# Single Stabilizing Point Mutation Enables High‐Resolution Co‐Crystal Structures of the Adenosine A_2A_ Receptor with Preladenant Conjugates

**DOI:** 10.1002/anie.202115545

**Published:** 2022-03-24

**Authors:** Tobias Claff, Tim A. Klapschinski, Udaya K. Tiruttani Subhramanyam, Victoria J. Vaaßen, Jonathan G. Schlegel, Christin Vielmuth, Jan H. Voß, Jörg Labahn, Christa E. Müller

**Affiliations:** ^1^ Pharmaceutical Institute Pharmaceutical & Medicinal Chemistry University of Bonn An der Immenburg 4 53121 Bonn Germany; ^2^ Centre for Structural Systems Biology (CSSB) Notkestraße 85 22607 Hamburg Germany; ^3^ Research Centre Jülich Institute of Complex Systems (IBI-7) Wilhelm-Johnen-Straße 52425 Jülich Germany

**Keywords:** Adenosine A_2A_ Receptor, Cancer, G Protein-Coupled Receptor (GPCR), Preladenant Conjugates, Protein Structures

## Abstract

The G protein‐coupled adenosine A_2A_ receptor (A_2A_AR) is an important new (potential) drug target in immuno‐oncology, and for neurodegenerative diseases. Preladenant and its derivatives belong to the most potent A_2A_AR antagonists displaying exceptional selectivity. While crystal structures of the human A_2A_AR have been solved, mostly using the A_2A_‐StaR2 protein that bears 9 point mutations, co‐crystallization with Preladenant derivatives has so far been elusive. We developed a new A_2A_AR construct harboring a single point mutation (S91^3.39^K) which renders it extremely thermostable. This allowed the co‐crystallization of two novel Preladenant derivatives, the polyethylene glycol‐conjugated (PEGylated) PSB‐2113, and the fluorophore‐labeled PSB‐2115. The obtained crystal structures (2.25 Å and 2.6 Å resolution) provide explanations for the high potency and selectivity of Preladenant derivatives. They represent the first crystal structures of a GPCR in complex with PEG‐ and fluorophore‐conjugated ligands. The applied strategy is predicted to be applicable to further class A GPCRs.

## Graphical abstract

A super‐stable adenosine A_2A_ receptor (A_2A_AR) containing a single point mutation in the transmembrane regions was developed by imitating an allosteric Na^+^ through introduction of a basic amino acid residue. This allowed the determination of high‐resolution co‐crystal structures with newly designed PEGylated and fluorophore‐labeled Preladenant derivatives, which belong to the most potent A_2A_AR antagonists endowed with outstanding selectivity.

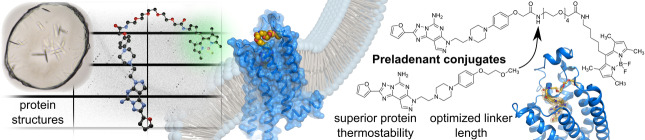

## Introduction

The nucleoside adenosine has been recognized as a fundamental signaling molecule of life.^[1]^ It activates a family of G protein‐coupled receptors (GPCRs) designated A_1_, A_2A_, A_2B_, and A_3_. The adenosine A_2A_ receptor (A_2A_AR) subtype plays a pivotal role in a variety of immunological processes. It couples to G_s_ proteins leading to an increase in intracellular cyclic adenosine monophosphate (cAMP) concentrations.^[2]^ Adenosine represents one of the strongest immunosuppressive agents of the innate immune system, an activity that is mainly mediated by activation of the A_2A_AR.[^3^, ^4^] This receptor acts as an immune checkpoint that is exploited by tumor cells to evade the immune system and to promote uncontrolled growth.^[5]^ While extracellular adenosine levels are typically in the nanomolar range, they can dramatically rise in the tumor microenvironment and in inflamed tissues by more than 100‐fold reaching micromolar concentrations.^[6]^ Blockade of A_2A_ARs re‐activates the compromised immune cells in the microenvironment of cancer cells thereby allowing, for example, T cell infiltration of tumor tissues.^[4]^ Thus, A_2A_AR antagonists represent a new, promising class of checkpoint inhibitors for the treatment of cancers and possibly also for the therapy of infections.[^7^, ^8^]

In the brain, the A_2A_AR is almost exclusively expressed in the caudate‐putamen (striatum) at high levels.^[9]^ Neurodegeneration was found to lead to an upsurge in A_2A_AR expression.^[10]^ Elevated A_2A_AR levels are already observed in early‐stage patients suffering from Parkinson's Disease (PD)^[11]^ and were found to correlate with the severity of PD.^[12]^

Preladenant (SCH‐420814, see Figure S1) was the first non‐xanthine A_2A_AR antagonist to enter clinical development for the treatment of PD.^[13]^ While the drug was found to be generally safe and well‐tolerated, phase III clinical trials failed to provide evidence for its efficacy,^[14]^ possibly due to an imperfect trial design. Nevertheless, Preladenant is one of the most potent A_2A_AR antagonists with an outstanding selectivity towards the other AR subtypes of several hundred‐ to more than 1000‐fold.^[15]^ The tricyclic Preladenant scaffold has therefore been utilized to develop tool compounds and labeled diagnostics, e.g. positron emission tomography tracers^[16]^ and fluorescence‐labeled derivatives.^[17]^

Although several high‐resolution crystal structures of the A_2A_AR were obtained, no structures in complex with Preladenant or its derivatives have been reported. Thus, the exact binding mode and interactions of this prominent and unique class of A_2A_AR antagonists are still unknown. In the last decade, advances in A_2A_AR structural biology were greatly facilitated by a research platform that introduced the stabilized receptor (StaR) A_2A_‐StaR2^[18]^ which had been engineered to achieve enhanced protein stability through multiple point mutations.^[19]^ The A_2A_‐StaR2 has been indispensable to enhance our understanding of A_2A_AR antagonist binding pockets. According to all protein data bank (PDB) (www.rcsb.org)^[20]^ entries, 18 different A_2A_AR antagonists have so far been crystallized in complex with the A_2A_AR (for an overview see Table S1). The vast majority of these ligands (16) was exclusively co‐crystallized using the A_2A_‐StaR2 either with or without the intracellular fusion protein bRIL (thermostabilized apocytochrome b_562_RIL).[^19^, ^21^] Moreover, a drug design program based on A_2A_‐StaR2 structures enabled the development of the potent A_2A_AR antagonist Imaradenant (AZD‐4635, see Figure S1, *K*_i_ A_2A_AR: 1.7 nM, 37‐fold selective versus the A_2B_AR).[^22^, ^23^] The A_2A_‐StaR2 construct comprises nine point mutations, two of which, T88^3.36^A and S277^7.42^A, are located inside the orthosteric ligand binding pocket of the A_2A_AR interfering with agonist binding^[24]^ and, in case of the S277^7.42^A mutation, possibly also with the binding of antagonist scaffolds^[25]^ (superscripts refer to the Ballesteros‐Weinstein system^[26]^). In fact, the recently solved crystal structure of the A_2A_‐StaR2 in complex with the clinical candidate Imaradenant^[23]^ revealed direct ligand contacts to the mutated A277^7.42^.

In an effort to strongly reduce the number of point mutations and, in particular, to avoid mutations located in the orthosteric ligand binding pocket, we developed a new, significantly improved thermostabilized A_2A_AR mutant harboring only a single point mutation (designated A_2A_‐PSB1‐bRIL) and yet endowed with superior stability compared to the A_2A_‐StaR2 mutant. This was inspired by a corresponding mutation in the crystallized serotonin 5‐HT_2A_ receptor which appeared to show promise for the A_2A_AR as well.[^27^, ^28^]

In parallel, we developed a new series of Preladenant derivatives equipped with polyethylene glycol (PEG) linkers of different length appropriate for connecting reporter molecules, e.g. fluorescent dyes. An optimized PEGylated Preladenant derivative, PSB‐2113, was subsequently labeled with a boron‐dipyrromethene (BODIPY) fluorophore yielding the fluorescent probe PSB‐2115 suitable for specific A_2A_AR imaging.

Herein, we present the first high‐resolution crystal structure of A_2A_‐PSB1‐bRIL in complex with the Preladenant conjugates PSB‐2113 and PSB‐2115 at 2.25 Å and 2.6 Å resolution, respectively. Our results provide insights into the interactions of the potent and highly selective Preladenant scaffold with the orthosteric binding site of the receptor. Moreover, we obtained the first X‐ray structures of a GPCR co‐crystallized with an antagonist that is conjugated to a PEG linker and a fluorescent dye.

## Results and Discussion

**Figure 1:**
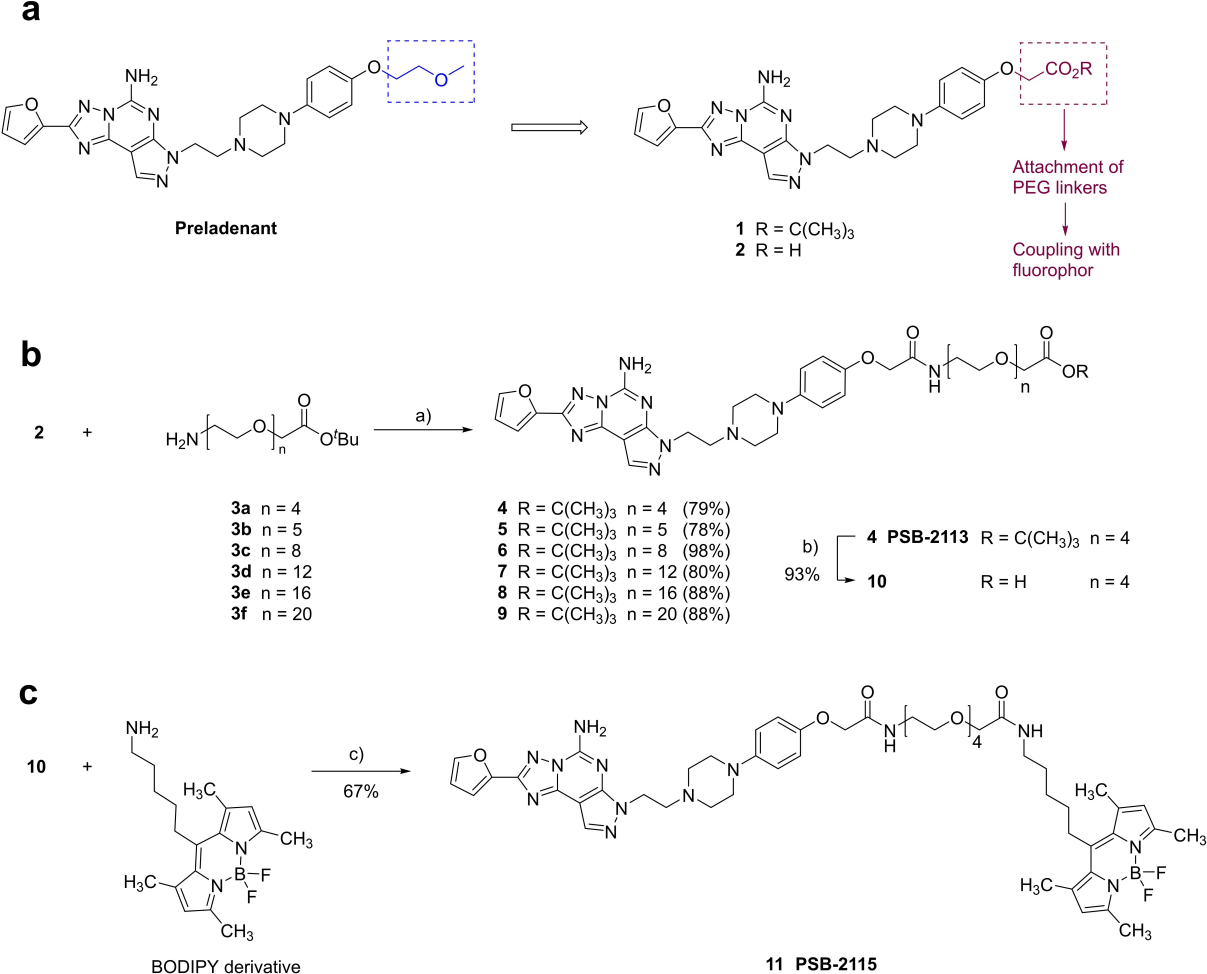
Design and synthesis of conjugated Preladenant derivatives. a) Design and synthetic strategy. b) Synthesis of PEGylated Preladenant derivatives. c) Synthesis of Preladenant derivative labeled with a BODIPY fluorophore attached via an optimized PEG linker. Reaction conditions: a) HATU, DIPEA, CH_2_Cl_2_, RT, 24 h. b) trifluoroacetic acid, TIPS, CH_2_Cl_2_, RT, 24 h. c) HATU, DIPEA, CH_2_Cl_2_, RT, 24 h.

As a first step, we synthesized novel conjugated Preladenant derivatives. This was achieved by replacement of the terminal methoxyethyl ether group on the extended phenylpiperazinylethyl residue of Preladenant that is attached to the pyrazole ring of the tricyclic core structure (see Figure 1).

A synthetic strategy to obtain the target compounds was designed as depicted in Figure 1a. The carboxy‐functionalized Preladenant derivative **2** was prepared via its protected precursor **1** (details on the synthesis of compounds **1** and **2** are provided in Scheme S1). Carboxylic acid **2** can subsequently be coupled with amines to connect PEG linkers to the pharmacophore via amide formation. To this end, *tert*‐butyloxycarbonyl(Boc)‐protected PEG linkers of increasing length (4 to 20 ethylene glycol monomer units, **3 a**–**3 f**) were attached to compound **1** using (1‐[bis(dimethylamino)methylene]‐1*H*‐1,2,3‐triazolo[4,5‐*b*]pyridinium‐3‐oxide hexafluorophosphate (HATU) as a coupling reagent in the presence of diisopropylethylamine (DIPEA) as a base under mild conditions (see Figure 1b). Products **4**–**9** were obtained in excellent yields (see Figure 1). These were subsequently tested in radioligand competition binding assays to determine A_2A_AR affinities and selectivities versus the other human AR subtypes (see Table 1). Our aim at this point was to study the consequences of the introduced structural modifications on the Preladenant scaffold, and to find out which linker length would be optimal. While the free carboxylic acid **2**, used as a precursor for the coupling reactions, showed only moderate A_2A_AR affinity (*K*_i_ 200 nM), its Boc‐protected ester **1** was ≈100‐fold more potent displaying similar affinity as the parent compound Preladenant (Table 1). All investigated Boc‐protected PEG derivatives (**4**–**9**) exhibited higher affinity for the A_2A_AR than the carboxylate precursor **2**. Increasing PEG linker length resulted in decreased A_2A_AR affinity. In fact, the highest A_2A_AR affinity was achieved with the shortest PEG linker comprised of four ethyleneglycol units (compound **4**, PSB‐2113, *K*_i_ 2.28 nM). Therefore, we selected the PEG‐substituted compound **4** for subsequent studies. Deprotection with trifluoroacetic acid in the presence of triisopropylsilane (TIPS) led to carboxylic acid **10** (*K*_i_ A_2A_AR 8.84 nM) in high yield. Subsequent coupling reaction with an aminoalkyl‐functionalized BODIPY derivative, prepared as previously described,^[29]^ in the presence of HATU/DIPEA under mild conditions yielded the desired BODIPY‐labeled Preladenant derivative **11** (PSB‐2115) in excellent yield (see Figure 1c). The final BODIPY‐labeled product still showed very high affinity for the A_2A_AR (*K*_i_ 3.47 nM). This is combined with excellent selectivity (>1000‐fold) versus the A_2B_‐ and A_3_AR subtypes, and still around 50‐fold selectivity versus the A_1_AR (see Table 1). Moreover, PEGylation can be expected to increase water‐solubility and modulate pharmacokinetic properties.^[30]^ For example, it will prevent brain penetration and associated side‐effects, such as central stimulation which is undesired for peripheral indications, e.g. in immuno‐oncology and in the treatment of infections. Moreover, it allows the attachment of targeting moieties, e.g. antibodies, and reporter groups such as fluorophores as in PSB‐2115.

**Table 1:** Affinities of Preladenant derivatives at human adenosine receptor subtypes.^[a]^

Compound	Human A_1_AR	Human A_2A_AR	Human A_2B_AR	Human A_3_AR
	Radioligand [^3^H]CCPA *K*_i_±SEM [nM] (or % inhibition±SEM at 1 μM)	Radioligand [^3^H]MSX‐2 *K*_i_±SEM [nM]	Radioligand [^3^H]PSB‐603 *K*_i_±SEM [nM] (or % inhibition±SEM at 1 μM)	Radioligand [^3^H]PSB‐11 *K*_i_±SEM [nM] (or % inhibition±SEM at 1 μM)
**ZM241385^[b]^**	**225**	**0.8**	**50**	**>10 000**
**Preladenant^[c]^**	**295±**10	**0.884±**0.232	**>1000**	**>1000**
**1**	**420±**36	**1.93±**0.75	**>1000** (15±10)	**>1000** (25±2)
**2**	**>1000** (18±4)	**200±**16	**>1000** (2±11)	**>1000** (12±10)
**4 (PSB‐2113)**	**>1000** (38±9)	**2.28±**0.41	**>1000** (9±1)	**>1000** (34±4)
**5**	**>1000** (28±1)	**9.39±**1.39	**>1000** (24±1)	**>1000** (8±5)
**6**	**>1000** (1±6)	**10.3±**2.1	**>1000** (0±3)	**>1000** (26±4)
**7**	**>1000** (23±9)	**8.92±**4.05	**>1000** (8±3)	**>1000** (14±5)
**8**	**>1000** (2±2)	**30.3±**7.9	**>1000** (5±2)	**>1000** (2±0)
**9**	**>1000** (0±12)	**45.5±**12.3	**>1000** (−8±2)	**>1000** (2±5)
**10**	**>1000** (6±5)	**8.84±**0.64	**>1000** (17±9)	**>1000** (13±1)
**11 (PSB‐2115)**	**165±**20	**3.47±**0.23	**>1000** (32±9)	**>1000** (39±6)

[a] *K*_i_ values are means from 3 independent experiments shown in bold±standard error of the mean (SEM). [b] See ref. [31], for structure see Figure S1. [c] See ref. [15].

With these highly potent and selective Preladenant conjugates in hand we aimed at obtaining co‐crystal structures in complex with the human A_2A_AR to gain insight into their interactions with the receptor protein.

Initially, we attempted to crystallize the human A_2A_AR in complex with the new Preladenant conjugates using the previously described A_2A_AR crystallization construct^[32]^ that lacks the long A_2A_AR *C*‐terminal tail and in which the intracellular loop (ICL) 3 is replaced by the soluble fusion protein bRIL (designated A_2A_‐ΔC‐bRIL). This construct does not contain any additional stabilizing point mutations. While we accomplished to produce crystals with an average size of 50 μm (Figure S2A), no high‐resolution diffraction data could be obtained. Our observation is consistent with previous studies reporting only low‐resolution diffraction data or micro‐crystal hits deriving from co‐crystallization of the same A_2A_AR protein with the related tricyclic A_2A_AR antagonists SCH‐442416 and SCH‐58261^[33]^ (for compound structures see Figure S1). To date, 17 crystal structures of A_2A_‐ΔC‐bRIL in complex with the structurally related bicyclic A_2A_AR antagonist ZM241385 have been obtained. However, the same strategy does not appear to be as straightforward for tricyclic A_2A_AR antagonists like Preladenant. A plausible explanation could be differences in ligand binding kinetics or inverse agonist efficacies.^[34]^

**Figure 2:**
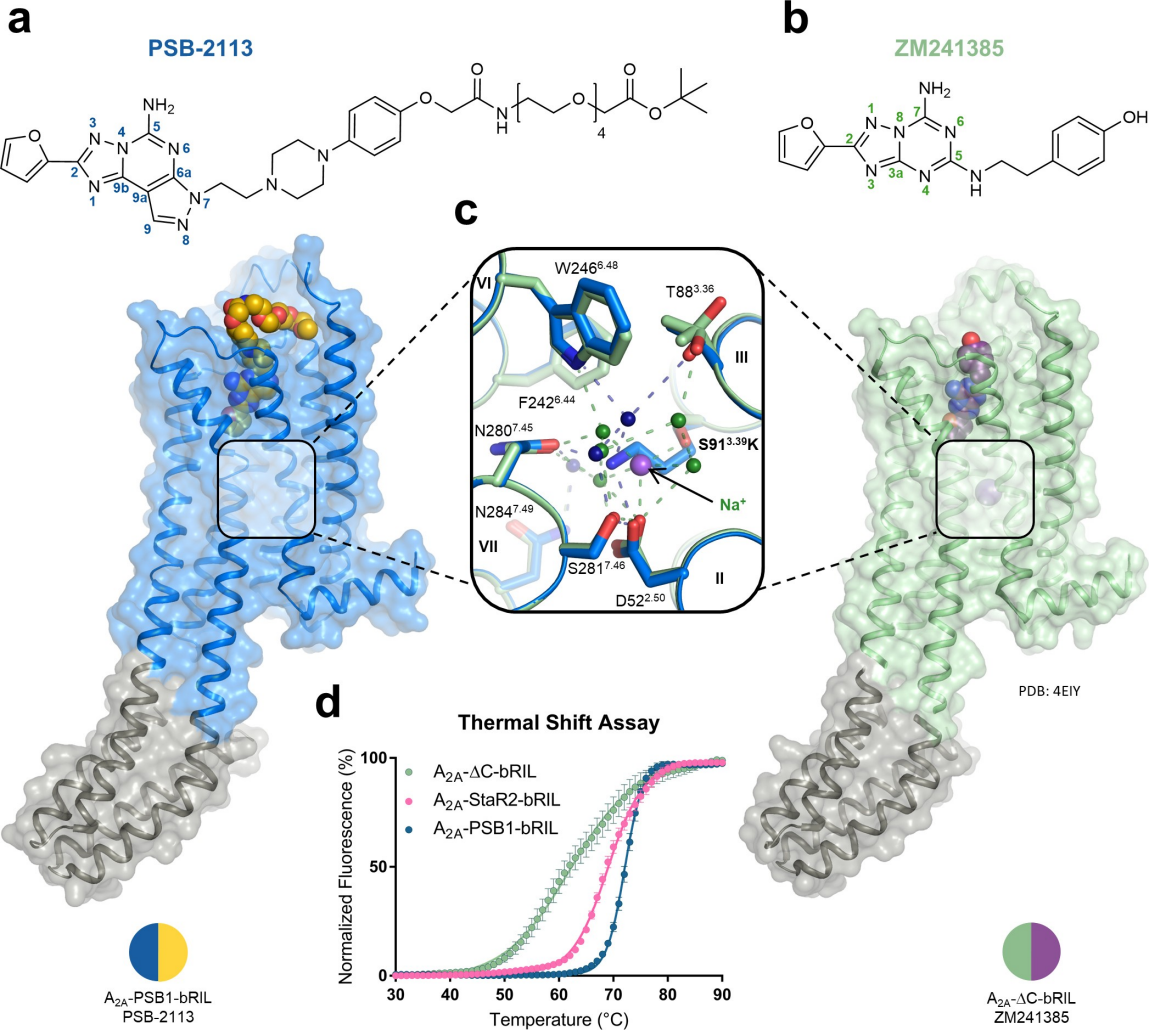
Architecture of the single‐mutated thermostabilized A_2A_AR. Overview of the crystal structures and A_2A_AR antagonists of a) A_2A_‐PSB1‐bRIL‐PSB‐2113 compared to b) A_2A_‐ΔC‐bRIL‐ZM241385. c) Sodium binding pocket comparison between A_2A_‐ΔC‐bRIL and A_2A_‐PSB1‐bRIL highlighting the introduced S91^3.39^K mutation. d) Thermostability assessment of different A_2A_AR crystallization constructs without the presence of A_2A_AR ligands. Error bars indicate the SEM.

More stable A_2A_AR crystallization constructs have meanwhile become available, the most successful one being the A_2A_‐StaR2 mutant that contains nine point mutations.^[19]^ Rather than utilizing the A_2A_‐StaR2 for crystallization, our objective was to keep the number of mutations at a minimum, and, importantly, to avoid any mutations that may interfere with ligand binding. Inspired by the recently elucidated crystal structure of the serotonin 5‐HT_2A_ receptor^[27]^ where the basic amino acid lysine occupies the well‐known allosteric sodium binding site,^[35]^ we introduced a single point mutation into the A_2A_AR construct A_2A_‐ΔC‐bRIL at the analogous position to replace the corresponding serine residue S91^3.39^ by lysine (S91^3.39^K). The S91^3.39^K mutation appeared to be in fact beneficial for A_2A_AR stability.^[28]^ This A_2A_AR mutant, designated A_2A_‐PSB1‐bRIL (PSB, Pharmaceutical Sciences Bonn), led to substantial protein thermostabilization, even in the ligand‐free (APO) state, consistent with a melting temperature (*T*_M_) increase by approximately 10 °C compared to A_2A_‐ΔC‐bRIL (see Figure 2). In fact, the thermostability of A_2A_‐PSB1‐bRIL was significantly higher than the thermostability of the A_2A_‐StaR2‐bRIL that was concurrently produced in our laboratory and purified in parallel with the new construct using the same procedure (Δ*T*_M_=3.03 °C; *p*=0.0025, two‐sided t‐test). The resulting new thermostabilized construct, designated A_2A_‐PSB1‐bRIL, was expressed in and purified from *Spodoptera frugiperda* (Sf9) insect cells. We succeeded in obtaining A_2A_‐PSB1‐bRIL‐ligand complexes with high purity (Figure S2B and C) and successfully crystallized them in lipidic cubic phase (LCP) (Figure S2D and E). Importantly, protein crystals of A_2A_‐PSB1‐bRIL produced high‐resolution diffraction data which enabled the elucidation of two new crystal structures in complex with PSB‐2113 and PSB‐2115 (see Table S2 for detailed refinement statistics).

The root‐mean‐square‐deviation (RMSD) of all resolved GPCR backbone atoms between A_2A_‐PSB1‐bRIL and A_2A_‐ΔC‐bRIL (PDB 4EIY) is 0.183 Å (1204 aligned atoms, based on the PSB‐2113 complex) indicating that the transmembrane helix geometry is not affected by the newly introduced S91^3.39^K mutation. The respective wild‐type (wt) residue in this position (S91^3.39^) is located inside the highly conserved allosteric sodium binding pocket, where it directly coordinates a sodium ion as observed in many inactive state class A GPCRs.[^32^, ^35^] In the novel mutant, the larger lysine in this position displaces the sodium ion together with three structural water molecules, and fully occupies the former allosteric binding pocket without disrupting the overall helix geometry of the A_2A_AR (Figure 2a, b and c). In fact, the protonated amino group of K91^3.39^ mimics the positively charged sodium ion, thereby stabilizing the same inactive receptor conformation. Precisely, K91^3.39^ forms a salt bridge to D52^2.50^, a direct hydrogen bond interaction to N280^7.45^ and water‐mediated hydrogen bonds to S281^7.46^ and W246^6.48^ (Figure 2c). Thus, the long K91^3.39^ sidechain sterically prevents the activation‐induced collapse of the former sodium binding pocket^[24]^ and restricts the “rotamer toggle switch”,^[36]^ including amino acids T88^3.36^, F242^6.44^ and W246^6.48^, in the inactive conformation (Figure 2c).

**Figure 3:**
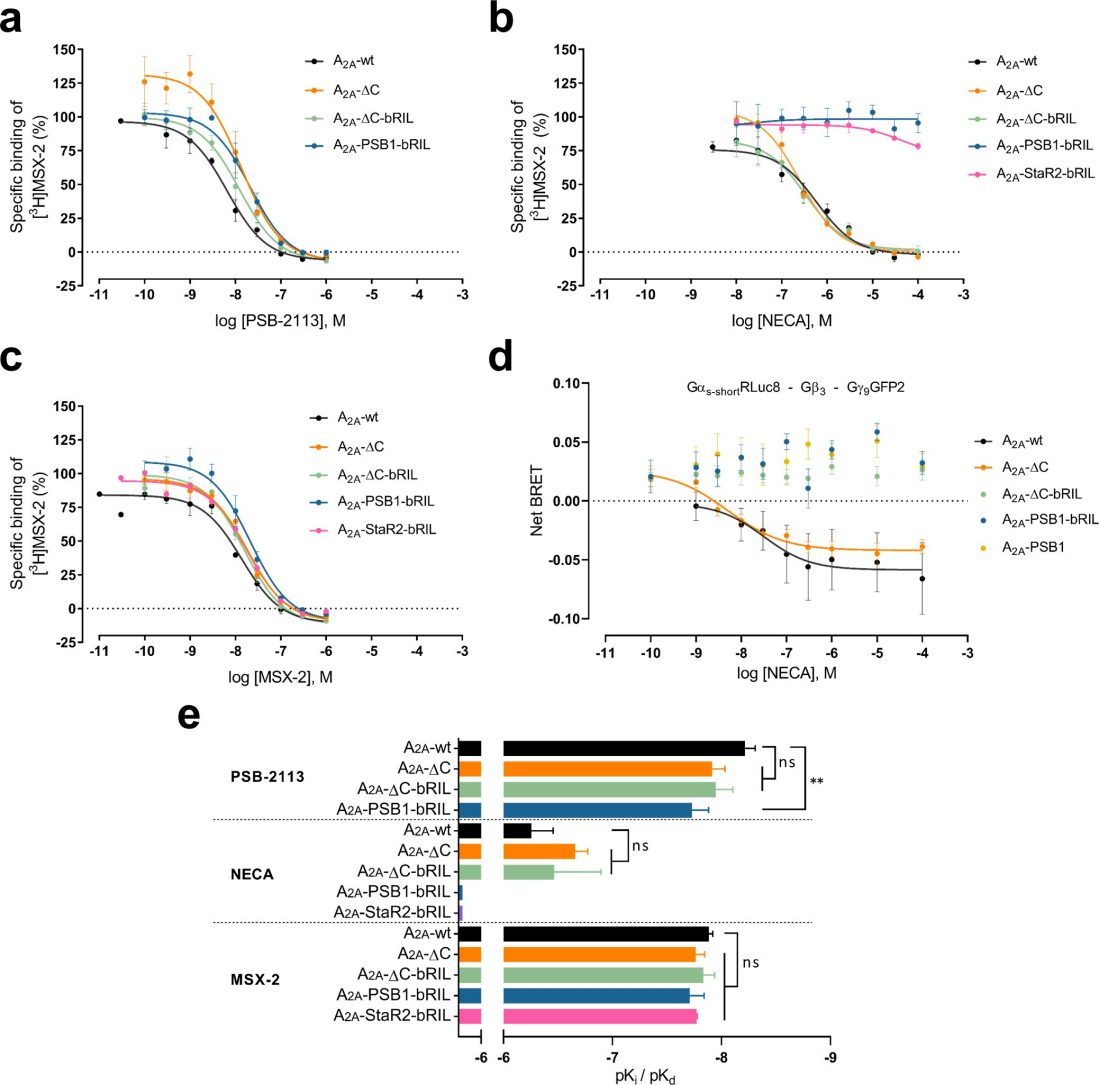
Pharmacological characterization of A_2A_AR constructs. Results of competitive radioligand binding experiments on Sf9 insect cell membranes with a) PSB‐2113, b) NECA and c) MSX‐2 using [^3^H]MSX‐2 as radioligand. Error bars indicate SEM. d) TRUPATH assay results using HEK293 cells expressing Gα_s‐short_Rluc8, Gβ_3_, Gγ_9_GFP2 and the respective A_2A_AR construct with error bars indicating SEM. e) Comparison of p*K*_i_ and p*K*_d_ values calculated from radioligand binding experiments with error bars indicating the standard deviation (SD). The statistical evaluation was performed using the one‐way‐ANOVA with Dunnett's post‐hoc test.

Radioligand binding experiments were performed with Sf9 insect cell membranes expressing A_2A_‐PSB1‐bRIL using the A_2A_‐selective antagonist radioligand [^3^H]MSX‐2.^[37]^ For comparison, various other A_2A_AR constructs were additionally investigated. For the wt A_2A_AR, radioligand binding experiments were further performed on membranes from Chinese hamster ovary (CHO−S) suspension cells. The affinity of the Preladenant conjugate PSB‐2113 to the wt A_2A_AR was virtually identical regardless of the cell line, CHO−S cells or Sf9 insect cells, in which the receptor was expressed (*K*_i_ 2.28 nM vs. 6.30 nM). Moreover, the new, PEGylated A_2A_AR antagonist PSB‐2113 as well as the standard xanthine antagonist MSX‐2 (for structure see Figure S1) were binding to the non‐mutated A_2A_‐ΔC‐bRIL and A_2A_‐ΔC with the same affinities as to the wt A_2A_AR (Figure 3 and Table S3). This demonstrates that A_2A_AR antagonist binding was neither altered by introduction of the bRIL fusion protein nor by truncation of the C‐terminus. The binding affinity of MSX‐2 to the S91^3.39^K‐mutated A_2A_‐PSB1‐bRIL receptor was also unaltered as compared to the wt A_2A_AR, while the affinity of PSB‐2113 was slightly (≈3‐fold) lower at the mutant than at the wt A_2A_AR, but still in the low nanomolar range (19.6 nM vs. 6.30 nM; *p*=0.0801; paired t‐test) (Figure 3 and Table S3). The S91^3.39^K mutation stabilizes the same inactive state as sodium ions. Since high sodium concentrations do not alter the affinity of A_2A_AR antagonists,^[32]^ we cannot expect an affinity increase towards A_2A_‐PSB1‐bRIL either.^[32]^ On the other hand, it has been shown that Preladenant and other antagonists bind to active state‐stabilized A_2A_AR constructs with significantly lower affinity.^[24]^

Moreover, we observed that the agonist 5′‐*N*‐ethylcarboxamidoadenosine (NECA) could still bind to the truncated but non‐mutated A_2A_AR constructs regardless of the presence of the fusion partner in the ICL3 (A_2A_‐ΔC and A_2A_‐ΔC‐bRIL) with similar affinity as to the wt A_2A_AR (Figure 3). However, no agonist binding to A_2A_‐PSB1‐bRIL could be detected (p*K*_i_<4.0) as exemplarily shown for NECA versus [^3^H]MSX‐2 (Figure 3 and Table S3). A rationale for the observed abolished agonist binding to A_2A_‐PSB1‐bRIL may be provided by the fact that the S91^3.39^K mutation restrains key activation switches in the inactive conformation. This prevents movements of W246^6.48^, H250^6.52^ and helix III that are required to accommodate the ribose moiety of A_2A_AR agonists (adenosine and its derivatives) in the ligand binding pocket.^[38]^ In our hands, NECA binding to the A_2A_‐StaR2‐bRIL was equally abolished.

Next, we utilized the biosensor platform TRUPATH^[39]^ to test the effect of the S91^3.39^K mutation on Gα_s_ activation. For this purpose, we stimulated the truncated A_2A_AR constructs with or without bRIL applying the agonist NECA. A_2A_‐ΔC‐bRIL served as a negative control since the fusion partner in the ICL3 sterically blocks the G protein binding site. In support of our findings from radioligand binding experiments, the results showed that the S91^3.39^K mutated A_2A_AR was not able to activate Gα_s_ proteins in HEK293 cells. On the other hand, Gα_s_ activation was unaffected in the *C*‐terminal truncated A_2A_AR construct when compared to the wt A_2A_AR (Figure 3d and Table S3).

**Figure 4:**
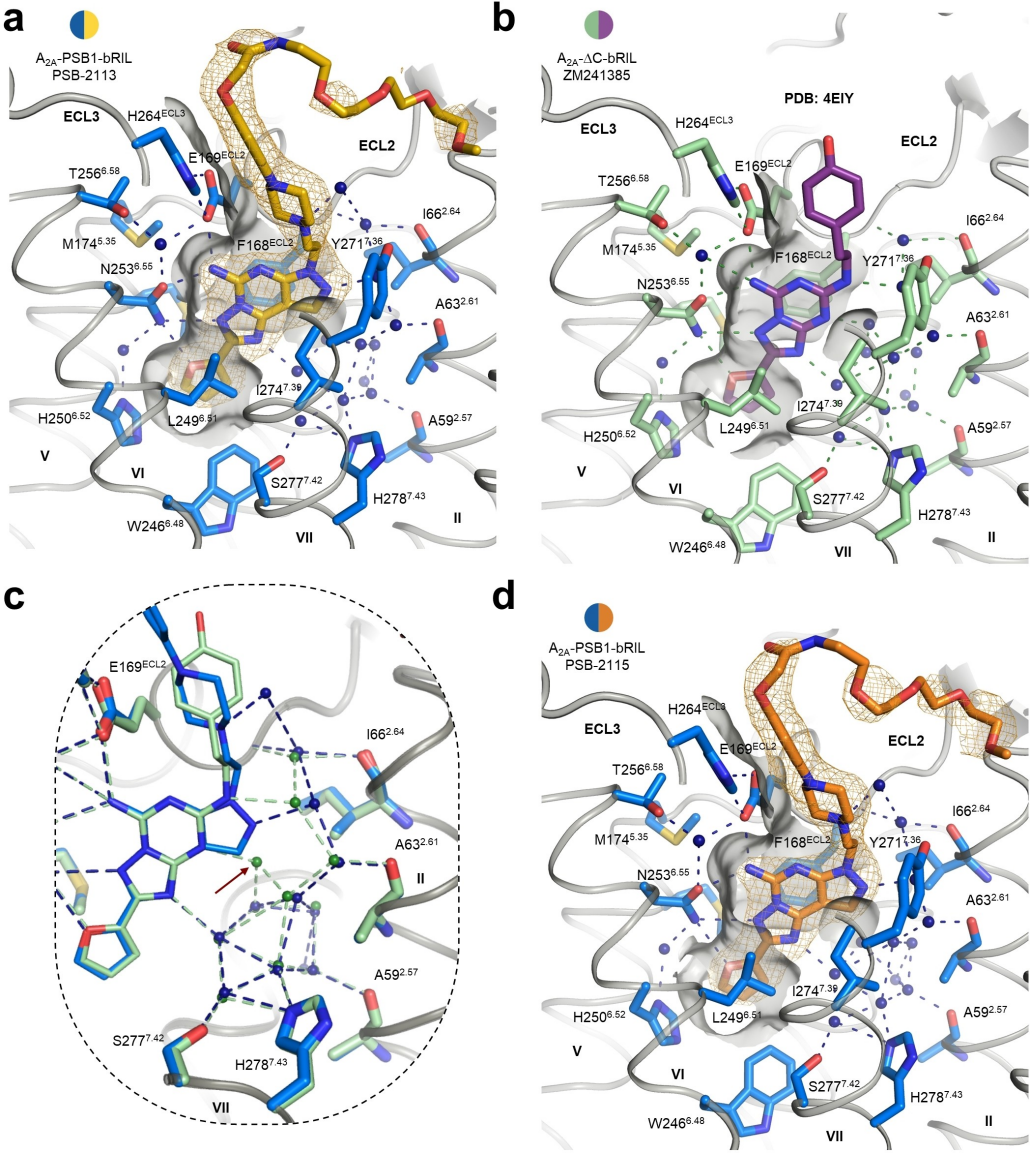
Comparison of ligand binding pockets. a) Ligand binding pocket of A_2A_‐PSB1‐bRIL‐PSB‐2113. The 2*F*_o_−*F*_c_ electron density of PSB‐2113 is shown in yellow mesh (contoured at 1.0 σ). b) Ligand binding pocket of A_2A_‐ΔC‐bRIL‐ZM241385. Coordinates were extracted from PDB entry 4EIY. c) Comparison of the water networks in A_2A_‐PSB1‐bRIL‐PSB‐2113 (blue) and A_2A_‐ΔC‐bRIL‐ZM241385 (green). The red arrow points to the structural water molecule that is displaced from the ligand binding pocket by the tricyclic core scaffold. d) Ligand binding pocket of A_2A_‐PSB1‐bRIL‐PSB‐2115. The 2*F*_o_−*F*_c_ electron density of PSB‐2115 is shown in orange mesh (contoured at 1.0 σ).

The core scaffold of Preladenant and its derivatives PSB‐2113 and PSB‐2115 exhibits certain similarities but also significant differences to the structurally well‐investigated A_2A_AR antagonist ZM241385 (for structures see Figure S1).[^32^, ^40^] Both antagonists contain an aromatic ring system that is connected to a 2‐furanyl moiety. However, while ZM241385 carries a bicyclic aromatic system, Preladenant possesses an additional five‐membered ring that likely contributes to its high selectivity compared to ZM241385. Despite the sterically more demanding tricyclic core, the Preladenant derivative PSB‐2113 binds to the A_2A_AR in the same orientation as ZM241385 and shows similar direct ligand interactions to helices V, VI, VII and extracellular loop (ECL) 2 (Figure 4a and b).

This includes a key hydrogen bond network to N253^6.55^ and E169^ECL2^ by the furan oxygen atom and the 5‐amino group of the heterocyclic core. In addition, the tricyclic aromatic system is stabilized by π–π stacking to F168^ECL2^ and by hydrophobic contacts to L249^6.51^ and I274^7.39^ (Figure 4a). PSB‐2113 is connected to helices I, II, III, and VII via water‐mediated hydrogen bonds, similarly as observed for ZM241385.^[32]^ However, the tricyclic core of PSB‐2113 extends further towards helix II which leads to the displacement of one of the structural water molecules from the ligand binding pocket (Figure 4c). The water molecules in this particular water network were previously termed “unhappy waters”^[41]^ as they would prefer to be in the bulk solvent but cannot leave a vacuum behind. Hence, the displacement of the water molecule by PSB‐2113 from the ligand binding pocket would be expected to be energetically favorable and is likely one of the reasons for the compound's high affinity. Moreover, while the number of nitrogen atoms is identical in the core scaffold of PSB‐2113 and ZM241385, their altered position (compare *N*7 and N8 in PSB‐2113 with *N*4 and *N* ^5^ in ZM241385, Figure 2a and b) results in a different pattern of hydrogen bond donors and acceptors. Specifically, PSB‐2113 does neither possess a hydrogen bond acceptor in position 9a nor a hydrogen bond donor in the *N*7‐position due to the additional five‐membered ring. This leads to small positional movements of water molecules within the hydrogen bonding network (Figure 4c) but does not interfere with the overall system that connects the ligand to the backbone of helices II and III and the sidechains of E13^1.39^, Y271^7.36^, S277^7.42^, and H278^7.43^ (Figure 4a and c). The phenylpiperazinylethyl moiety that is attached to the N7 in PSB‐2113 extends towards the extracellular surface of the A_2A_AR, stabilized by π–π stacking to H264^ECL3^ (Figure 4a). A similar binding mode was previously determined for the A_2A_AR antagonist 12x that also features a phenylpiperazinylethyl extension but is derived from ZM241385 (Figure S3).^[21]^ H264^ECL3^ itself forms an ionic lock with E169^ECL2^ that has frequently been observed in both active and inactive state A_2A_AR structures.^[42]^ Structures of the A_2A_AR lacking the ionic lock have also been obtained but appear to be dependent on either crystallization conditions^[19]^ or the co‐crystallized ligand (Table S1).^[43]^ No unambiguous electron density evidence could be observed for the PEG linker that clearly sticks out of the binding pocket (Figure 4a). This indicates that the PEG‐chain located at the receptor surface is highly flexible, which is a desired characteristic for the intended purpose to attach variable reporter molecules to the terminus of the linker.

**Figure 5:**
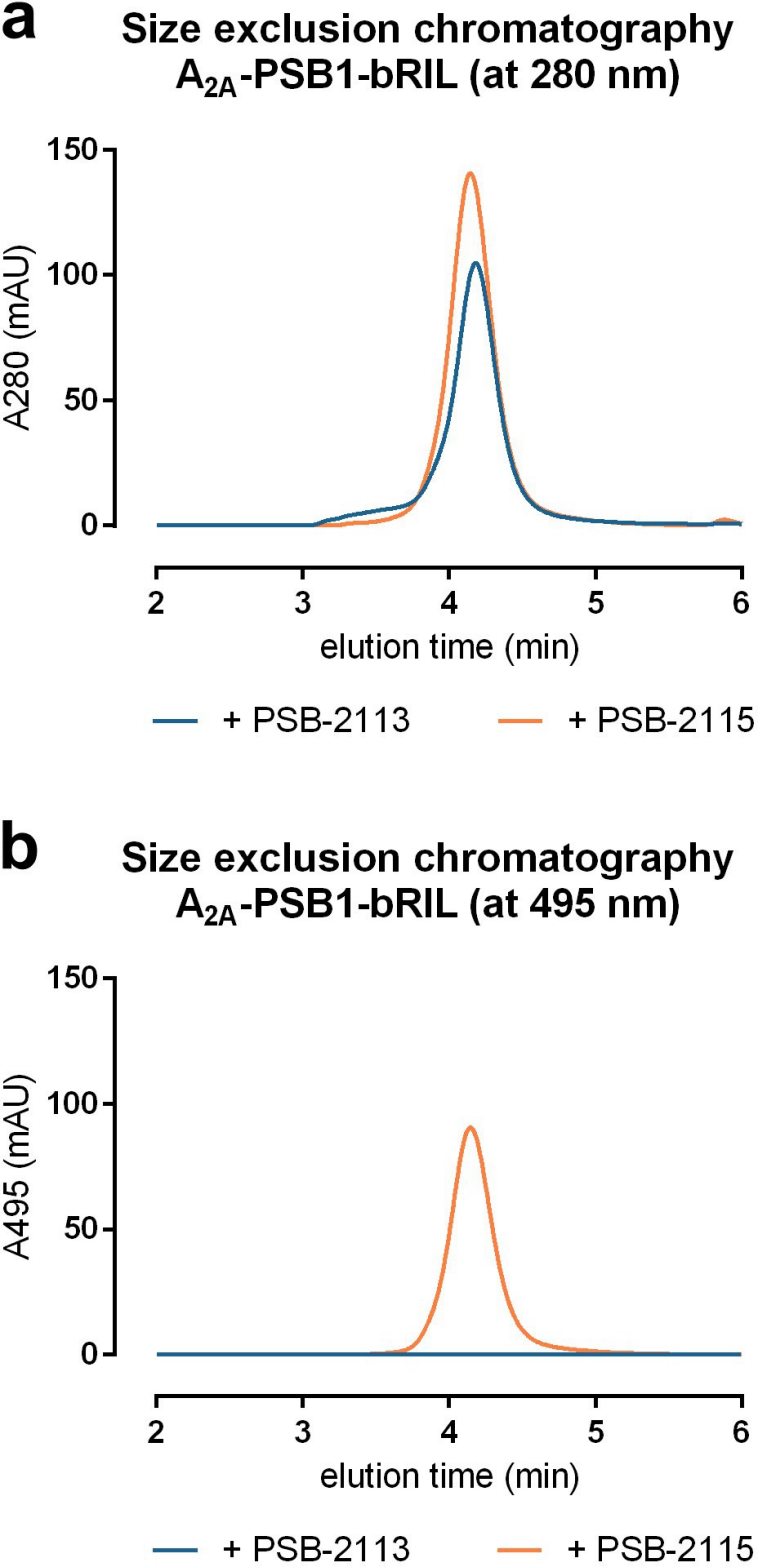
Size‐exclusion chromatography analysis. The complexes of A_2A_AR antagonists PSB‐2113 and PSB‐2115 together with A_2A_‐PSB1‐bRIL were analyzed by size‐exclusion chromatography using two different detection wavelengths (a) 280 nm and b) 495 nm).

Next, we solved the crystal structure of the A_2A_AR in complex with the new fluorescence‐labeled A_2A_AR antagonist PSB‐2115. This ligand differs from PSB‐2113 by the attached BODIPY fluorophore (Figure S1). The binding pocket that accommodates the Preladenant scaffold is virtually identical in both structures (Figure 4a and d), proving that the attached fluorophore does not interfere with A_2A_AR binding. In analogy to PSB‐2113, no electron density could be observed neither for the flexible PEG linker, nor for the BODIPY fluorophore, and no specific interactions of the A_2A_AR with the linker or fluorophore could be detected. Analytical size‐exclusion chromatography confirmed the presence of the fluorophore in the A_2A_‐PSB1‐bRIL‐PSB‐2115 complex (Figure 5). A signal could be observed for the latter complex at the absorption maximum of the respective BODIPY derivative (495 nm, for the fluorescence spectrum see Figure S4), whereas the analogous PSB‐2113 complex that is lacking the fluorophore was only detectable at a lower, protein‐specific wavelength (280 nm).

**Figure 6:**
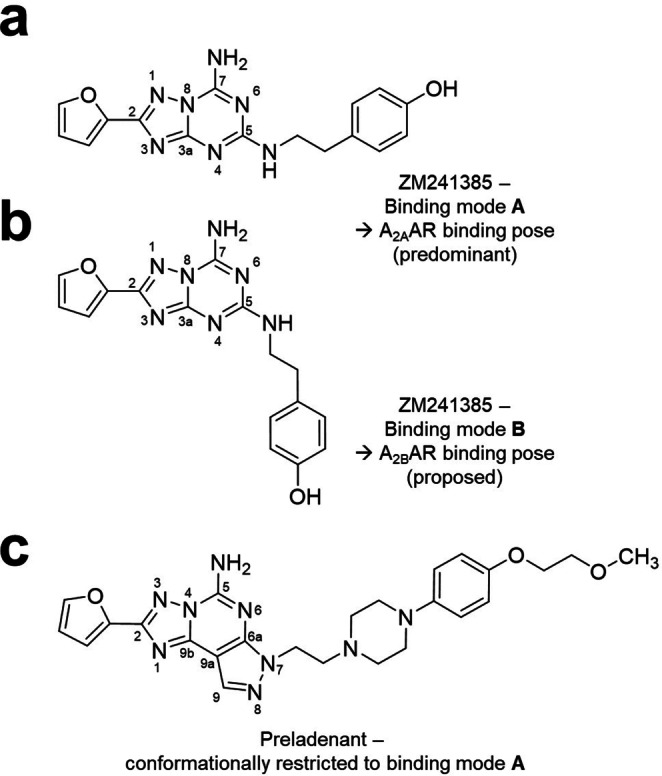
a) Binding pose **A** of ZM241385 to the A_2A_AR as seen in PDB ID 4EIY. b) Proposed binding pose **B** of ZM241385 in the A_2B_AR.^[45]^ c) Binding mode of the Preladenant scaffold as observed in the new A_2A_AR structures.

In contrast to the tricyclic Preladenant and its new conjugates, which show high selectivity for the A_2A_AR, the previously co‐crystallized bicyclic antagonist ZM241385 is only weakly selective, binding additionally to the A_2B_AR with high affinity.^[44]^ The new crystal structures suggest that the tricyclic core and the resulting conformational restriction of the substituent at the *N*7‐position of Preladenant represent important determinants for A_2A_AR selectivity. To date, no A_2B_AR structures have yet been solved. However, homology modeling approaches have proposed structural features of the A_2B_AR and its orthosteric ligand binding site.^[45]^ The extracellular amino‐terminus and loops differ significantly between the A_2A_‐ and the A_2B_AR whereas the amino acids in the orthosteric ligand binding pocket of both receptor subtypes are nearly identical with only one single amino acid difference (L249^6.51^ in the A_2A_AR and V250^6.51^ in the A_2B_AR). The leucine residue in position 249^6.51^ of the A_2A_AR exhibits direct hydrophobic contacts to the tricyclic Preladenant structure as observed in our newly determined structures (Figure 4a). Moreover, an L249^6.51^V mutation in the A_2A_AR has been shown to lower the binding affinity of ZM241385.^[46]^ Hence, its exchange to valine in the A_2B_AR may contribute to the observed high A_2A_AR selectivity of Preladenant and its derivatives. Moreover, the additional pyrazole ring in Preladenant determines the direction of the elongated *N*7‐substituent, whose conformation is thereby restricted, i.e. the exit vector is sterically fixed (see Figure 6). In contrast, the phenylethyl residue attached to the analogous *N* ^5^ (the amino group attached to C5) in the non‐selective bicyclic antagonist ZM241385 is much more flexible and therefore able to adopt different conformations, e.g. conformation **A**, similar to Preladenant (Figure 6) or conformation **B**, in which the phenyethyl residues points into a completely different direction. Conformation **A** of the *N* ^5^‐substituent in ZM241385 is consistent with the predominant A_2A_AR binding mode^[32]^ and with the fixed conformation in Preladenant. However, a structure of the A_2A_‐StaR2 in complex with ZM241385,^[19]^ crystallized by vapor‐diffusion in alkaline conditions, showed that the A_2A_AR can also harbor binding mode **B**, and is thus able to accommodate both conformations. On the other hand, previous molecular docking experiments suggested binding mode **B** for ZM241385 in the A_2B_AR binding pocket^[45]^ and we propose that binding mode **A** would lead to a sterical clash with A_2B_AR residues at the extracellular terminus of its helix VII (e.g. K269^7.32^). The fact that Preladenant analogs substituted at *N*8 rather than *N*7, can, in contrast, display high A_2B_AR affinity,^[47]^ further supports our hypothesis. Shifting of the large residue in Preladenant from the *N*7‐ to the *N*8‐position will allow it to adopt a conformation that can now interact with both the A_2B_‐ and the A_2A_AR binding pocket.

## Conclusion

The A_2A_AR has become an important drug target.[^7^, ^8^, ^22^] In particular, A_2A_AR antagonists are being developed for the treatment of neurodegenerative diseases and for cancer therapy due to their immunostimulatory and anti‐proliferative effects. Extensive efforts have been invested in studying the A_2A_AR's structure in complex with various ligands.[^19^, ^21^, ^24^, ^32^] Nevertheless, a co‐crystal structure of one of the most potent (*K*_i_<1 nM) and selective (≈3 orders of magnitude) A_2A_AR antagonists, Preladenant, has not been accessible to date. We have now been able to solve A_2A_AR crystal structures in complex with two Preladenant derivatives, PSB‐2113 and PSB‐2115. This has been possible due to the design and construction of the novel thermostabilized A_2A_AR mutant A_2A_‐PSB1‐bRIL, which harbors only a single, but crucial point mutation in the transmembrane domain. Although we achieved a marked decrease in the number of mutated amino acid residues (with only a single exchange) compared to the previously optimized A_2A_AR crystallization construct (with nine mutations),^[19]^ the stability of the novel construct is even greater than that of any other A_2A_AR mutant reported to date. Thus, the A_2A_‐PSB1‐bRIL receptor construct is proposed to become the new gold standard for the determination of A_2A_AR structures in its inactive state, which will be most helpful for the development of novel A_2A_AR blockers. The A_2A_AR is being used as a test case for class A GPCRs in general, and we predict that our strategy for GPCR stabilization should be useful for many other GPCRs that are modulated in the same way by sodium ions as the A_2A_AR. The newly developed PEGylated and fluorescence‐labeled Preladenant derivatives represent prototypes of valuable and versatile pharmacological tools for studying this (patho)physiologically important receptor and drug target. Their high‐resolution X‐ray structures will guide the way to improved A_2A_AR antagonists which have great potential as novel drugs for diseases with urgent medical need, such as neurodegeneration and cancer.

## Conflict of interest

The authors declare no conflict of interest.

1

## Supporting information

As a service to our authors and readers, this journal provides supporting information supplied by the authors. Such materials are peer reviewed and may be re‐organized for online delivery, but are not copy‐edited or typeset. Technical support issues arising from supporting information (other than missing files) should be addressed to the authors.

Supporting Information

## Data Availability

The data that support the findings of this study are available in the supplementary material of this article. Coordinates and structure factors have been deposited in the Protein Data Bank (PDB) under accession codes 7PX4 (A_2A_‐PSB1‐bRIL‐PSB‐2113) and 7PYR (A_2A_‐PSB1‐bRIL‐PSB‐2115).

## References

[1a] G. Burnstock , Nat. Rev. Drug Discovery 2008, 7, 575–590; 18591979 10.1038/nrd2605

[1b] B. Fredholm , A. Verkhratsky , Acta Physiol. 2010, 199, 91–92. 10.1111/j.1748-1716.2010.02113.x 20534039

[2a] P. A. Borea , S. Gessi , S. Merighi , F. Vincenzi , K. Varani , Physiol. Rev. 2018, 98, 1591–1625; 29848236 10.1152/physrev.00049.2017

[2b] C. E. Müller , K. A. Jacobson , Biochim. Biophys. Acta Biomembr. 2011, 1808, 1290–1308. 10.1016/j.bbamem.2010.12.017 PMC3437328 21185259

[3] J. Kjaergaard , S. Hatfield , G. Jones , A. Ohta , M. Sitkovsky , J. Immunol. 2018, 201, 782–791. 29802128 10.4049/jimmunol.1700850 PMC6052792

[4] A. Ohta , E. Gorelik , S. J. Prasad , F. Ronchese , D. Lukashev , M. K. K. Wong , X. Huang , S. Caldwell , K. Liu , P. Smith , et al , Proc. Natl. Acad. Sci. USA 2006, 103, 13132–13137. 16916931 10.1073/pnas.0605251103 PMC1559765

[5a] A. Young , D. Mittal , K. Stannard , M. Yong , M. W. Teng , B. Allard , J. Stagg , M. J. Smyth , Oncoimmunology 2014, 3, e958952; 25941583 10.4161/21624011.2014.958952 PMC4292728

[5b] S. M. Hatfield , M. Sitkovsky , Curr. Opin. Pharmacol. 2016, 29, 90–96. 27429212 10.1016/j.coph.2016.06.009 PMC4992656

[6] B. B. Fredholm , A. P. IJzerman , K. A. Jacobson , J. Linden , C. E. Müller , Pharmacol. Rev. 2011, 63, 1–34. 21303899 10.1124/pr.110.003285 PMC3061413

[7] A. Hammami , D. Allard , B. Allard , J. Stagg , Semin. Immunol. 2019, 42, 101304. 31604539 10.1016/j.smim.2019.101304

[8] D. Allard , M. Turcotte , J. Stagg , Immunol. Cell Biol. 2017, 95, 333–339. 28174424 10.1038/icb.2017.8

[9] S. N. Schiffmann , G. Fisone , R. Moresco , R. A. Cunha , S. Ferré , Prog. Neurobiol. 2007, 83, 277–292. 17646043 10.1016/j.pneurobio.2007.05.001 PMC2148496

[10] C. Laurent , S. Burnouf , B. Ferry , V. L. Batalha , J. E. Coelho , Y. Baqi , E. Malik , E. Marciniak , E. Mariciniak , S. Parrot , et al , Mol. Psychiatry 2016, 21, 149. 26216297 10.1038/mp.2015.115

[11] I. Villar-Menéndez , S. Porta , S. P. Buira , T. Pereira-Veiga , S. Díaz-Sánchez , J. L. Albasanz , I. Ferrer , M. Martín , M. Barrachina , Neurobiol. Dis. 2014, 69, 206–214. 24892887 10.1016/j.nbd.2014.05.030

[12] F. Calon , M. Dridi , O. Hornykiewicz , P. J. Bédard , A. H. Rajput , T. Di Paolo , Brain 2004, 127, 1075–1084. 15033896 10.1093/brain/awh128

[13] C. Zúñiga-Ramírez , F. Micheli , Future Neurol. 2013, 8, 639–648.

[14] R. A. Hauser , F. Stocchi , O. Rascol , S. B. Huyck , R. Capece , T. W. Ho , P. Sklar , C. Lines , D. Michelson , D. Hewitt , JAMA Neurol. 2015, 72, 1491–1500. 26523919 10.1001/jamaneurol.2015.2268

[15] J. C. Burbiel , W. Ghattas , P. Küppers , M. Köse , S. Lacher , A.-M. Herzner , R. S. Kombu , R. R. Akkinepally , J. Hockemeyer , C. E. Müller , ChemMedChem 2016, 11, 2272–2286. 27531666 10.1002/cmdc.201600255

[16] S. Khanapur , A. van Waarde , K. Ishiwata , K. L. Leenders , R. A. J. O. Dierckx , P. H. Elsinga , Curr. Med. Chem. 2014, 21, 312–328. 24059232 10.2174/09298673113206660265

[17] X. Yang , L. H. Heitman , A. P. IJzerman , D. van der Es , Purinergic Signal. 2021, 17, 85–108. 33313997 10.1007/s11302-020-09753-8 PMC7954947

[18] N. Robertson , A. Jazayeri , J. Errey , A. Baig , E. Hurrell , A. Zhukov , C. J. Langmead , M. Weir , F. H. Marshall , Neuropharmacology 2011, 60, 36–44. 20624408 10.1016/j.neuropharm.2010.07.001

[19] A. S. Doré , N. Robertson , J. C. Errey , I. Ng , K. Hollenstein , B. Tehan , E. Hurrell , K. Bennett , M. Congreve , F. Magnani , et al , Structure 2011, 19, 1283–1293. 21885291 10.1016/j.str.2011.06.014 PMC3732996

[20] H. M. Berman , J. Westbrook , Z. Feng , G. Gilliland , T. N. Bhat , H. Weissig , I. N. Shindyalov , P. E. Bourne , Nucleic Acids Res. 2000, 28, 235–242. 10592235 10.1093/nar/28.1.235 PMC102472

[21] E. Segala , D. Guo , R. K. Y. Cheng , A. Bortolato , F. Deflorian , A. S. Doré , J. C. Errey , L. H. Heitman , A. P. IJzerman , F. H. Marshall , et al , J. Med. Chem. 2016, 59, 6470–6479. 27312113 10.1021/acs.jmedchem.6b00653

[22] M. Congreve , G. A. Brown , A. Borodovsky , M. L. Lamb , Expert Opin. Drug Discovery 2018, 13, 997–1003. 10.1080/17460441.2018.1534825 30336706

[23] A. Borodovsky , C. M. Barbon , Y. Wang , M. Ye , L. Prickett , D. Chandra , J. Shaw , N. Deng , K. Sachsenmeier , J. D. Clarke , et al , J. Immunother. Cancer 2020, 8, e000417. 32727810 10.1136/jitc-2019-000417 PMC7394305

[24] G. Lebon , T. Warne , P. C. Edwards , K. Bennett , C. J. Langmead , A. G. W. Leslie , C. G. Tate , Nature 2011, 474, 521–525. 21593763 10.1038/nature10136 PMC3146096

[25] C. J. Langmead , S. P. Andrews , M. Congreve , J. C. Errey , E. Hurrell , F. H. Marshall , J. S. Mason , C. M. Richardson , N. Robertson , A. Zhukov , M. Weir , J. Med. Chem. 2012, 55, 1904–1909. 22250781 10.1021/jm201455y PMC3308209

[26] J. A. Ballesteros , H. Weinstein in Methods in Neurosciences (Ed.: S. C. Sealfon ), Elsevier, Amsterdam, 1995, pp. 366–428.

[27] K. T. Kimura , H. Asada , A. Inoue , F. M. N. Kadji , D. Im , C. Mori , T. Arakawa , K. Hirata , Y. Nomura , N. Nomura , et al , Nat. Struct. Mol. Biol. 2019, 26, 121–128. 30723326 10.1038/s41594-018-0180-z

[28] S. Yasuda , Y. Kajiwara , Y. Takamuku , N. Suzuki , T. Murata , M. Kinoshita , J. Phys. Chem. 2016, 120, 3833–3843. 10.1021/acs.jpcb.6b01405 27056055

[29] F. Heisig , S. Gollos , S. J. Freudenthal , A. El-Tayeb , J. Iqbal , C. E. Müller , J. Fluoresc. 2014, 24, 213–230. 24052460 10.1007/s10895-013-1289-4

[30] V. Hoguet , M. Lasalle , M. Maingot , G. Dequirez , R. Boulahjar , F. Leroux , C. Piveteau , A. Herledan , A. Biela , J. Dumont , et al , J. Med. Chem. 2021, 64, 1593–1610. 33470812 10.1021/acs.jmedchem.0c01774

[31] C. E. Müller , S. Ferré , Recent Pat. CNS Drug Discovery 2007, 2, 1–21. 18221214 10.2174/157488907779561772

[32] W. Liu , E. Chun , A. A. Thompson , P. Chubukov , F. Xu , V. Katritch , G. W. Han , C. B. Roth , L. H. Heitman , A. P. IJzerman , V. Cherezov , R. C. Stevens , Science 2012, 337, 232–236. 22798613 10.1126/science.1219218 PMC3399762

[33] X. Zhang , R. C. Stevens , F. Xu , Trends Biochem. Sci. 2015, 40, 79–87. 25601764 10.1016/j.tibs.2014.12.005

[34] K. A. Bennett , B. Tehan , G. Lebon , C. G. Tate , M. Weir , F. H. Marshall , C. J. Langmead , Mol. Pharmacol. 2013, 83, 949–958. 23429888 10.1124/mol.112.084509 PMC3629831

[35] K. L. White , M. T. Eddy , Z.-G. Gao , G. W. Han , T. Lian , A. Deary , N. Patel , K. A. Jacobson , V. Katritch , R. C. Stevens , Structure 2018, 26, 259-269.e5. 29395784 10.1016/j.str.2017.12.013 PMC5810373

[36] X. Pang , M. Yang , K. Han , Proteins Struct. Funct. Bioinf. 2013, 81, 1399–1410. 10.1002/prot.24283 23508898

[37] C. E. Müller , J. Maurinsh , R. Sauer , Eur. J. Pharm. Sci. 2000, 10, 259–265. 10838015 10.1016/s0928-0987(00)00064-6

[38] F. Xu , H. Wu , V. Katritch , G. W. Han , K. A. Jacobson , Z.-G. Gao , V. Cherezov , R. C. Stevens , Science 2011, 332, 322–327. 21393508 10.1126/science.1202793 PMC3086811

[39] R. H. J. Olsen , J. F. DiBerto , J. G. English , A. M. Glaudin , B. E. Krumm , S. T. Slocum , T. Che , A. C. Gavin , J. D. McCorvy , B. L. Roth , R.T. Strachan , Nat. Chem. Biol. 2020, 16, 841–849. 32367019 10.1038/s41589-020-0535-8 PMC7648517

[40] V.-P. Jaakola , M. T. Griffith , M. A. Hanson , V. Cherezov , E. Y. T. Chien , J. R. Lane , A. P. IJzerman , R. C. Stevens , Science 2008, 322, 1211–1217. 18832607 10.1126/science.1164772 PMC2586971

[41] J. S. Mason , A. Bortolato , D. R. Weiss , F. Deflorian , B. Tehan , F. H. Marshall , In Silico Pharmacol. 2013, 1, 23.

[42] B. Carpenter , G. Lebon , Front. Pharmacol. 2017, 8, 898. 29311917 10.3389/fphar.2017.00898 PMC5736361

[43] P. Rucktooa , R. K. Y. Cheng , E. Segala , T. Geng , J. C. Errey , G. A. Brown , R. M. Cooke , F. H. Marshall , A. S. Doré , Sci. Rep. 2018, 8, 41. 29311713 10.1038/s41598-017-18570-w PMC5758569

[44] C. Carbajales , J. Azuaje , A. Oliveira , M. I. Loza , J. Brea , M. I. Cadavid , C. F. Masaguer , X. García-Mera , H. Gutiérrez-de-Terán , E. Sotelo , J. Med. Chem. 2017, 60, 3372–3382. 28368607 10.1021/acs.jmedchem.7b00138

[45] F. F. Sherbiny , A. C. Schiedel , A. Maass , C. E. Müller , J. Comput.-Aided Mol. Des. 2009, 23, 807–828. 19757091 10.1007/s10822-009-9299-7

[46] X. Wang , W. Jespers , R. Prieto-Díaz , M. Majellaro , A. P. IJzerman , G. J. P. van Westen , E. Sotelo , L. H. Heitman , H. Gutiérrez-de-Terán , Sci. Rep. 2021, 11, 14171. 34238993 10.1038/s41598-021-93419-x PMC8266863

[47] P. G. Baraldi , B. Cacciari , R. Romagnoli , K.-N. Klotz , G. Spalluto , K. Varani , S. Gessi , S. Merighi , P. A. Borea , Drug Dev. Res. 2001, 53, 225–235.

