# Supplementary material for: Single Stabilizing Point Mutation Enables High‐Resolution Co‐Crystal Structures of the Adenosine A2A Receptor with Preladenant Conjugates
Source: Angew Chem Int Ed Engl. 2022 Mar 24;61(22):e202115545. doi: 10.1002/anie.202115545 (PMC9310709; doi:10.1002/anie.202115545)

Angewandte Chemie
Eine Zeitschrift der Gesellschaft Deutscher Chemiker

Supporting Information

Single Stabilizing Point Mutation Enables High-Resolution Co-Crystal Structures of the Adenosine A_{2A} Receptor with Preladenant Conjugates

*T. Claff, T. A. Klapschinski, U. K. Tiruttani Subhramanyam, V. J. Vaaßen, J. G. Schlegel, C. Vielmuth, J. H. Voß, J. Labahn, C. E. Müller**

Author Contributions

T.C. Investigation:Lead; Methodology:Lead; Validation:Lead; Writing – original draft:Lead
T.K. Investigation:Lead; Writing – review & editing:Supporting
U.S. Formal analysis:Lead; Investigation:Equal; Validation:Equal; Writing – review & editing:Supporting
J.S. Investigation:Supporting; Methodology:Supporting; Writing – review & editing:Supporting
C.V. Investigation:Supporting; Writing – review & editing:Supporting
J.V. Investigation:Equal; Methodology:Equal; Writing – review & editing:Supporting
J.L. Data curation:Equal; Formal analysis:Equal; Supervision:Equal; Writing – original draft:Supporting; Writing – review & editing:Supporting
C.M. Conceptualization:Lead; Supervision:Lead; Writing – original draft:Equal

Table of Contents

1. Experimental Procedures 3

1.1 Synthesis of Preladenant derivatives 3

1.2 Expression of A_{2A}AR constructs in Sf9 insect cells 7

1.3 Protein purification for crystallization experiments 7

1.4 Protein purification for stability screening 7

1.5 Thermal shift assay 8

1.6 Crystallization 8

1.7 Data collection and structure determination 8

1.8 Radioligand binding assays 8

1.9 TRUPATH assay 9

1.10 Synthesis of carboxy-functionalized Preladenant analog 2 10

1.10.1 Synthesis of the side-chain (a) 10

1.10.2 Synthesis of the tricyclic Preladenant core (19) and attachment of the side chain yielding the functionalized Preladenant derivative 2 (b) 11

1.10.3 Synthesis of amino-substituted PEG derivatives 3a-3f 13

2. Supplementary Figures 15

3. Supplementary Tables 18

SUPPORTING INFORMATION

1. Experimental Procedures

1.1 Synthesis of Preladenant derivatives

General

Chemicals were purchased from Merck (Darmstadt, Germany), ABCR (Karlsruhe, Germany), or TCI (Eschborn, Germany); dried solvents were purchased from Acros Organics (Fisher Scientific GmbH, Schwerte, Germany). Solvents were of HPLC quality. Thin-layer chromatography (TLC) was performed on silica gel plates F_{254} (0,25 mm) or on reverse phase silica gel plates 60 RP-18 F_{254} (Merck). Compounds were detected under UV light (254 nm, 366 nm) or by spraying the plates with a basic $KMnO_4$ solution (1.5 g of $KMnO_4$, 10 g of K_2CO_3, and 1.25 ml of 10% aq. NaOH solution in 200 ml of water), or a ninhydrin solution (1.5 g of ninhydrin dissolved in 100 mL of butanol containing 3.0 ml of acetic acid). Reaction controls were performed by measuring the molecular masses of the products on an Advion Expression L mass spectrometer with a TLC interface, following atomic pressure chemical ionization (APCI) or electrospray ionization (ESI). Column chromatography was performed using glass columns filled with silica gel 60 (35-70 µm), or using the automated flash chromatography system Combiflash Rf 200 (Teledyne ISCO, Nebraska, USA). The final compounds were purified by reversed phase HPLC (Knauer, Berlin, Germany), on a C18ec column, 250 x 20 mm, Nucleodur 100-5. Analytical LCMS spectra were obtained on an API2000 mass spectrometer with an ESI source (ABSciex, Darmstadt, Germany) coupled with an Agilent HPLC HP1100 system (column: EC50/2 Nucleodur C18 Gravity 3 µm, Macherey-Nagel, Düren, Germany) at 25 °C. The following eluents were used: water containing 2 mM ammonium acetate (A) and methanol containing 2 mM ammonium acetate (B). A gradient was used starting from 90% A, reaching 100% B within 10 min. The column was flushed for additional 10 min with B. The flow rate was 0.3 ml/min. The samples were dissolved in B or in acetonitrile at a concentration of 1 mg/ml, and 8 µl of the solution were injected. The total ion current (TIC) was measured for the relevant masses, routinely from 150-800 m/z. UV absorption was measured from 190-900 nm using a diode array detector (DAD), and purity was determined at 220-400 nm.
High resolution mass spectra were obtained by HPLC-QToF-MS spectroscopy on a Bruker micrOTOF-Q mass spectrometer connected to a Dionex Ultimate 3000 HPLC system (column: EC50/2 Nucleodur C18 Gravity 3 µm, Macherey-Nagel, Düren, Germany). Conditions were the same as described above.
UV spectra were determined from 300 to 800 nm on a Varian/Cary 50 Bio instrument (Agilent Technologies, USA). Fluorescence spectra were recorded on a Flx-Monaco spektrofluorometer (Monaco, Monaco) using a xenon lamp. The band width was 5 nm for excitation and emission, and the emission was recorded from 300 to 800 nm. The excitation wavelenth was selected according to the absorption maxima. Stock solutions were prepared in DMSO (10 mM or 0.1 mM), and 1 µl of stock solution was added to a cuvette containing 990 µl of solvent (H_2O, CH_2Cl_2 or MeOH) and 9 µl of DMSO. The final DMSO concentration ways 1%, and the final compound concentration was 10 µM for UV spectra determination, and 0.1 µM for fluorescence spectra.
^{1}H and ^{13}C NMR spectra were measured in $CDCl_3$ or DMSO-d_6 on a Bruker Ascend 600 MHz spectrometer at 600.18 MHz (^{1}H) and 150.93 MHz (^{13}C). Chemical shifts (δ) are given in ppm, and were related to the solvent signals ($CDCl_3$, 7.26 ppm for ^{1}H NMR spectra, 77.16 ppm for ^{13}C NMR spectra; DMSO-d_6, 2.50 ppm for ^{1}H NMR spectra, 39.52 ppm for ^{13}C NMR spectra). Multiplicities are designated as follows: s, singlet; d, doublet; q, quartet; p, pentet; sext, sextet; m, multiplet; b, broad. Coupling constants (J) are in Hertz (Hz). Melting points were determined on a Büchi 545 melting point apparatus and are uncorrected. The syntheses of the functionalized Preladenant derivatives **1** and **2** and of the amino-substituted PEG linkers **3a**-**3f** are described in **Scheme S1**.

***tert*-Butyl 1-(4-(4-(2-(5-amino-2-(furan-2-yl)-7*H*-pyrazolo[4,3-*e*][1,2,4]triazolo[1,5-*c*]pyrimidin-7-yl)ethyl)piperazin-1-yl)phenoxy)-2-oxo-6,9,12,15-tetraoxa-3-azaheptadecan-17-oate (4, PSB-2113)**

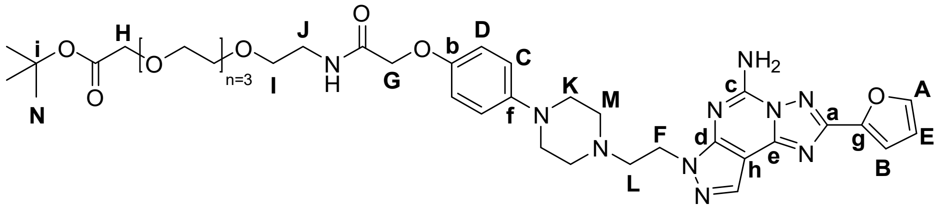

Compound **2** (20 mg, 0.04 mmol, 1.0 eq.) is dissolved in dichloromethane (500 µl) and HATU (17.9 mg, 0.07 mmol, 1.2 eq.) is added. In a separate vial, compound **3a** (13.5 mg, 0.044 mmol, 1.1 eq.) is dissolved in dichloromethane (500 µl) and diisopropylethylamine (10.3 mg, 0.08 mmol, 2.0 eq.) is added. After 5 min of pre-activation of the carboxylic acid **2**, the solution of the amine **3a** is added, and the mixture is stirred for 24 h at room temperature (RT). Then, the solvent is removed *in vacuo*, and the product is purified by HPLC, applying a gradient within 15 min from 70% aq. methanol to 100% methanol, yielding 25.1mg (0.032 mmol, 79%) of **4**.
^{1}H NMR (600 MHz, DMSO-d_6) δ [ppm]: 8.16 (s, 1H, NCH), 8.05 (bs, 2H, NH_2), 7.95 (t, $^3J_{H,H}$ = 5.73 Hz, 1H, NH), 7.94–7.93 (m, 1H, A), 7.22 (dd, $^3J_{H,H}$ = 3.32 Hz, $^3J_{H,H}$ = 0.85 Hz 1H, B), 6.88–6.80 (m, 4H, C, D), 6.73 (dd, $^3J_{H,H}$ = 1.77 Hz, $^3J_{H,H}$ = 3.41 Hz, 1H, E), 4.42 (t, $^3J_{H,H}$ = 6.79 Hz 2H, F), 4.36 (s, 2H, G), 3.96 (s, 2H, H), 3.56–3.54 (m, 2H, CH_2), 3.52–3.48 (m, 10H, 5 x CH_2), 3.44 (t, $^3J_{H,H}$ = 5.96 Hz, I), 3.30–3.25 (m, 2H, J), 2.98–2.93 (m, 4H, K), 2.83 (t, $^3J_{H,H}$ = 6.81 Hz, 2H, L), 2.61–2.58 (m, 4H, M), 1.40 (s, 9H, N). **^{13}C NMR** (126 MHz, DMSO-d_6) δ [ppm]: 169.3 (C_q, C_{ester}), 168.0 (C_q, C_{amide}), 155.4 (C_q, a), 151.2 (C_q, b), 148.7 (C_q, c), 148.6 (C_q, d), 146.2 (C_q, e), 146.0 (C_q, f), 145.5 (C_q, g), 145.1 (CH, A), 131.4 (CH, NCH), 117.1 (2 x CH, C, D), 115.3 (2 x CH, C, D), 112.3 (CH, B), 112.1 (CH, B), 95.7 (C_q, h), 80.6 (C_q, i), 69.9 (CH_2), 69.8 (CH_2), 69.7 (2 x CH_2), 69.7 (CH_2), 69.5 (CH_2), 68.8 (CH_2), 68.1 (CH_2), 67.6 (CH_2), 56.5 (CH_2,

L), 52.6 (2 x CH_2, M), 49.4 (2 x CH_2, K), 44.4 (CH_2, F), 38.2 (CH_2, J), 27.7 (3 x CH_3, N). **Mp**. 115.9–117.6 °C. **HRMS** (ESI-QTOF) calculated for $C_{38}H_{52}N_{10}O_9$ $[M+H]^+$: 793.3991; found: 793.3850. **Purity**: 99%.

***tert*-Butyl 1-(4-(4-(2-(5-amino-2-(furan-2-yl)-7*H*-pyrazolo[4,3-e][1,2,4]triazolo[1,5-*c*]pyrimidin-7-yl)ethyl)piperazin-1-yl)phenoxy)-2-oxo-6,9,12,15,18-pentaoxa-3-azaicosan-20-oate (5)**

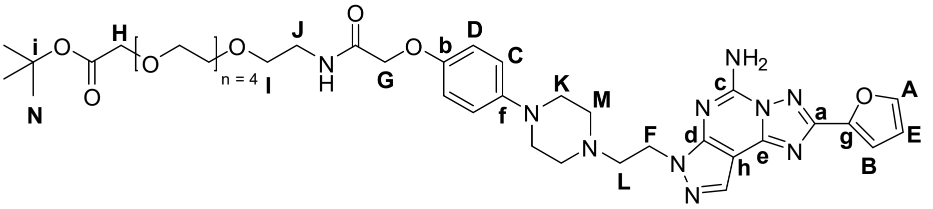

The reaction is performed as described for the synthesis of compound **4** from 2.8 mg (5.5 µmol, 1.0 eq.) of **2** and HATU (2.5 mg, 6.6 µmol, 1.2 eq.) dissolved in 500 µl of dichloromethane, and 2.1 mg (6.1 µmol, 1.1 eq.) of **3b**, in the presence of 2.0 µl (11.0 µmol, 2.0 eq.) of diisopropylethylamine. The product was purified by HPLC as described for compound **4**. Yield: 3.6 mg (4.3 µmol, 78%) of product **5**.

^{1}H NMR (600 MHz, DMSO-d_6) *δ* [ppm]: 8.16 (s, 1H, NCH), 8.05 (bs, 2H, NH_2), 7.95 (t, $^3J_{H,H}$ = 5.75 Hz, 1H, NH), 7.94–7.93 (m, 1H, A), 7.22 (d, $^3J_{H,H}$ = 3.32 Hz, 1H, B), 6.87–6.84 (m, 2H, C), 6.84–6.81 (m, 2H, D), 6.73 (dd, $^3J_{H,H}$ = 1.71 Hz, $^3J_{H,H}$ = 3.45 Hz, 1H, E), 4.42 (t, $^3J_{H,H}$ = 6.83 Hz, 2H, F), 4.36 (s, 2H, G), 3.97 (s, 2H, H), 3.58–3.47 (m, 16H, PEG), 3.44 (t, $^3J_{H,H}$ = 5.96Hz, I), 3.30–3.26 (m, 2H, J), 2.99–2.92 (m, 4H, K), 2.83 (t, $^3J_{H,H}$ = 6.85 Hz, 2H, L), 2.62–2.57 (m, 4H, M), 1.41 (s, 9H, N). **^{13}C NMR** (151 MHz, DMSO-d_6) *δ* [ppm]: 169.3 (C_q, C_{Ester}), 168.0 (C_q, C_{amide}), 155.4 (C_q, a), 151.2 (C_q, b), 148.7 (C_q, c), 148.6 (C_q, d), 146.2 (C_q, e), 146.0 (C_q, f), 145.5 (C_q, g), 145.1 (CH, A), 131.4 (CH, NCH), 117.0 (2 x CH, C, D), 115.3 (2 x CH, C, D), 112.2 (CH, B), 112.1 (CH, E), 95.6 (C_q, h), 80.6 (C_q, i), 69.8 (CH_2, PEG), 69.8 (CH_2, PEG), 69.8 (CH_2, PEG), 69.7 (CH_2, PEG), 69.7 (CH_2, PEG), 69.7 (CH_2, PEG), 69.7 (CH_2, PEG), 69.5 (CH_2, PEG), 68.8 (CH_2, I), 68.1 (CH_2, H), 67.6 (CH_2, G), 56.5 (CH_2, L), 52.6 (2 x CH_2, M), 49.4 (2 x CH_2, K), 44.4 (CH_2, F), 38.2 (CH_2, J), 27.7 (3 x CH_3, N). **Mp.** 295 – 300 °C (decomposition). **LCMS**: positive [*m*/*z*] = 837.5 ($[M+H]^+$). **HRMS** (ESI-QTOF) calculated for $C_{40}H_{56}N_{10}O_{10}$ $[M+Na]^+$: 859.4073; found: 859.4074. **Purity**: 96%.

***tert*-Butyl 1-(4-(4-(2-(5-amino-2-(furan-2-yl)-7*H*-pyrazolo[4,3-e][1,2,4]triazolo[1,5-*c*]pyrimidin-7-yl)ethyl)piperazin-1-yl)phenoxy)-2-oxo-6,9,12,15,18,21,24,27-octaoxa-3-azanonacosan-29-oate (6)**

The reaction is performed as described for the synthesis of compound **4** from 20 mg (0.04 mmol, 1.0 eq.) of **2** and HATU (17.9 mg, 0.07 mmol, 1.2 eq.) dissolved in 500 µl of dichloromethane, and 21.3 mg (0.044 mol, 1.1 eq.) of **3c**, in the presence of 10.3 mg (0.08 mmol, 2.0 eq.) of diisopropylethylamine. The product is purified by HPLC as described for compound **4**. Yield: 37.9 mg (0.039 mmol, 98%) of product **6**.

^{1}H NMR (600 MHz, DMSO-d_6) *δ* [ppm]: 8.16 (s, 1H, NCH), 8.05 (bs, 2H, NH_2), 7.95 (t, $^3J_{H,H}$ = 5.75 Hz, 1H, NH), 7.94–7.93 (m, 1H, A), 7.22 (d, $^3J_{H,H}$ = 3.30 Hz, 1H, B), 6.87–6.81 (m, 4H, C+D), 6.73 (dd, $^3J_{H,H}$ = 1.74 Hz, $^3J_{H,H}$ = 3.39 Hz, 1H, E), 4.42 (t, $^3J_{H,H}$ = 6.81 Hz, 2H, F), 4.36 (s, 2H, G), 3.97 (s, 2H, H), 3.57–3.54 (m, 2H, CH_2), 3.53–3.51 (m, 2H, CH_2), 3.51–3.47 (m, 24H, 12 x CH_2), 3.44 (t, $^3J_{H,H}$ = 5.96 Hz, I), 3.28 (q, $^3J_{H,H}$ = 5.92 Hz, 2H, J), 2.99–2.93 (m, 4H, K), 2.83 (t, $^3J_{H,H}$ = 6.80 Hz, 2H, L), 2.62–2.57 (m, 4H, M), 1.41 (s, 9H, N). **^{13}C NMR** (151 MHz, DMSO-d_6) *δ* [ppm]: 169.3 (C_q, C_{ester}), 168.0 (C_q, C_{amide}), 155.3 (C_q, a), 151.2 (C_q, b), 148.7 (C_q, c), 148.6 (C_q, d), 146.2 (C_q, e), 146.0 (C_q, f), 145.5 (C_q, g), 145.1 (CH, A), 131.4 (CH, NCH), 117.0 (2 x CH, C, D), 115.3 (2 x CH, C, D), 112.2 (CH, B), 112.1 (CH, E), 95.6 (C_q, h), 80.6 (C_q, i), 69.8 (CH_2, PEG), 69.8 (9 x CH_2, PEG), 69.7 (2 x CH_2, PEG), 69.7 (CH_2, PEG), 69.5 (CH_2, PEG), 68.8 (CH_2, I), 68.1 (CH_2, H), 67.6 (CH_2, G), 56.5 (CH_2, L), 52.6 (2 x CH_2, M), 49.3 (2 x CH_2, K), 44.4 (CH_2, F), 38.2 (CH_2, J), 27.7 (3 x CH_3, N). Mp: could not be determined due to hygroscopic nature of the compound. **HRMS** (ESI-QTOF) calculated for $C_{46}H_{69}N_{10}O_{13}$ $[M+H]^+$: 969.5040; found: 969.5027. **Purity**: 98%.

***tert*-Butyl 1-(4-(4-(2-(5-amino-2-(furan-2-yl)-7*H*-pyrazolo[4,3-*e*][1,2,4]triazolo[1,5-*c*]pyrimidin-7-yl)ethyl)piperazin-1-yl)phenoxy)-2-oxo-6,9,12,15,18,21,24,27,30,33,36,39-dodecaoxa-3-azahentetracontan-41-oate (7)**

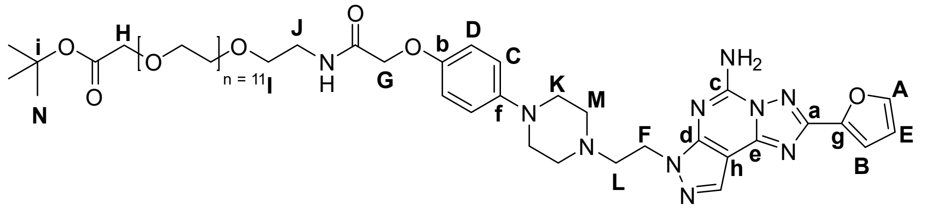

The reaction is performed as described for the synthesis of compound **4** from 20 mg (0.04 mmol, 1.0 eq.) of **2** and HATU (17.9 mg, 0.07 mmol, 1.2 eq.) dissolved in 500 µl of dichloromethane, and 29.0 mg (0.044 mol, 1.1 eq.) of **3d**, in the presence of 10.3 mg (0.08 mmol, 2.0 eq.) of diisopropylethylamine. The product is purified by HPLC as described for compound **4**. Yield: 36.6 mg (0.032 mmol, 80%) of product **7**.

^{1}H NMR (600 MHz, DMSO-*d*$_6$) *δ* [ppm]: 8.16 (s, 1H, NCH), 8.05 (bs, 2H, NH_2), 7.95 (t, $^3J_{H,H}$ = 5.73 Hz, 1H, NH), 7.94–7.93 (m, 1H, A), 7.22 (d, $^3J_{H,H}$ = 3.35 Hz, 1H, B), 6.87–6.81 (m, 4H, C+D), 6.73 (dd, $^3J_{H,H}$ = 1.73 Hz, $^3J_{H,H}$ = 3.51 Hz, 1H, E), 4.42 (t, $^3J_{H,H}$ = 6.78 Hz, 2H, F), 4.36 (s, 2H, G), 3.97 (s, 2H, H), 3.58–3.54 (m, 2H, CH_2), 3.53–3.51 (m, 2H, CH_2), 3.51–3.47 (m, 40H, 20 x CH_2), 3.44 (t, $^3J_{H,H}$ = 5.96 Hz, I), 3.28 (q, $^3J_{H,H}$ = 5.87 Hz, 2H, J), 2.99–2.93 (m, 4H, K), 2.83 (t, $^3J_{H,H}$ = 6.80 Hz, 2H, L), 2.61–2.57 (m, 4H, M), 1.41 (s, 9H, N). **^{13}C NMR** (151 MHz, DMSO-*d*$_6$) *δ* [ppm]: 169.4 (C_q, C_{Ester}), 168.0 (C_q, C_{amide}), 155.4 (C_q, a), 151.2 (C_q, b), 148.7 (C_q, c), 148.6 (C_q, d), 146.2 (C_q, e), 146.0 (C_q, f), 145.5 (C_q, g), 145.1 (CH, A), 131.4 (CH, NCH), 117.0 (2 x CH, C, D), 115.3 (2 x CH, C, D), 112.3 (CH, B), 112.1 (CH, E), 95.7 (C_q, h), 80.6 (C_q, i), 69.9 (CH_2, PEG), 69.8 (17 x CH_2, PEG), 69.7 (CH_2, PEG), 69.7 (CH_2, PEG), 69.7 (CH_2, PEG), 69.5 (CH_2, PEG), 68.8 (CH_2, I), 68.1 (CH_2, H), 67.6 (CH_2, G), 56.5 (CH_2, L), 52.6 (2 x CH_2, M), 49.4 (2 x CH_2, K), 44.4 (CH_2, F), 38.2 (CH_2, J), 27.7 (3x CH_3, N). Mp could not be determined due to the hygroscopic nature of the compound. **HRMS** (ESI-QTOF) calculated for $C_{54}H_{84}N_{10}O_{17}$ $[M+H]^+$: 1145.6089; found: 1145.6064. **Purity**: 98%.

***tert*-Butyl 1-(4-(4-(2-(5-amino-2-(furan-2-yl)-7*H*-pyrazolo[4,3-*e*][1,2,4]triazolo[1,5-*c*]pyrimidin-7-yl)ethyl)piperazin-1-yl)phenoxy)-2-oxo-6,9,12,15,18,21,24,27,30,33,36,39,42,45,48,51-hexadecaoxa-3-azatripentacontan-53-oate (8)**

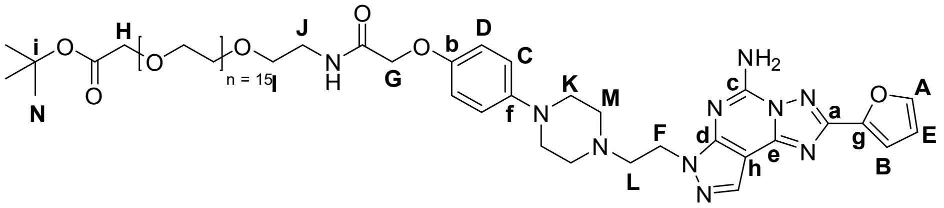

The reaction is performed as described for the synthesis of compound **4** from 20 mg (0.04 mmol, 1.0 eq.) of **2** and HATU (17.9 mg, 0.07 mmol, 1.2 eq.) dissolved in 500 µl of dichloromethane, and 36.8 mg (0.044 mol, 1.1 eq.) of **3e**, in the presence of 10.3 mg (0.08 mmol, 2.0 eq.) of diisopropylethylamine. The product is purified by HPLC as described for compound **4**. Yield: 46.4 mg (0.035 mmol, 88%) of product **8**.

^{1}H NMR (600 MHz, DMSO-*d*$_6$) *δ* [ppm]: 8.16 (s, 1H, NCH), 8.05 (bs, 2H, NH_2), 7.95 (t, $^3J_{H,H}$ = 5.71 Hz, 1H, NH), 7.94–7.93 (m, 1H, A), 7.22 (d, $^3J_{H,H}$ = 3.36 Hz, 1H, B), 6.87–6.81 (m, 4H, C+D), 6.73 (dd, $^3J_{H,H}$ = 1.75 Hz, $^3J_{H,H}$ = 3.40 Hz, 1H, E), 4.42 (t, $^3J_{H,H}$ = 6.81 Hz, 2H, F), 4.36 (s, 2H, G), 3.97 (s, 2H, H), 3.58–3.55 (m, 2H, CH_2), 3.54–3.51 (m, 2H, CH_2), 3.52–3.48 (m, 56H, 28 x CH_2), 3.44 (t, $^3J_{H,H}$ = 5.96 Hz, I), 3.28 (q, $^3J_{H,H}$ = 5.86 Hz, 2H, J), 2.99–2.93 (m, 4H, K), 2.83 (t, $^3J_{H,H}$ = 6.81 Hz, 2H, L), 2.63–2.57 (m, 4H, M), 1.41 (s, 9H, N). **^{13}C NMR** (151 MHz, DMSO-*d*$_6$) *δ* [ppm]: 169.3 (C_q, C_{Ester}), 168.0 (C_q, C_{Amid}), 155.4 (C_q, a), 151.2 (C_q, b), 148.7 (C_q, c), 148.6 (C_q, d), 146.2 (C_q, e), 146.0 (C_q, f), 145.5 (C_q, g), 145.1 (CH, A), 131.4 (CH, NCH), 117.0 (2 x CH, C, D), 115.3 (2 x CH, C, D), 112.2 (CH, B), 112.1 (CH, E), 95.6 (C_q, h), 80.6 (C_q, i), 69.8 (CH_2, PEG), 69.8 (25 x CH_2, PEG), 69.7 (CH_2, PEG), 69.7 (CH_2, PEG), 69.7 (CH_2, PEG), 69.5 (CH_2, PEG), 68.8 (CH_2, I), 68.1 (CH_2, H), 67.6 (CH_2, G), 56.5 (CH_2, L), 52.6 (2x CH_2, M), 49.3 (2x CH_2, K), 44.4 (CH_2, F), 38.2 (CH_2, J), 27.7 (3x CH_3, N). Mp could not be determined due to the hygroscopic nature of the compound. **HRMS** (ESI-QTOF) calculated for $C_{62}H_{100}N_{10}O_{21}$ $[M+H]^+$: 1321.7086; found: 1321.7137. **Purity**: 98%.

***tert*-Butyl 1-(4-(4-(2-(5-amino-2-(furan-2-yl)-7*H*-pyrazolo[4,3-e][1,2,4]triazolo[1,5-*c*]pyrimidin-7-yl)ethyl)piperazin-1-yl)phenoxy)-2-oxo-6,9,12,15,18,21,24,27,30,33,36,39,42,45,48,51,54,57,60,63-icosaoxa-3-azapentahexacontan-65-oate (9)**

The reaction is performed as described for the synthesis of compound **4** from 20 mg (0.04 mmol, 1.0 eq.) of **2** and HATU (17.9 mg, 0.07 mmol, 1.2 eq.) dissolved in 500 µl of dichloromethane, and 44.5 mg (0.044 mol, 1.1 eq.) of **3f**, in the presence of 10.3 mg (0.08 mmol, 2.0 eq.) of diisopropylethylamine. The product is purified by HPLC as described for compound **4**. Yield: 52.7 mg (0.035 mmol, 88%) of product **9**.

^{1}H NMR (600 MHz, DMSO-*d*$_6$) *δ* [ppm]: 8.16 (s, 1H, NCH), 8.05 (bs, 2H, NH_2), 7.95 (t, $^3J_{H,H}$ = 5.82 Hz, 1H, NH), 7.93 (dd, $^3J_{H,H}$ = 1.78 Hz, $^3J_{H,H}$ = 0.77 Hz, 1H, A), 7.22 (dd, $^3J_{H,H}$ = 3.43 Hz, $^3J_{H,H}$ = 0.77 Hz, 1H, B), 6.87–6.81 (m, 4H, C+D), 6.73 (dd, $^3J_{H,H}$ = 1.76 Hz, $^3J_{H,H}$ = 3.41 Hz, 1H, E), 4.42 (t, $^3J_{H,H}$ = 6.81 Hz, 2H, F), 4.36 (s, 2H, G), 3.97 (s, 2H, H), 3.58–3.55 (m, 2H, CH_2), 3.54–3.51 (m, 2H, CH_2), 3.51–3.48 (m, 72H, 28 x CH_2), 3.44 (t, $^3J_{H,H}$ = 5.96 Hz, I), 3.28 (q, $^3J_{H,H}$ = 5.88 Hz, 2H, J), 2.98–2.94 (m, 4H, K), 2.83 (t, $^3J_{H,H}$ = 6.79 Hz, 2H, L), 2.61–2.57 (m, 4H, M), 1.42 (s, 9H, N). **^{13}C NMR** (151 MHz, DMSO-*d*$_6$) *δ* [ppm]: 169.3 (C_q, C_{Ester}), 168.0 (C_q, C_{Amid}), 155.4 (C_q, a), 151.2 (C_q, b), 148.7 (C_q, c), 148.6 (C_q, d), 146.2 (C_q, e), 146.0 (C_q, f), 145.5 (C_q, g), 145.1 (CH, A), 131.4 (CH, NCH), 117.0 (2 x CH, C, D), 115.3 (2 x CH, C, D), 112.2 (CH, B), 112.1 (CH, E), 95.6 (C_q, h), 80.6 (C_q, i), 69.8 (CH_2, PEG), 69.8 (33 x CH_2, PEG), 69.7 (CH_2, PEG), 69.7 (CH_2, PEG), 69.7 (CH_2, PEG), 69.7 (CH_2, PEG), 69.5 (CH_2, PEG), 68.8 (CH_2, I), 68.1 (CH_2, H), 67.6 (CH_2, G), 56.5 (CH_2, L), 52.6 (2 x CH_2,

M), 49.3 (2 x CH_2, K), 44.4 (CH_2, F), 38.2 (CH_2, J), 27.7 (3x CH_3, N). Mp could not be determined due to the hygroscopic nature of the compound. **HRMS** (ESI-QTOF) calculated fro $C_{70}H_{116}N_{10}O_{25}$ $[M+H]^+$: 1497.8139; found: 1497.8186. **Purity**: 96%.

1-(4-(4-(2-(5-Amino-2-(furan-2-yl)-7*H*-pyrazolo[4,3-*e*][1,2,4]triazolo[1,5-*c*]pyrimidin-7-yl)ethyl)piperazin-1-yl)phenoxy)-2-oxo-6,9,12,15-tetraoxa-3-azaheptadecan-17-carboxylic acid (10)

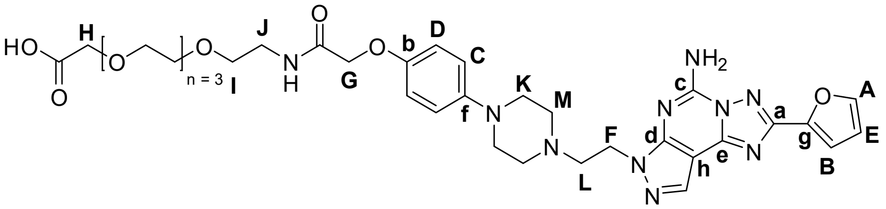

Compound **4** (8.7 mg, 0.01 mmol, 1.0 eq.) is dissolved in a mixture of trifluoroacetic acid/dichloromethane (6:4), and 25 µl of TIPS are added. The mixture is stirred for 2h at RT. Then, the solvent is removed *in vacuo*, 200 µl of acetonitrile and a few drops of trifluoroacetic acid are added until fully dissolved, and the product is purified by HPLC, applying a gradient from 50% aq. acetonitrile reaching 100% acetonitrile within 15 min, then eluting for 5 more min yielding 6.9 mg (0.0093 mmol, 93%) of carboxylic acid derivative **10**.

^{1}H NMR (600 MHz, DMSO-*d*$_6$) δ [ppm]: 8.64 (t, $^3J_{H,H}$ = 5.85 Hz, NH), 8.16 (s, 1H, NCH), 8.08 (bs, 2H, NH_2), 7.95–7.91 (m, 1H, A), 7.22 (d, $^3J_{H,H}$ = 3.37 Hz, 1H, B), 6.84–6.82 (m, 4H, C, D), 6.73 (dd, $^3J_{H,H}$ = 1.77 Hz, $^3J_{H,H}$ = 3.43 Hz, 1H, E), 4.42 (s, 2H, G), 4.42 (t, $^3J_{H,H}$ = 6.72 Hz 2H, F), 3.55 (s, 2H, H), 3.53–3.49 (m, 12H, 6 x CH_2), 3.47 (t, $^3J_{H,H}$ = 5.61 Hz, I), 3.31–3.26 (m, 2H, J), 2.98–2.93 (m, 4H, K), 2.83 (t, $^3J_{H,H}$ = 6.81 Hz, 2H, L), 2.62–2.57 (m, 4H, M). **^{13}C NMR** (151 MHz, DMSO-*d*$_6$) δ [ppm]: 171.8 (C_q, C_{acid}), 168.0 C_q, C_{amide}), 155.3 (C_q, a), 151.2 (C_q, b), 148.7 (C_q, c), 148.6 (C_q, d), 146.2 (C_q, e), 145.9 (C_q, f), 145.5 (C_q, g), 145.1 (CH, A), 131.4 (CH, NCH), 117.0 (2 x CH, C, D), 115.3 (2 x CH, C, D), 112.2 (CH, B), 112.1 (CH, E), 95.6 (C_q, h), 69.7 (2 x CH_2), 69.7 (CH_2), 69.6 (CH_2), 69.5 (2 x CH_2), 68.8 (CH_2), 68.4 (CH_2), 67.5 (CH_2, G), 56.5 (CH_2, L), 52.6 (2 x CH_2, M), 49.3 (2 x CH_2, K), 44.4 (CH_2, F), 38.2 (CH_2, J). **Mp**: 183.2–183.3 °C**.** **HRMS** (ESI-QTOF) calculated for $C_{34}H_{45}N_{10}O_9$ $[M+H]^+$: 737.3365; found: 737.3360. **Purity**: 99%.

14-(2-(4-(4-(2-(5-Amino-2-(furan-2-yl)-7*H*-pyrazolo[4,3-*e*][1,2,4]triazolo[1,5-*c*]pyrimidin-7-yl)ethyl)piperazin-1-yl)phenoxy)acetamido)-*N*-(5-(5,5-difluoro-1,3,7,9-tetramethyl-5*H*-4*l*4,5*l*4-dipyrrolo[1,2-*c*:2',1'-*f*][1,3,2]diazaborinin-10-yl)pentyl)-3,6,9,12-tetraoxatetradecanamide (11, PSB-2115)

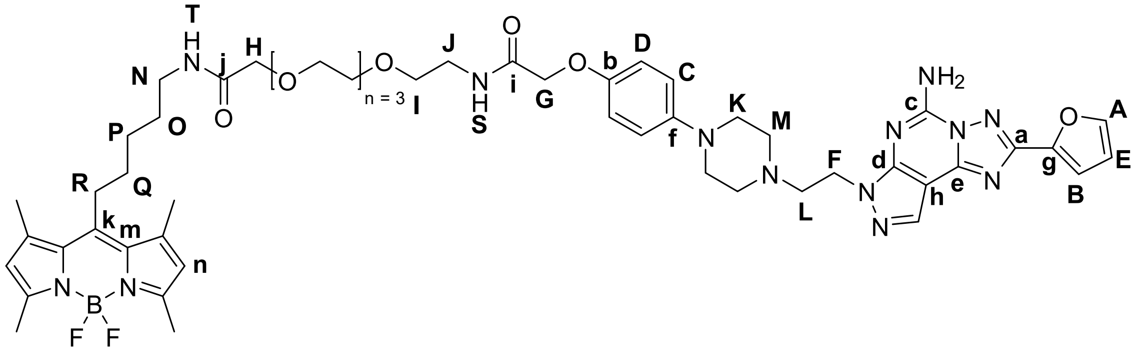

Carboxylic acid derivative **10** (7.4 mg, 0.01 mmol, 1.0 eq.) is dissolved in 0.2 ml of dichloromethane, and HATU (4.6 mg, 0.012 mmol, 1.2 eq.) is added. In a separate vial, the aminopentyl-substituted BODIPY derivative (8-(5-aminopentyl)-4,4-difluoro-1,3,5,7-tetramethyl-4-bora-3a,4a-diaza-*s*-indacene, 5 mg, 0.015 mmol, 1.1 eq.), synthesized as previously described[1], was dissolved in 0.2 ml of dichloromethane, and diisopropylethylamine (2.6 mg, 0.02 mmol, 2.0 eq.) was added. After 5 min of pre-activation of carboxylic acid **10**, the solution of the amino-substituted BODIPY derivative is added, and the mixture is stirred at RT for 12h. The product is purified by

^{1}H NMR (600 MHz, DMSO-*d*$_6$) δ [ppm]: 8.16 (s, 1H, NCH), 8.05 (bs, 2H, NH_2), 7.94 (t, $^3J_{H,H}$ = 4.59 Hz, 1H, S), 7.95–7.91 (m, 1H, A), 7.64 (t, $^3J_{H,H}$ = 5.76 Hz, 1H, T), 7.22 (d, $^3J_{H,H}$ = 3.31 Hz, 1H, B), 6.87–6.80 (m, 4H, C, D), 6.72 (dd, $^3J_{H,H}$ = 1.80 Hz, $^3J_{H,H}$ = 3.43 Hz, 1H, E), 6.21 (s, 2H, n), 4.41 (t, $^3J_{H,H}$ = 6.72 Hz, 2H, F), 4.35 (s, 2H, G), 3.85 (s, 2H, H), 3.57–3.45 (m, 12H, 6 x CH_2), 3.42 (t, $^3J_{H,H}$ = 6.01 Hz, I), 3.27 (dt, $^3J_{H,H}$ = 6.01 Hz, $^3J_{H,H}$ = 6.01 Hz, 2H, J), 3.12 (dt, $^3J_{H,H}$ = 6.58 Hz, $^3J_{H,H}$ = 6.58 Hz, 2H, N), 2.98–2.93 (m, 4H, K), 2.94–2.89 (m, 2H, R), 2.86–2.80 (m, 2H, L), 2.62–2.56 (m, 4H, M), 2.39 (s, 6H, 2 x CH_3), 2.39 (s, 6H, 2 x CH_3), 1.59–1.52 (m, 2H, Q), 1.52–1.47 (m, 2H, O) 1.47–1.41 (m, 2H, P). **^{13}C NMR** (151 MHz, DMSO-*d*$_6$) δ [ppm]: 169.1 (C_q, j), 168.0 (C_q, i), 155.4 (C_q, a), 153.0 (2 x C_q, C_{BODIPY}), 151.2 (C_q, b), 148.7 (C_q, e), 148.6 (C_q, d), 146.7 (C_q, k), 146.3 (C_q, c), 146.0 (C_q, f), 145.5 (C_q, g), 145.1 (CH, A), 140.8 (2 x C_q, C_{BODIPY}), 131.4 (CH, NCH), 130.7 (2 x C_q, m), 121.7 (2 x CH, n), 117.1 (2 x CH, C, D), 115.3 (2 x CH, C, D), 112.3 (CH, B), 112.1 (CH, E), 95.7 (C_q, h), 70.2 (CH_2), 70.0 (CH_2, H), 69.8 (CH_2), 69.7 (CH_2), 69.5 (CH_2), 68.8 (CH_2, I), 67.6 (CH_2, G), 56.5 (CH_2, L), 52.6 (2 x CH_2, M), 49.3 (2 x CH_2, K), 44.4 (CH_2, F), 38.2 (CH_2, J), 37.8 (CH_2, N), 31.1 (CH_2, Q), 28.8 (CH_2, O), 27.8 (CH_2, R), 27.0 (CH_2, P), 15.8 (2 x CH_3), 14.1 (2 x CH_3). Mp: 171 °C (decomposition). **HRMS** (ESI-QTOF) calculated for $C_{52}H_{68}BF_2N_{13}O_8$ $[M+H]^+$: 1052.5456; found: 1052.5324. Purity: 95%. Absorption/emission: 498 nm / 508 nm.

1.2 Expression of A_{2A}AR constructs in Sf9 insect cells

Sf9 insect cells (Expression Systems) were cultured in ESF921 cell culture medium (Expression Systems) at 27 °C and were frequently tested negative for mycoplasma contamination (PCR-test, in-house). A_{2A}AR constructs were expressed utilizing a baculoviral expression system (Bac-to-Bac, ThermoFisher). For this purpose, the DNA sequence encoding for the wt A_{2A}AR and the A_{2A}-ΔC construct (comprising A_{2A}AR residues 2-316) was cloned into a modified pFastBac1 vector (ThermoFisher) that has its original polyhedrin promoter substituted with a GP64 promoter (GP64-pFastBac1).[2] The overall expression cassette is identical to previously described A_{2A}AR crystallization constructs[3] including an *N*-terminal auto-cleavable influenza hemagglutinin (HA) signal sequence[4] followed by a FLAG-tag and a *C*-terminal deca-histidine tag. The *C*-terminal histidine tag is not present in the wt A_{2A}AR plasmid. For A_{2A}-ΔC-bRIL, the third intracellular loop (residues 209-218) of A_{2A}-ΔC was substituted with a thermostabilized apocytochrome bRIL.[5] The S91$^{3.39}$K point mutation was introduced using site-directed mutagenesis to create A_{2A}-PSB1-bRIL and A_{2A}-PSB1. The DNA sequence encoding for the A_{2A}-StaR2-bRIL[6] comprising nine point mutations (A54$^{2.42}$L, T88$^{3.36}$A, R107$^{3.55}$A, K122$^{4.43}$A, N154^{ECL2}A, L202$^{5.63}$A, L235$^{6.37}$A, V239$^{6.41}$A, S277$^{7.42}$A) was gene-synthesized by BioCat and subcloned into the same GP64-pFastBac1 vector. The final plasmids were transfected into Sf9 insect cells at a cell density of 1.0 mio cells per ml as previously described [7] to generate the initial P0 virus. 400 µl of the P0 viral solutions were used to infect 40 ml of Sf9 insect cells at a density of 2.0-3.0 mio cells per ml following incubation at 27 °C for 48 h at 140 revolutions per minute. Both, the P1 virus and cells expressing the different A_{2A}AR constructs were harvested by centrifugation. Proteins purified from these cells were used to assess protein stability. For upscaling, 6 ml of P1 viruses were used to infect 900 ml of Sf9 insect cells at a density of 2.0-3.0 mio cells per ml following incubation at 27 °C for 48 h at 140 revolutions per minute. Cells were harvested by centrifugation, washed with phosphate-buffered saline (PBS) and stored at -80 °C for further use.

1.3 Protein purification for crystallization experiments

Protein purification for crystallization experiments was performed according to previously described procedures.[3] Sf9 insect cells from 900 ml infected cell culture were lyzed by osmotic shock in low osmotic buffer [10 mM HEPES pH 7.5, 10 mM $MgCl_2$, 20 mM KCl and EDTA-free cOmplete protease inhibitor cocktail (Roche)] using a dounce homogenizer and washed repeatedly using a high osmotic buffer that is identical to the low osmotic buffer but with the addition of 1 M NaCl. Membranes were finally resuspended in 50 ml resuspension buffer [10 mM HEPES pH 7.5, 10 mM $MgCl_2$, 20 mM KCl, 30% (v/v) glycerol] and stored at -80 °C for further use. Purified membranes corresponding to a volume of 25 ml were incubated with 4 mM theophylline and 2 mg per ml iodoacetamide for 60 min. The A_{2A}AR was then solubilized using an equal volume of solubilization buffer I [100 mM HEPES pH 7.5, 1.6 M NaCl, 1.0 % (w/v) dodecyl-β-d-maltopyranoside (DDM) and 0.2% (w/v) cholesteryl hemisuccinate (CHS)] over 3 h while shaking (end-over-end) at 4 °C. Solubilized proteins were separated from insoluble material by centrifugation at 48,000 g. The supernatant was supplemented with 20 mM imidazole (aqueous solution pH 7.5) and 500 µl of a pre-washed cobalt-based immobilized metal affinity chromatography (IMAC) medium (TALON Superflow, cytiva) following overnight incubation at 4 °C. In order to remove protein impurities and to exchange the low affinity A_{2A}AR antagonist theophylline for PSB-2113 or PSB-2115, the IMAC medium was washed with 30 column volumes (CVs) of wash buffer I [50 mM HEPES pH 7.5, 800 mM NaCl, 0.5 % (w/v) DDM, 0.1% (w/v) CHS, 10% (v/v) glycerol, 20 mM imidazole pH 7.5, 8 mM adenosine triphosphate (ATP), 10 mM $MgCl_2$, and 50 µM PSB-2113 or 50 µM PSB-2115] and 20 CVs of wash buffer II [50 mM HEPES pH 7.5, 800 mM NaCl, 0.05 % (w/v) DDM, 0.01% (w/v) CHS, 10% (v/v) glycerol, 50 mM imidazole pH 7.5, and 50 µM PSB-2113 or 50 µM PSB-2115]. A_{2A}-PSB1-bRIL-PSB-2113 and A_{2A}-PSB1-bRIL-PSB-2115 complexes were eluted from the column using 4 CVs elution buffer [25 mM HEPES pH 7.5, 800 mM NaCl, 10% (v/v) glycerol, 220 mM imidazole, 0.025 % (w/v) DDM, 0.005% (w/v) CHS, and 25 µM PSB-2113 or 25 µM PSB-2115]. The purified receptor complex was concentrated to volume of 20-30 µl using 100 kDa cut-off Vivaspin concentrators (Sartorius). The protein was immediately used for crystallization experiments while protein purity, monodispersity and thermostability were assessed using analytical size-exclusion chromatography, sodium dodecyl sulfate polyacrylamide gel electrophoresis (SDS-PAGE) and a thermal shift assay, respectively.

1.4 Protein purification for stability screening

A_{2A}ARs from 40 ml of Sf9 insect cell culture were purified accordingly but with minor modifications. Cells were lyzed by osmotic shock in low osmotic buffer and washed once with high osmotic buffer. Purified membranes were resuspended in 3 ml resuspension buffer and stored at -80 °C until further use. Prior to solubilization, membranes were incubated with 2 mg per ml iodoacetamide for 30 min at 4 °C. No ligand was added during purification so that the A_{2A}ARs were obtained in their APO state. Solubilization was performed using an equal volume of solubilization buffer II [100 mM HEPES pH 7.5, 1.6 M NaCl, 2.0 % (w/v) DDM and 0.4% (w/v) CHS] over the time course of 3 h while shaking (end-over-end) at 4 °C. Solubilized proteins were obtained by centrifugation at 14,000 g. The supernatant was supplemented with 20 mM imidazole pH 7.5 and 12.5 µl cobalt-based IMAC medium (TALON Superflow, cytiva) following overnight incubation at 4 °C. The IMAC medium was washed with 60 CVs of ligand-free wash buffer I (without the addition of ATP and $MgCl_2$) and 40 CVs of ligand-free wash buffer II. Finally, the proteins were eluted using 10 CVs of ligand-free elution buffer without further protein concentration. The protein was used directly for analytical size-exclusion chromatography, SDS-PAGE and thermal shift assays.

1.5 Thermal shift assay

The thermostability of different A_{2A}AR protein preparations was determined as previously described[8] using the thiol-specific fluorescent dye *N*-[4-(7-diethylamino-4-methyl-3-coumarinyl)phenyl]maleimide (CPM). Briefly, CPM (final concentration 2 µg per ml) was incubated for 10 min with an appropriate amount of protein in a buffer consisting of 25 mM HEPES pH 7.5, 500 mM NaCl, 2% (v/v) glycerol, 0.05% (w/v), 0.01% (w/v) CHS and the tested A_{2A}AR ligand (ligand stock solutions were prepared at a concentration of 1 mM in DMSO) or control. The protein thermostability was assessed on a Rotor-Gene Q real-time PCR cycler (Qiagen) using the excitation wavelength of 365 ± 20 nm and the detection wavelength of 460 ± 20 nm. Data was collected over a temperature range of 30 °C to 90 °C with a ramp of 1 °C per min and a fluorescence gain of 1. The T_M values were calculated from the turning point of the non-linear regression (Boltzmann sigmoidal fit) in GraphPad Prism 7.0 after subtraction of a respective buffer control value. Thermostability data was obtained from at least three independent measurements.

1.6 Crystallization

The purified and concentrated A_{2A}-PSB1-bRIL-PSB-2113 and A_{2A}-PSB1-bRIL-PSB-2115 complexes were reconstituted into LCP by mixing the protein with a molten lipid mixture [90% (w/v) 1-oleoyl-*rac*-glycerin (Sigma), 10 % (w/v) cholesterol (Sigma)] in a 2 to 3 ratio using the two-syringe method.[9] An automatic crystallization robot (Formulatrix NT8) was used to perform crystallization experiments by overlaying 50 nl mesophase with 0.8 nl precipitant solution on 96-well glass sandwich plates (Marienfeld). The crystallization plates were stored at 20 °C and automatically imaged using a crystallization plate imager (Formulatrix RockImager 54). Diffraction quality crystals grew under the following precipitant condition: 24% (v/v) PEG-400 (polyethylene glycol 400, average molecular weight 400, Hampton Research), 10-30 mM sodium thiocyanate (Hampton Research), 100 mM sodium citrate pH 5.2 (Hampton Research), and 2% (v/v) 2,5-hexanediol (Molecular Dimensions). Crystals were harvested using 50-100 µm Micromounts (MiTeGen) and were directly flash-frozen in liquid nitrogen without further cryoprotection.

1.7 Data collection and structure determination

X-ray diffraction data were collected on the P11-high-throughput-MX beam line at PETRA III, Hamburg, Germany. Data were collected at 100 K using a microfocused beam (20 × 20 µm^2) of ~12.0 keV (1.0332 Å) with 1 % transmission at a rate of 100 ms per frame and an oscillation-range of 0.1°. Data were collected until 1.8 Å and 2.5 Å for the A_{2A}-PSB1-bRIL-PSB-2113 and A_{2A}-PSB1-bRIL-PSB-2115 crystals, respectively. XDS, XSCALE and XDSCONV[10] were used for data processing (see **Table S2** for details). The PSB-2113 structure was determined by phenix.phaser [11] using the previously solved A_{2A}-ΔC-bRIL structure[3] as a model (PDB 4EIY) (translation function Z score (TFZ) - 67.0 and log-likelihood gain (LLG) - 9667.819). The PSB-2115 structure was determined similarly (TFZ - 61.2 and LLG - 6257.201), but instead using the A_{2A}-PSB1-bRIL-PSB-2113 coordinates as the search model. Each model went through phenix.autobuild[12] once, which included density modification, iterative-model building and refinement. Then, each model was built with respective ligands (PSB-2113 and PSB-2115) and other components like cholesterol hemisuccinate, monoolein and PEG using COOT.[13] The newly determined protein structures were consistent with the published PDB 4EIY structure, the RMSD being 0.28 Å. For the ligands, well-defined electron densities were observed within the orthosteric binding site, while no significant electron density was observed for the fluorophores and the connecting flexible PEG linker. The refined structures of the PSB-2113 and PSB-2115 complexes showed good agreement with the obtained data (R_{work} / R_{free} ratios of 0.190 / 0.235 and 0.187 / 0.245 (2.6 Å), respectively). Detailed refinement statistics are reported in **Table S2**.

1.8 Radioligand binding assays

Radioligand binding assays were performed on Sf9 insect cell and CHO-S cell membranes. Sf9 membrane preparations were obtained from 40 ml of baculovirus infected Sf9 insect cells using a dounce homogenizer with one step low osmotic buffer and one step high osmotic buffer as described above, but resuspended in the following resuspension buffer: 10 mM HEPES pH 7.5, 10 mM $MgCl_2$, 20 mM KCl. CHO-S cells were disrupted in a buffer consisting of 50 mM tris(hydroxymethyl)aminomethane (Tris) pH 7.4 and 2 mM EDTA using an Ultra-Turrax homogenizer and cell membranes were resuspended in a 50 mM Tris pH 7.4 buffer. Total protein concentrations were determined using a Bradford assay[14] with bovine serum albumin (BSA) as a reference, and 15 µg of protein per well was used in the radioligand binding experiments. Competition binding experiments to determine the affinity (K_i) of A_{2A}AR ligands were performed using the A_{2A}AR-selective antagonist radioligand [^{3}H]MSX-2 (specific activity 85 Ci per mmol, final concentration 1 nM)[15] in a buffer consisting of 50 mM Tris buffer pH 7.4 (supplemented with 2 U per ml adenosine desaminase). Nonspecific binding was determined in the presence of 10 µM CGS15943, and total binding was determined in the presence of DMSO. The assays were incubated for 30 min at RT followed by filtration through GF/B glass fiber filters (Whatman) using a 48-well harvester (Brandel). Filters were pre-incubated for 30 to 60 min in a solution of 0.3 % (w/v) polyethylenimine to reduce nonspecific binding. After harvesting, filters were washed with ice-cold Tris buffer (50 mM Tris, pH 7.4), transferred into scintillation vials, and incubated with 2.5 ml of scintillation cocktail (ProSafe FC plus) for 6 h. Subsequently, the radioactivity was determined on a liquid scintillation counter (Tricarb 2810TR, Perkin Elmer, efficiency of 53%). At least three independent experiments were performed. Homologous competition with unlabeled MSX-2 was

performed to determine the equilibrium dissociation constant (K_d) of each A_{2A}AR construct. K_i values of A_{2A}AR antagonists and agonists were calculated using the Cheng-Prusoff equation (GraphPad Prism 7.0).

1.9 TRUPATH assay

Human embryonic kidney 293 (HEK293) cells were cultured at 37° C and 5% CO_2 in Dulbecco's modified eagle's medium (DMEM) supplemented with 10% (v/v) fetal bovine serum (FBS), 100 U penicillin per ml, and 100 µg streptomycin per ml. Repeated in-house PCR tests confirmed the cells to be free of mycoplasma contamination.

The TRUPATH bioluminescence resonance energy transfer (BRET) assay was conducted with minor variations to the originally published protocol.[16] TRUPATH biosensors were a gift from Bryan Roth (Addgene kit #1000000163). On the first day, HEK293 cells were detached from the flask with trypsin and seeded into 6-well plates at a density of 10^6 cells per well in 2 ml DMEM. After 2 h, the cells were transiently transfected with the biosensors and the GPCR of interest. For this purpose, the A_{2A}AR constructs with or without bRIL fusion protein were PCR-amplified without *N*-terminal HA- and *C*-terminal His-tags and subcloned into pcDNA3.1(+) using BamHI and EcoRI. For each well, 3 µl lipofectamine 2000 (ThermoFisher) per µg DNA were mixed with OptiMEM medium (ThermoFisher) to a volume of 250 µl per well and then incubated for 10 min at RT. Then, the lipofectamine solution was mixed with an equal volume of OptiMEM-DNA mixture (100 ng of each pcDNA5/FRT/To-$G\alpha_{s\text{-}short}$-RLuc8, pcDNA3.1-$G\beta_3$, pcDNA3.1-$G\gamma_9$-GFP2, and pcDNA3.1-FLAG-GPCR) and incubated for 30 min at RT. The mixture was added to the cells following overnight incubation. The next day, the cells were detached from the 6-well plate by pipetting and were seeded into a white-bottom 96-well plate (Greiner BioOne) at a density of 30,000 cells per well. After 24 h, the cells were washed with assay buffer (Hank's balanced salt solution (ThermoFisher) plus 20 mM HEPES pH 7.4). Then, 60 µl of assay buffer supplemented with 1000 U per ml of adenosine desaminase (Roche) and 10 µl of luciferase substrate (50 µM coelenterazine 400a (Biomol) in assay buffer) was added to the cells and incubated for another 5 min. Agonist solution (30 µl, 1% final DMSO concentration) was added and the BRET measurement was started in a Mithras LB940 plate reader after 5 min (RLuc8 emission at 395 nm, GFP2 emission at 510 nm). The BRET2 ratio was calculated by division of the GFP2 signal by the RLuc8 signal. Data analysis was performed by means of GraphPad PRISM 8.0. netBRET values were calculated by subtraction of buffer control BRET2 ratio from the BRET2 ratio of each data point. Concentration-response curves were then fitted by a sigmoidal dose-response curve with variable slope (four parameters) to yield EC_{50} and E_{max} values. Data was obtained in at least three independent experiments performed in duplicate.

1.10 Synthesis of carboxy-functionalized Preladenant analog 2

The synthesis of the precursor **2** is depicted in **Scheme S1** below.

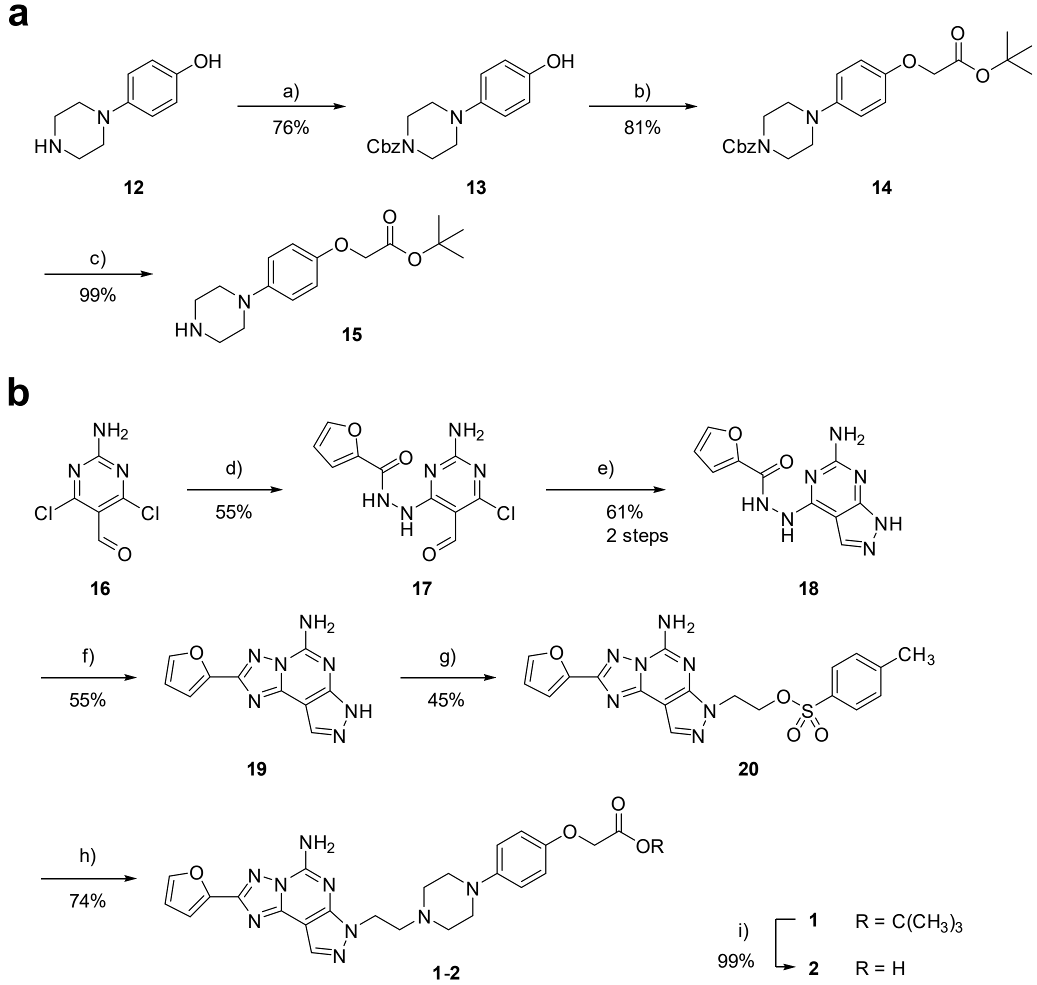

Scheme S1. a Synthesis of the piperazine adduct **15**. **b** Synthesis of the carboxy-functionalized Preladenant derivative **2**.
Reaction conditions: a) CbzCl, MeOH, RT, 12h. b) *tert*-butyl bromoacetate, K_2CO_3, acetone, RT, 12 h. c) 10% Pd/C, H_2, MeOH, RT, 2h. d) 2-Furoic acid hydrazide, NEt_3, DMSO, 120 °C, 18 h. e) N_2H_4, CH_3CN, 70 °C, 1h. f) BSA, HMDS, 120 °C, 3h. g) Ethylene glycol ditosylate, NaH, DMF, RT, 4h. h) **4**, DMF, 80 °C, 20h. i) trifluoroacetic acid, CH_2Cl_2, RT, 1h.

1.10.1 Synthesis of the side-chain (a)

Benzyl 4-(4-hydroxyphenyl)piperazine-1-carboxylate (13)

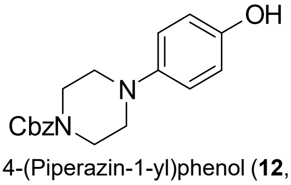

4-(Piperazin-1-yl)phenol (**12**, 500 mg, 2.8 mmol, 1.0 eq.) is dissolved in 2.8 ml of methanol, and benzyl chloroformate (402 µl, 2.8 mmol, 1.0 eq.) is added dropwise. The reaction mixture is stirred for 12h at RT. Then, the solvent is removed *in vacuo*, and the residue is dissolved in 50 ml of a saturated aq. $NaHCO_3$ solution. After extraction with ethyl acetate (3 x 50 ml) the united organic phases are dried over $MgSO_4$, and the solvent is subsequently removed *in vacuo*. The product is purified by column chromatography on silica gel with dichloromethane/methanol (95:5) yielding 661 mg (2.1 mmol) of **13** (yield: 76 %).
^{1}H NMR (500 MHz, DMSO-d_6) δ [ppm]: 8.85 (s, 1H, OH), 7.42–7.28 (m, 5H, H_{Cbz}), 6.85–6.75 (m, 2H, $H_{arom.}$), 6.70–6.61 (m, 2H, $H_{arom.}$), 5.10 (s, 2H, H_{Cbz}), 3.59–3.45 (m, 4H, $H_{Piperazin}$), 2.94–2.89 (m, 4H, $H_{Piperazin}$). **^{13}C NMR** (126 MHz, DMSO-d_6) δ [ppm] = 154.4 (C_q, CO), 151.4 (C_q, COH), 144.0 (C_q, NC), 136.9 (C_q, C_{Cbz}), 128.4 (2x CH, C_{Cbz}), 127.8 (C_q, C_{Cbz}), 127.5 (2x CH, C_{Cbz}), 118.5 (C_q, $C_{arom.}$), 115.5 (C_q, $C_{arom.}$), 66.2 (CH_2, C_{Cbz}), 50.2 (2x CH_2, $C_{Piperazin}$), 43.6 (2x CH_2, $C_{Piperazin}$). **LC-MS**: 313.2 ([M+H]$^+$). **Purity**: 95%.

Benzyl 4-(4-(2-(*tert*-butoxy)-2-oxoethoxy)phenyl)piperazine-1-carboxylate (14)

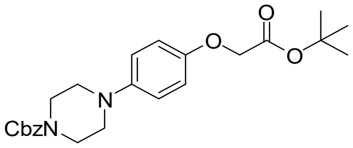

Compound **13** (100 mg, 0.32 mmol, 1.0 eq.) is dissolved in acetone (500 µl), and K_2CO_3 (49 mg, 0.35 mmol, 1.1 eq.) und *tert*-butyl bromoacetate (62 mg, 0.32 mmol, 1.0 eq.) are added under an atmosphere of argon. After a reaction time of 12 h at RT, the solvent is removed *in vacuo*, and the residue is dissolved in 5 ml of water, and subsequently extracted with ethyl acetate (3 x 20 ml). The united organic phases are dried over $MgSO_4$, and the solvent is subsequently removed *in vacuo*. The product is purified by column chromatography on silica gel with dichloromethane/methanol (97:3) yielding 110 mg (2.6 mmol) of **14** (yield: 81 %).

^{1}H NMR (500 MHz, DMSO-*d*$_6$) δ [ppm]: 7.40–7.30 (m, 5H, H_{Cbz}), 6.93–6.87 (m, 2H, $H_{arom.}$), 6.84–6.76 (m, 2H, $H_{arom.}$), 5.10 (s, 2H, H_{Cbz}), 4.54 (s, 2H, CH_2), 3.58–3.48 (m, 4H, $H_{Piperazin}$), 3.02–2.95 (m, 4H, $H_{Piperazin}$), 1.42 (s, 9H, 3x CH_3). **^{13}C NMR** (126 MHz, DMSO-*d*$_6$) δ [ppm] = 168.1 (C_q, $CO_{t\text{-Butylester}}$), 154.4 (C_q, CO_{Cbz}), 151.7 (C_q, C_{Ether}), 145.6 (C_q, CN), 136.8 (C_q, C_{Cbz}), 128.4 (2x CH, C_{Cbz}), 127.8 (C_q, C_{Cbz}), 127.5 (2x CH, C_{Cbz}), 117.8 (C_q, $C_{arom.}$), 115.0 (C_q, $C_{arom.}$), 81.2 (C_q, $C(CH_3)_3$), 66.2 (CH_2, C_{Cbz}), 65.5 (CH_2), 49.6 (2x CH_2, $C_{Piperazin}$), 43.4 (2x CH_2, $C_{Piperazin}$), 27.7 (3x CH_3). **LC-MS**: 427.2 ($[M+H]^+$). **Purity**: 98%.

***tert*-Butyl 2-(4-(piperazin-1-yl)phenoxy)acetate (15)**

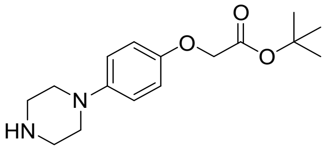

Compound **14** (100 mg, 0.23 mmol, 1.0 eq.) is dissolved in methanol (1 ml). Under an atmosphere of argon (using an argon-filled ballon), 10% Pd/C (10 mg) is added. Then, the argon atmosphere is replaced by an atmosphere of hydrogen gas (H_2) again using a balloon (a T-connector is used for switching between the two gas-containing balloons), and the solution is stirred for 2h at RT. The solution is filtered over Celite®, and the solvent is removed *in vacuo*. The product is obtained in 99% yield (67 mg, 0.23 mmol).

^{1}H NMR (600 MHz, DMSO-*d*$_6$) δ [ppm]: 6.88–6.82 (m, 2H, $H_{arom.}$), 6.80–6.75 (m, 2H, $H_{arom.}$), 4.53 (s, 2H), 2.95–2.91 (m, 4H, $H_{piperazine}$), 2.86–2.81 (m, 4H, $H_{piperazine}$), 1.42 (s, 9H, 3 x CH_3). **^{13}C NMR** (151 MHz, DMSO-*d*$_6$) δ [ppm] = 168.2 (C_q, $CO_{t\text{-Butylester}}$), 151.1 (C_q, C_{Ether}), 146.4 (C_q, CN), 117.1 (C_q, $C_{arom.}$), 115.0 (C_q, $C_{arom.}$), 81.2 (C_q, $C(CH_3)_3$), 65.5 (CH_2), 50.3 (2x CH_2, $C_{piperazine}$), 45.5 (2 x CH_2, $C_{piperazine}$), 27.7 (3 x CH_3). **LC-MS**: 293.0 ($[M+H]^+$). **Purity**: 97%.

1.10.2 Synthesis of the tricyclic Preladenant core (19) and attachment of the side chain yielding the functionalized Preladenant derivative 2 (b)

(*E*/*Z*)-*N*'-(6-Chloro-5-formyl-2-imino-2,3-dihydropyrimidin-4(1*H*)-ylidene)furan-2-carbohydrazide (17)[17]

2-Amino-4,6-dichloropyrimidine-5-carbaldehyde (**16**, 1,0 g, 5.2 mmol, 1 eq.) is dissolved in 40 ml of THF, and furane-2-carbonic acid hydrazide (730 mg, 5.72 mmol, 1.1 eq.) and triethylamine (526 mg, 5.2 mmol, 1 eq.) are added under an atmosphere of argon. After 2 h of heating under reflux, the solvent is removed *in vacuo*, and water is added (20 ml). The aqueous solution is extracted with EtOAc (5 x 20 ml), and the united organic phases are dried over $MgSO_4$. After filtration, the solvent is removed *in vacuo*. The remaining solid is recrystallized from methanol/diethyl ether (8:1) yielding 811 mg (2.9 mmol, 55%) of **17** (lit. yield: 82% [17]).

^{1}H NMR (500 MHz, DMSO-*d*$_6$) δ [ppm]: 10.64 (s, 1H), 10.38 (s, 1H), 9.97 (s, 1H, $H_{aldehyde}$), 7.92–7.90 (m, 1H, H_{furane}), 7.88 (s, 1H), 7.85 (s, 1H), 7.25 (d, $^3J_{H,H}$ = 3.54 Hz, 1H, H_{furane}), 6.67 (dd, $^3J_{H,H}$ = 1.76 Hz, $^3J_{H,H}$ = 3.48 Hz, 1H, H_{furane}). **^{13}C NMR** (126 MHz, DMSO-*d*$_6$) δ [ppm] = 187.5 (CH, $C_{aldehyde}$), 165.0 (Cq), 163.0 (Cq), 162.5 (Cq), 156.5 (Cq), 146.0 (Cq), 145.7 (CH, C_{furane}), 114.9 (CH, C_{furane}), 111.9 (CH, C_{furane}), 100.8 (Cq). **LC-MS**: positive [*m*/*z*] = 282.1 ($[M+H]^+$). **Purity**: 98%.

***N*'-(6-Amino-1*H*-pyrazolo[3,4-*d*]pyrimidin-4-yl)furane-2-carbohydrazide (18)**

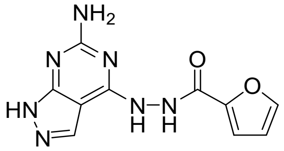

Compound **17** (0.5 g, 1.7 mmol, 1.0 eq.) is dissolved in 25 ml of acetonitrile, heated to 70 °C, and hydrazine (80% aqueous solution, 323 mg, 5.1 mmol, 3.0 eq.) is added. After 1 h of heating under reflux, the resulting solid is filtered off and washed with acetonitrile (ca. 25 ml) yielding 250 mg (0.96 mmol, 57%) of **18** (lit. yield: 83%[17]).

^{1}H NMR/^{13}C NMR in 2M-DCl/DMSO-d_6: signals for 3 tautomers are observed which cannot be unambigously assigned. **LC-MS**: positive [*m*/*z*] = 260.2 ([M+H]$^+$). **Purity**: 99%.

5-Amino-2-(furan-2-yl)-7*H*-pyrazolo[4,3-*e*][1,2,4]triazolo[1,5-*c*]pyrimidine (19)

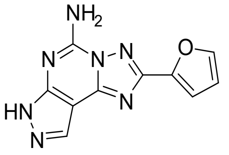

Compound **18** (200 mg, 0.77 mmol, 1 eq.), *N*,*O*-bis(trimethylsilyl)acetamide (1.08 g, 5.3 mmol, 6.8 eq.) and hexamethyldisilazane (2.85 g, 17.7 mmol, 23 eq.) are heated under an atmosphere of argon at 120 °C for 3h. The residue is suspended in hot water and filtered. The residue is recrystallized from 80% aqueous acetic acid yielding 94 mg (0.4 mmol, 51 % yield) of **19** (lit. yield 67% [17]).

^{1}H NMR (600 MHz, DMSO-d_6) *δ* [ppm]: 13.37 (bs, 1H, NH), 8.14 (bs, 1H, NCH), 7.94–7.93 (m, 1H, H_{Furan}), 7.92 (bs, 2H, NH_2) 7.22 (d, $^3J_{H,H}$ = 3.31 Hz, 1H, H_{Furan}), 6.73 (dd, $^3J_{H,H}$ = 1.73, $^3J_{H,H}$ = 3.38 Hz, 1H, H_{Furan}). **^{13}C NMR** (151 MHz, DMSO-d_6) *δ* [ppm] =155.2 (C_q), 150.2 (C_q), 148.7 (C_q), 146.2 (C_q), 145.6 (CH, C_{furane}), 145.0 (CH, C_{furane}), 132.4 (CH, NCH), 112.1 (CH, C_{furane}), 112.1 (CH, C_{furane}), 95.4 (C_q). **LC-MS**: positive [*m*/*z*] = 242.1 ([M+H]$^+$). **Purity**: 99%.

2-(5-Amino-2-(furan-2-yl)-7*H*-pyrazolo[4,3-*e*][1,2,4]triazolo[1,5-*c*]pyrimidin-7-yl)ethyl 4-methylbenzenesulfonate (20)[18]

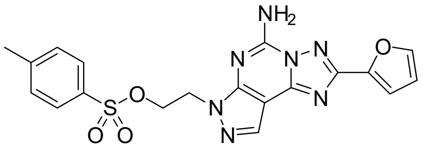

Compound **19** (50 mg, 0.21 mmol, 1.0 eq.) is dissolved in DMF (252 µl) under an atmosphere of argon, and NaH (10 mg, 0.25 mmol, 1.2 eq.), followed by ethylene di(*p*-toluenesulfonate) (95,5 mg, 0.25 mmol, 1.2 eq.), and the mixture is stirred for 4h at RT. The solvent is removed *in vacuo*, and the remaining solid is purified by flash chromatography on silica gel, applying a gradient from 99% dichloromethane/1% methanol to 95% dichloromethane/5% methanol within 20 min, yielding 42 mg (0.1 mmol, 45% yield) of **20**.

^{1}H NMR (600 MHz, DMSO-d_6) *δ* [ppm]: 8.11–8.00 (bs, 2H, NH_2), 8.04 (s, 1H, NCH), 7.97–7.94 (m, 1H, H_{furane}), 7.43–7.38 (m, 2H, $H_{arom.}$), 7.25 (d, $^3J_{H,H}$ = 3.36 Hz, 1H, H_{furane}), 7.11–7.07 (m, 2H, $H_{arom.}$), 6.74 (dd, $^3J_{H,H}$ = 1.77 Hz, $^3J_{H,H}$ = 3.38 Hz, 1H, H_{furane}), 4.55–4.52 (m, 2H, OCH_2), 4.51–4.47 (m, 2H, NCH_2), 2.13 (s, 3H, CH_3). **^{13}C NMR** (151 MHz, DMSO-d_6) *δ* [ppm] = 155.4 (C_q), 148.6 (C_q), 148.5 (C_q), 146.2 (C_q), 145.5 (C_q, C_{Furan}), 145.2 (CH, C_{furane}), 144.5 (C_q, C_{tosyl}), 131.7 (CH, NCH), 131.7 (C_q, C_{tosyl}), 129.4 (2 x CH_2, $C_{arom.}$), 127.1 (2 x CH_2, $C_{arom.}$), 112.3 (CH, C_{furane}), 112.1 (CH, C_{furane}), 95.9 (C_q), 68.1 (CH_2, OCH_2), 46.0 (CH_2, NCH_2), 20.8 (CH_3, C_{methyl}). **LC-MS**: positive [*m*/*z*] = 440.2 ([M+H]$^+$). **Purity**: 98%.

***tert*-Butyl 2-(4-(4-(2-(5-amino-2-furan-2-yl)-7*H*-pyrazolo[4,3-*e*][1,2,4]triazolo[1,5-*c*]pyrimidin-7-yl)ethyl)piperazin)-1-yl)phenoxy)acetate (1)**

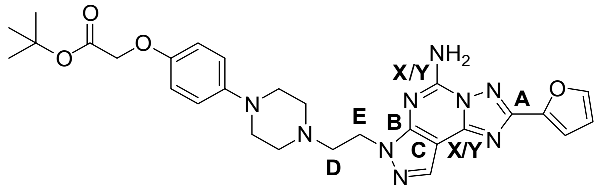

Compound **20** (39 mg, 0.09 mol, 1.0 eq.) is dissolved in DMF (504 µl) under an atmosphere of argon, and *tert*-butyl 2-(4-piperazin-1-yl)phenoxy)acetate (52 mg, 0.18 mmol, 2.0 eq.) is added. After 20h of stirring at 80 °C the solvent is removed *in vacuo*, and the product is purified by flash chromatography as described for compound 20 (the same gradient is run, however within 25 min). Yielding 24 mg (0.04 mmol) of **1** (yield: 48 %).

^{1}H NMR (600 MHz, DMSO-d_6) *δ* [ppm: 8.16 (s, 1H, NCH), 8.05 (bs, 2H, NH_2), 7.96–7.90 (m, 1H, H_{furane}), 7.22 (d, $^3J_{H,H}$ = 3.39 Hz, 1H, H_{furane}), 6.87–6.82 (m, 2H, 2x $CH_{arom.}$), 6.80–6.74 (m, 2H, 2 x $CH_{arom.}$), 6.73 (dd, $^3J_{H,H}$ = 1.76 Hz, $^3J_{H,H}$ = 3.34 Hz, 1H, H_{furane}), 4.52 (s, 2H, CO_2CH_2), 4.42 (t, $^3J_{H,H}$ = 6.89 Hz, 2H, NCH_2), 3.02–2.90 (m, 4H, 2x $CH_{2xpiperazine}$), 2.83 (t, $^3J_{H,H}$ = 6.94 Hz, 2H, $N_{piperazine}CH_2$), 2.65–2.54 (m, 4H, 2x $CH_{2xpiperazine}$), 1.41 (s, 9H, 3 x CH_3). **^{13}C NMR** (151 MHz, DMSO-d_6) *δ* [ppm] = 168.1 (C_q, $CO_{t\text{-}Butylester}$), 155.3 (C_q, A), 151.2 (C_q, C_{ether}), 148.7 (C_q, X/Y), 148.6 (C_q, B), 146.2 (C_q, X/Y), 145.8 (C_q, $CN_{piperazine}$), 145.5 (C_q, C_{furane}), 145.1 (CH, C_{furane}), 131.4 (CH, NCH), 117.0 (C_q, $C_{arom.}$), 115.0 (C_q, $C_{arom.}$), 112.2 (CH, C_{furane}), 112.1 (CH, C_{furane}), 95.6 (C_q, C), 81.1 (C_q, $C(CH_3)_3$), 65.5 (CH_2, CO_2CH_2), 56.5 (CH_2, D), 52.6 (2x CH_2, $C_{piperazine}$), 49.3 (2 x CH_2, $C_{piperazine}$), 44.4 (CH_2, E), 27.7 (3 x CH_3). Mp. 192.9–196.8 °C. **LC-MS**: positive [*m*/*z*] = 560.1 ([M+H]$^+$). **HRMS** (ESI-QTOF) calculated for $C_{28}H_{33}N_9O_4$ [M+H]$^+$: 560.2728; found: 560.2725. **Purity**: 97%.

2-(4-(4-(2-(5-Amino-2-(furan-2-yl)-7*H*-pyrazolo[4,3-*e*][1,2,4]triazolo[1,5-*c*]pyrimidin-7-yl)ethyl)piperazin-1-yl)phenoxy)acetic acid (2)

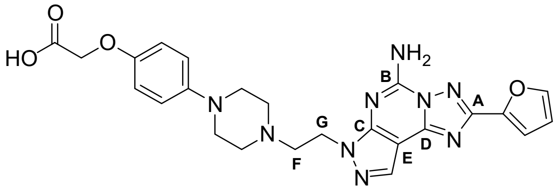

Compound **1** (21 mg, 0.038 mmol, 1.0 eq.) is dissolved in a round bottom flask in 10 ml of dichloromethane, and trifluoroacetic acid (1.2 ml) is added dropwise upon cooling with ice. Then, the ice bath is removed, and 2 drops of water are added. After stirring for 24h at RT, the solvent is removed *in vacuo*, and the residue is purifed by HPLC, applying a gradient of 50% aq. acetonitrile to 100% acetonitrile within 15 min, followed by elution for 5 min with 100% acetonitrile, yielding 19 mg (0.038 mmol) of **2** (yield: 100%).
^{1}H NMR (600 MHz, DMSO-d_6) *δ* [ppm]: 8.16 (s, 1H, NCH), 8.05 (bs, 2H, NH_2), 7.93 (d, $^3J_{H,H}$ = 1.6 Hz, 1H, H_{furane}), 7.22 (d, $^3J_{H,H}$ = 3.37 Hz, 1H, H_{furane}), 6.88–6.81 (m, 2H, 2 x $CH_{arom.}$), 6.79–6.75 (m, 2H, 2 x $CH_{arom.}$), 6.73 (dd, $^3J_{H,H}$ = 1.76 Hz, $^3J_{H,H}$ = 3.37 Hz, 1H, H_{furane}), 4.52 (s, 2H, CO_2CH_2), 4.42 (t, $^3J_{H,H}$ = 6.85 Hz, 2H, NCH_2), 2.99–2.92 (m, 4H, 2 x $CH_{2\ x\ piperazine}$), 2.84 (t, $^3J_{H,H}$ = 6.85 Hz, 2H, $N_{üiperazine}CH_2$), 2.64–2.57 (m, 4H, 2 x $CH_{2\ x\ piperazine}$). **^{13}C NMR** (151 MHz, DMSO-d_6) *δ* [ppm] = 170.5 (C_q, COOH), 155.4 (C_q, A), 153.0 (C_q, C_{ether}), 148.7 (C_q, B), 148.6 (C_q, C), 146.3 (C_q, D), 145.5 (C_q, $CN_{piperazine}$), 145.5 (C_q, C_{furane}), 145.1 (CH, C_{furane}), 131.4 (CH, NCH), 117.2 (C_q, $C_{arom.}$), 114.8 (C_q, $C_{arom.}$), 112.3 (CH, C_{furane}), 112.2 (CH, C_{furane}), 95.7 (C_q, E), 68.3 (CH_2, CO_2CH_2), 56.6 (CH_2, F), 52.8 (2x CH_2, $C_{piperazine}$), 49.9 (2 x CH_2, $C_{piperazine}$), 44.4 (CH_2, G). **Mp** >300 °C. **LC-MS**: positive [*m*/*z*] = 504.2 ($[M+H]^+$). **HRMS** (ESI-QTOF) calculated for $C_{24}H_{25}N_9O_4$ $[M-H]^-$: 502.1950; found: 502.1943. **Purity**: 98%.

1.10.3 Synthesis of amino-substituted PEG derivatives 3a-3f

The following azido-functionalized PEG esters used as starting materials were synthesized as previously described[19,20] and reduced using Method A or Method B, respectively, to yield the desired amino-functionalized PEG derivatives.

1.10.3.1 Method A

The appropriate ester (1.0 eq.) is dissolved in dichloromethane (1 ml per 100 mg of ester) under an atmosphere of argon (using a ballon), and 10% Pd/C (10 mg per 100 mg of ester) is added. The argon atmosphere is exchanged for hydrogen gas (H_2) using a balloon, and the mixture is stirred for 8h at RT. Then, the solution is filtered through Celite®, and the solvent is subsequently removed *in vacuo*. The product is purified by column chromatography on silica gel using 7N ammonia solution in methanol/dichloromethane as eluent; for details see below.

***tert*-Butyl 14-amino-3,6,9,12-tetraoxatetradecanoate (3a)**

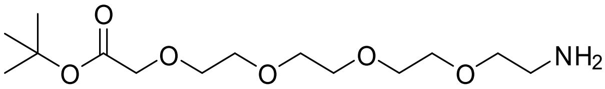

tert-Butyl 14-azido-3,6,9,12-tetraoxatetradecanoate (400 mg, 1.2mmol, 1.0 eq.); 7N NH_3 in methanol/dichloromethane (5:95); yield: 240 mg (0.78 mmol, 71%).
^{1}H-NMR (600 MHz, DMSO-d_6) *δ* [ppm]: 3.98 (s, 2H, CO_2CH_2), 3.58–3.55 (m, 2H, CH_2), 3.55–3.47 (m, 12H, 6 x CH_2), 3.36–3.33 (t, $^3J_{H,H}$ = 5.85 Hz, 2H, CH_2), 2.63 (t, $^3J_{H,H}$ = 5.83 Hz, 2H, NH_2), 1.42 (s, 9H, 3 x CH_3). **^{13}C-NMR** (151 MHz, DMSO-d_6) *δ* [ppm] = 169.4 (C_q, $C_{carbonyl}$), 80.7 (C_q, *C*$(CH_3)_3$), 73.0 (CH_2), 69.9 (CH_2), 69.8 (CH_2), 69.8 (CH_2), 69.8 (CH_2), 69.7 (CH_2), 69.6 (CH_2), 68.1 (CH_2), 41.3 (CH_2), 27.8 (3 x CH_3, $C(CH_3)_3$). **LC-MS**: positive [*m*/*z*] = 308.1 ($[M+H]^+$). **Purity** (determined by NMR): 95%.

***tert*-Butyl 17-amino-3,6,9,12,15-pentaoxaheptadecanoate (3b)**

tert-Butyl 17-azido-3,6,9,12,15-pentaoxaheptadecanoate (100 mg, 0.26 mmol, 1.0 eq.). The compound was used without further purification.
^{1}H-NMR (500 MHz, DMSO-d_6) *δ* [ppm]: 3.98 (s, 2H, $COOCH_2$), 3.58–3.48 (m, 18H, 9 x CH_2), 3.37–3.33 (m, 2H, CH_2), 2.64 (t, $^3J_{H,H}$ = 5.82 Hz, NH_2) 1.42 (s, 9H, 3x CH_3). **^{13}C-NMR** (151 MHz, DMSO-d_6) *δ* [ppm]: 169.3 (C_q, CO), 80.6 (C_q, $C_{t\text{-butyl}}$), 73.0 (CH_2, CO_2CH_2O), 69.8 (CH_2), 69.8 (CH_2), 69.8 (CH_2), 69.8 (CH_2), 69.8 (CH_2), 69.7 (CH_2), 69.7 (CH_2), 69.6 (CH_2), 68.1 (CH_2) 41.3 (CH_2, NH_2CH_2), 27.8 (CH_3, 3 x CH_3). **LC-MS**: positive [*m*/*z*] = 351.8 ($[M+H]^+$). Purity determined by NMR: >90%.

***tert*-Butyl 26-amino-3,6,9,12,15,18,21,24-octaoxahexacosanoate (3c)**

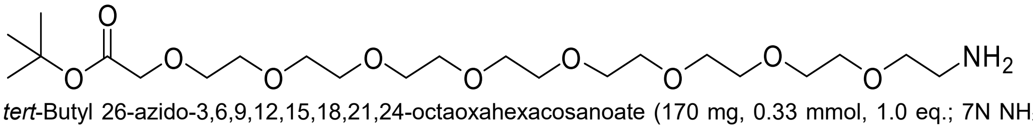

tert-Butyl 26-azido-3,6,9,12,15,18,21,24-octaoxahexacosanoate (170 mg, 0.33 mmol, 1.0 eq.; 7N NH_3 in methanol/dichloromethane (5:95); yield: 90.8 mg, 0.19 mmol, 57%.
^{1}H-NMR (600 MHz, DMSO-*d*$_6$) *δ* [ppm]: 3.98 (s, 2H, CO_2CH_2), 3.59–3.54 (m, 2H, CH_2), 3.55–3.46 (m, 28H, 14 x CH_2), 3.35 (t, $^3J_{H,H}$ = 5.81 Hz, 2H, CH_2), 2.64 (t, $^3J_{H,H}$ = 5.79 Hz, 2H, NH_2), 1.42 (s, 9H, 3 x CH_3). **^{13}C-NMR** (151 MHz, DMSO-*d*$_6$) *δ* [ppm]: 169.3 (C_q, $C_{carbonyl}$), 80.6 (C_q, *C*(CH_3)$_3$), 73.0 (CH_2), 69.8 (CH_2), 69.8 (CH_2), 69.8 (9 x CH_2), 69.7 (CH_2), 69.7 (CH_2), 69.6 (CH_2), 68.1 (CH_2), 41.3 (CH_2), 27.7 (3 x CH_3, C(CH_3)$_3$). **HRMS** (ESI-QTOF) calculated for $C_{22}H_{45}NO_{10}$ $[M+H]^+$: 484.3116; found: 484.3106. **Purity** determined by NMR: 95%.

1.10.3.2 Method B[20]

The appropriate ester (1.0 eq.) is dissolved in tetrahydrofurane (0.04 M) under an atmosphere of argon, and triphenylphosphane (2.0 eq.) is added. After stirring the solution for 24 h at RT, water (5.0 eq.) is added, and the mixture is stirred for additional 3 h. Then, the solvent is removed *in vacuo*, and the product is purified by column chromatography on silica gel using 7N NH_3 in methanol/dichloromethane as eluent. For details see below.

***tert*-Butyl 38-amino-3,6,9,12,15,18,21,24,27,30,33,36-dodecaoxaoctatriacontanoate (3d)**

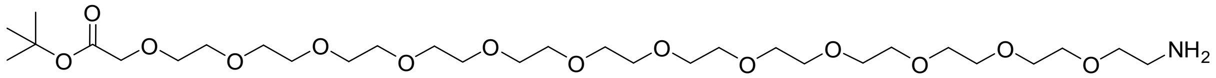

tert-Butyl 38-azido-3,6,9,12,15,18,21,24,27,30,33,36-dodecaoxaoctatriacontanoate (313 mg, 0.19 mmol, 1.0 eq.); 7N NH_3 in methanol/dichloromethane (7:93); yield: 109.1 mg, 0.17 mmol, 87%.
^{1}H-NMR (600 MHz, DMSO-*d*$_6$) *δ* [ppm]: 3.97 (s, 2H, CO_2CH_2), 3.58–3.55 (m, 2H, CH_2), 3.54–3.48 (m, 44H, 22 x CH_2), 3.35 (t, $^3J_{H,H}$ = 5.80 Hz, 2H, CH_2), 2.64 (t, $^3J_{H,H}$ = 5.77 Hz, 2H, NH_2), 1.42 (s, 9H, 3 x CH_3). **^{13}C-NMR** (151 MHz, DMSO-*d*$_6$) *δ* [ppm]: 169.4 (C_q, $C_{carbonyl}$), 80.7 (C_q, *C*(CH_3)$_3$), 72.9 (CH_2), 69.9 (CH_2), 69.8 (2 x CH_2), 69.8 (16 x CH_2), 69.8 (CH_2), 69.7 (CH_2), 69.6 (CH_2), 68.1 (CH_2), 41.3 (CH_2), 27.8 (3 x CH_3, C(CH_3)$_3$). **HRMS** (ESI-QTOF) calculated for $C_{30}H_{61}NO_{14}$ $[M+H]^+$: 660,4165; found: 660,4129. **Purity** determined by NMR: 93%.

***tert*-Butyl 50-amino-3,6,9,12,15,18,21,24,27,30,33,36,39,42,45,48-hexadecaoxapentacontanoate (3e)**

tert-Butyl 50-azido-3,6,9,12,15,18,21,24,27,30,33,36,39,42,45,48-hexadecaoxapentacontanoate (89.1 mg, 0.103 mmol, 1.0 eq.); 7N NH_3 in methanol/dichloromethane (10:90); yield: 77.7 mg, 0.093 mmol, 90%.
^{1}H-NMR (600 MHz, DMSO-*d*$_6$) *δ* [ppm]: 3.98 (s, 2H, CH_2), 3.58–3.55 (m, 2H, CH_2), 3.54–3.48 (m, 60H, 30 x CH_2), 3.36 (t, $^3J_{H,H}$ = 5.80 Hz, 2H, CH_2), 2.65 (t, $^3J_{H,H}$ = 5.77 Hz, 2H, NH_2), 1.42 (s, 9H, 3 x CH_3). **^{13}C-NMR** (151 MHz, DMSO-*d*$_6$) *δ* [ppm]: 169.3 (C_q, $C_{carbonyl}$), 80.6 (C_q, *C*(CH_3)$_3$), 72.9 (CH_2), 69.8 (CH_2), 69.8 (26 x CH_2), 69.7 (CH_2), 69.7 (CH_2), 69.6 (CH_2), 68.1 (CH_2), 41.3 (CH_2), 27.7 (3 x CH_3, C(CH_3)$_3$). **HRMS** (ESI-QTOF) calculated for $C_{38}H_{77}NO_{18}$ $[M+H]^+$: 836.5213; found: 836.5193. **Purity** determined by NMR: 95%.

***tert*-Butyl 62-amino-3,6,9,12,15,18,21,24,27,30,33,36,39,42,45,48,51,54,57,60-icosaoxadohexacontanoate (3f)**

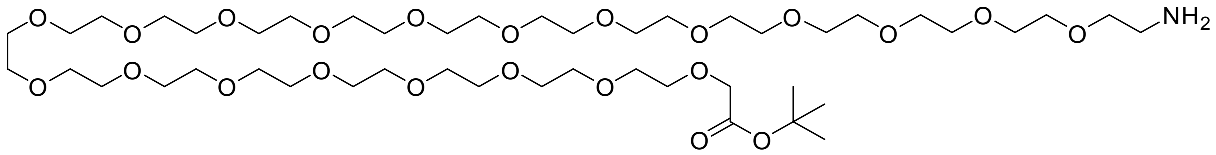

tert-Butyl 62-azido-3,6,9,12,15,18,21,24,27,30,33,36,39,42,45,48,51,54,57,60-icosaoxadohexacontanoate (200 mg, 0.19 mmol, 1.0 eq.); 7N NH_3 in methanol/dichloromethane (8:92); yield: 140.2 mg, 0.14 mmol, 71%.
^{1}H-NMR (600 MHz, DMSO-*d*$_6$) *δ* [ppm]: 3.98 (s, 2H, CH_2), 3.58–3.55 (m, 2H, CH_2), 3.55–3.48 (m, 76H, 38 x CH_2), 3.36 (t, $^3J_{H,H}$ = 5.79 Hz, 2H, CH_2), 2.65 (t, $^3J_{H,H}$ = 5.79 Hz, 2H, NH_2), 1.42 (s, 9H, 3 x CH_3). **^{13}C-NMR** (151 MHz, DMSO-*d*$_6$) *δ* [ppm]: 169.3 (C_q, $C_{carbonyl}$), 80.6 (C_q, *C*(CH_3)$_3$), 72.9 (CH_2), 69.8 (CH_2), 69.8 (2 x CH_2), 69.8 (32 x CH_2), 69.7 (CH_2), 69.7 (CH_2), 69.6 (CH_2), 68.1 (CH_2), 41.3 (CH_2), 27.7 (3 x CH_3, C(CH_3)$_3$). **HRMS** (ESI-QTOF) calculated for $C_{46}H_{93}NO_{22}$ $[M+H]^+$: 1012.6262; found: 1012.6239. **Purity** determined by NMR: 93%.

2. Supplementary Figures

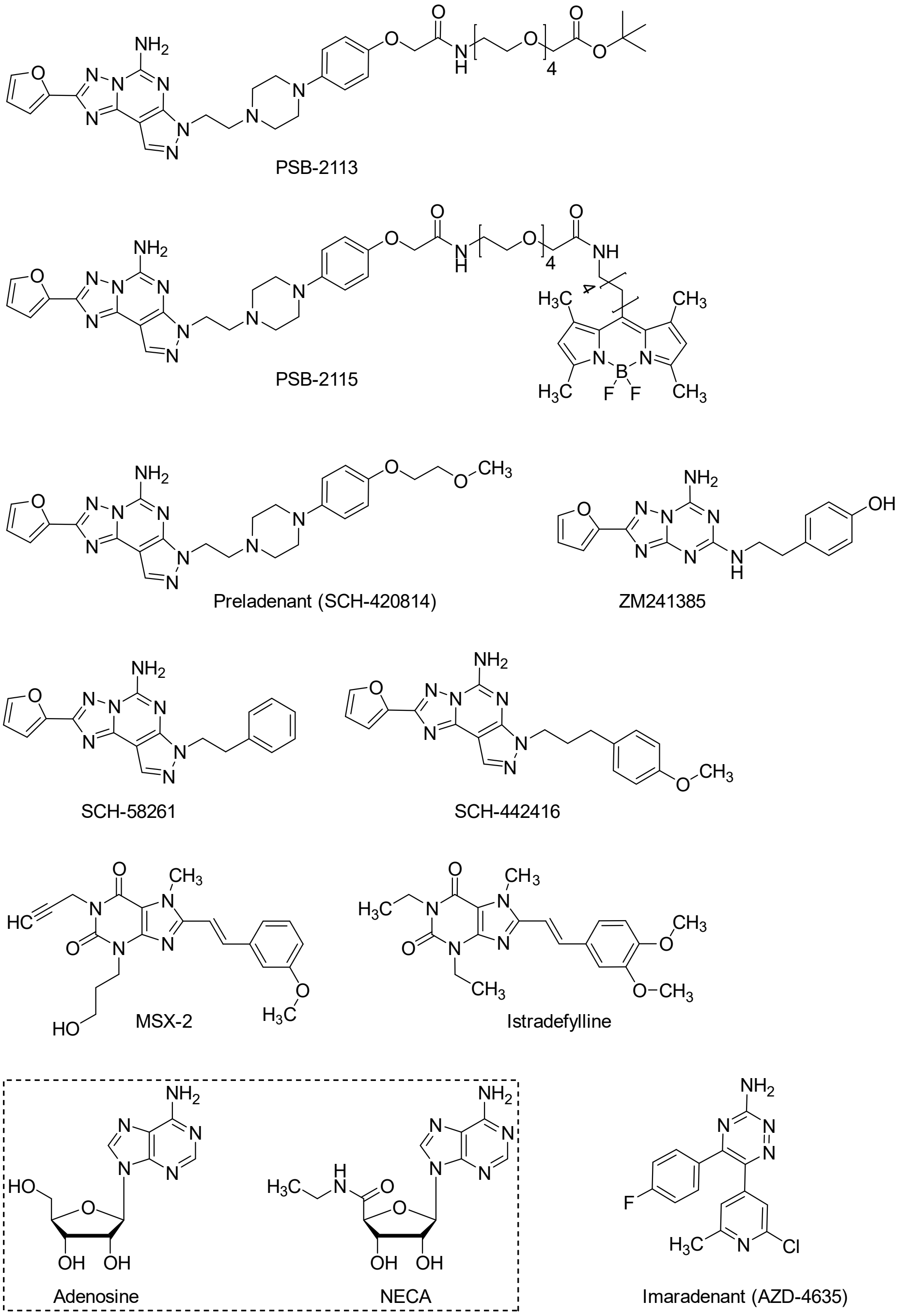

Figure S1. Chemical structures of discussed $A_{2A}AR$ antagonists and agonists. The two agonists adenosine and NECA are highlighted by a dashed rectangle.

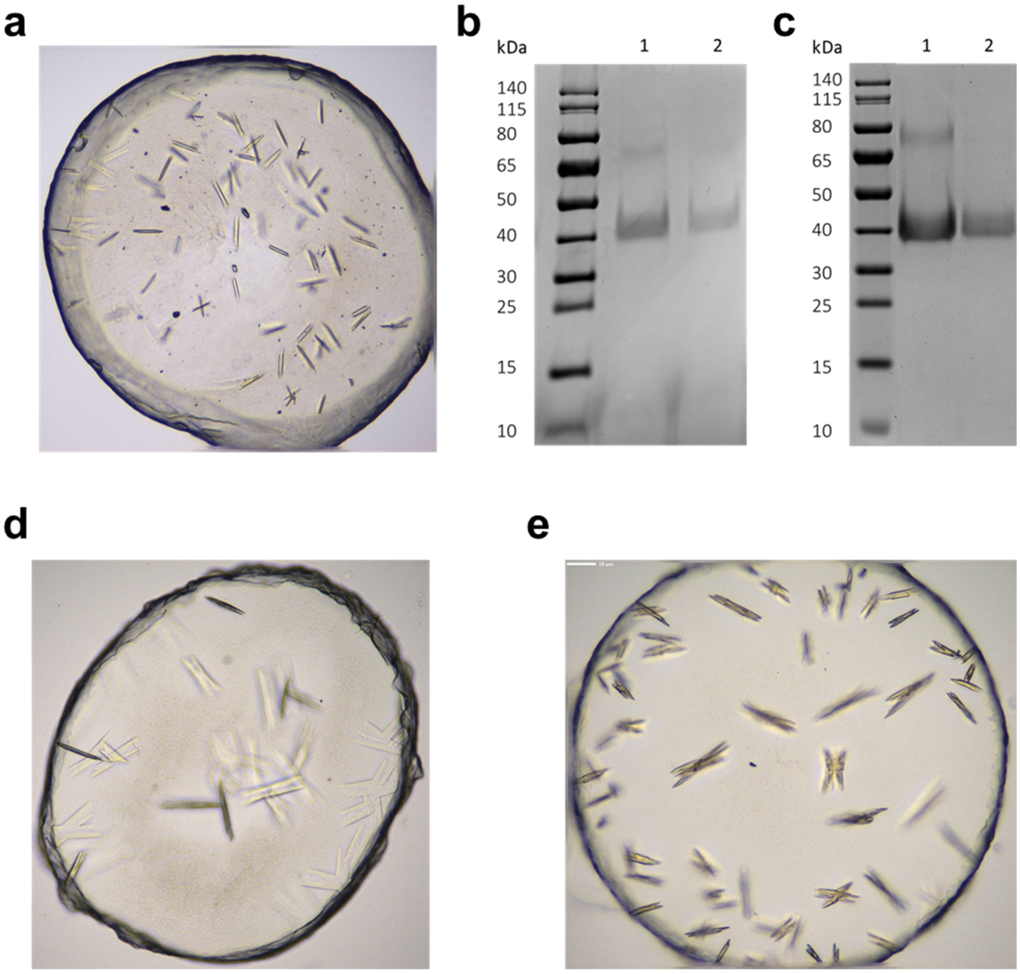

Figure S2. Crystallization and SDS-PAGE results**. a** Non-diffracting crystals of A_{2A}-ΔC-bRIL purified in complex with PSB-2113. **b** SDS-PAGE results of the unconcentrated A_{2A}-PSB1-bRIL-PSB-2113 complex, either (1) undiluted or (2) diluted with water in a 1 to 4 ratio. **c** SDS-PAGE results of the unconcentrated A_{2A}-PSB1-bRIL-PSB-2115 complex, either (1) undiluted or (2) diluted with water in a 1 to 4 ratio. **d** Crystals of the A_{2A}-PSB1-bRIL-PSB-2113 complex in LCP. **e** Crystals of the A_{2A}-PSB1-bRIL-PSB-2115 complex in LCP.

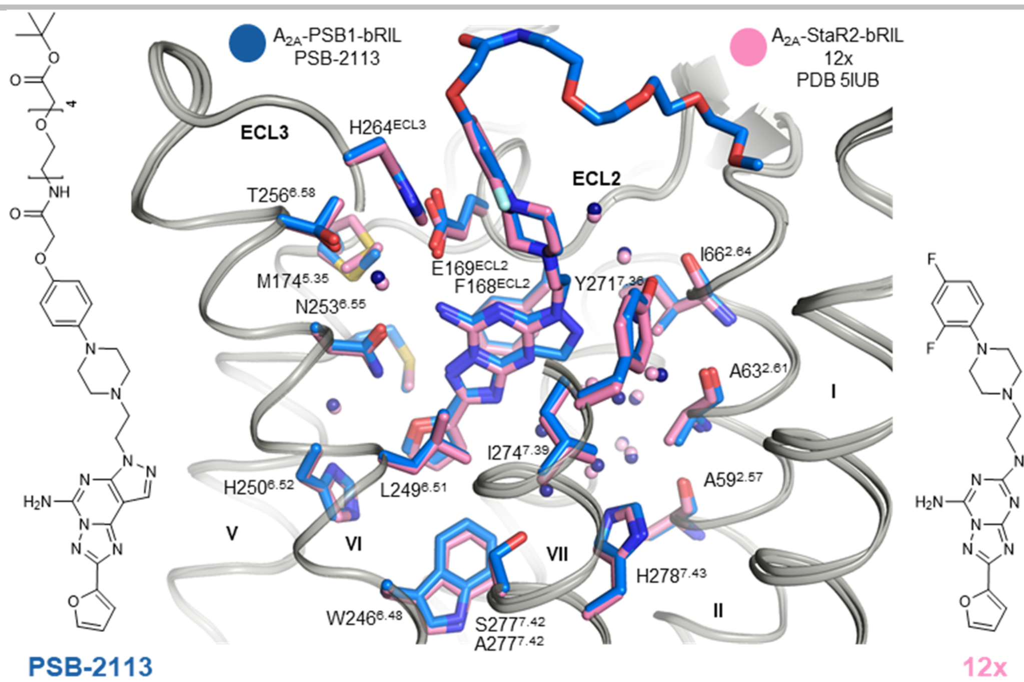

Figure S3. Binding pocket comparison of the new A_{2A}-PSB1-bRIl-PSB-2113 structure (blue) with the structure of A_{2A}-StaR2-bRIL in complex with A_{2A}AR antagonist **12x** (pink, PDB 5UIB).[6] 12x comprises a comparable phenylpiperazinylethyl extension as PSB-2113 and forms similar π-π interactions to $H264^{ECL3}$.

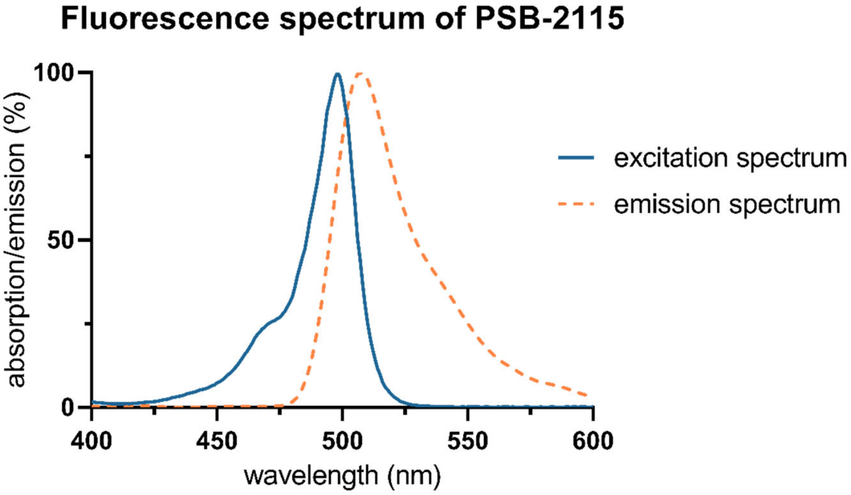

Figure S4. Fluorescence spectrum of BODIPY-conjugated preladenant derivative PSB-2115.

3. Supplementary Tables

Table S1. Complete list of all published A_{2A}AR crystal structures in complex with A_{2A}AR antagonists in the Protein Data Bank as of January 2022.

PDB Identifier	Ligand	A_{2A}AR construct	Resolution (Å)	H264-E169 ionic lock	pH during crystallization	Reference
3EML	ZM241385	A_{2A}-ΔC-T4L	2.60	intact	5.5 – 6.5	[21]
4EIY 5K2A 5K2B 5K2C 5K2D 5UVI 5JTB 5VRA 6AQF 6MH8 6JZH 6PS7 6WQA 6LPJ 6LPK 6LPL 7RM5	ZM241385	A_{2A}-ΔC-bRIL (+ N154Q in 6AQF)	1.80 – 4.20	intact	4.8 – 5.0	[3,22]
3PWH	ZM241385	A_{2A}-StaR2	3.30	open	8.0 – 8.75	[23]
3VGA 3VG9	ZM241385	A_{2A}-ΔC + N154Q	2.70 – 3.10	intact/open	6.5	[24]
5IU4 5NLX 5NM2 5NM4 5OLG 6S0L 6S0Q	ZM241385	A_{2A}-StaR2-bRIL	1.72 – 2.14	intact	5.0 – 5.4	[6,25,26]
3REY	Xanthine amine congener (XAC)	A_{2A}-StaR2	3.31	open	8.0 – 8.75	[23]
3RFM	Caffeine	A_{2A}-StaR2	3.60	open	8.0 – 8.75	[23]
5MZP	Caffeine	A_{2A}-StaR2-bRIL	2.10	intact	5.0	[27]
3UZA	4g	A_{2A}-StaR2	3.27	open	8.0 – 8.75	[28]
3UZC	4e	A_{2A}-StaR2	3.34	open	8.0 – 8.75	[28]
5OLZ	4e	A_{2A}-StaR2-bRIL	1.90	intact	5.3 – 5.4	[26]
5OM1	4e	A_{2A}-StaR2-bRIL	2.10	intact	5.3 – 5.4	[26]
5OM4	4e	A_{2A}-StaR2-bRIL	2.00	intact	5.3 – 5.4	[26]
5IU7	12c	A_{2A}-StaR2-bRIL	1.90	intact	5.3 – 5.4	[6]
5IU8	12f	A_{2A}-StaR2-bRIL	2.00	intact	5.5	[6]
5IUA	12b	A_{2A}-StaR2-bRIL	2.20	intact	5.3 – 5.4	[6]
5IUB	12x	A_{2A}-StaR2-bRIL	2.10	intact	5.5	[6]
5MZJ	Theophylline	A_{2A}-StaR2-bRIL	2.00	intact	5.1	[27]
5N2R	PSB-36	A_{2A}-StaR2-bRIL	2.80	open	5.1	[27]
5OLH	Vipadenant	A_{2A}-StaR2-bRIL	2.60	intact	5.3 – 5.4	[26]
5OLO	Tozadenant	A_{2A}-StaR2-bRIL	3.10	open	5.3 – 5.4	[26]
5OLV	LUAA47070	A_{2A}-StaR2-bRIL	2.00	intact	5.3 – 5.4	[26]
5UIG	Cmpd-1	A_{2A}-ΔC-bRIL (modified N- and C- terminus	3.50	intact	6.5	[29]
6GT3	Imaradenant	A_{2A}-StaR2-bRIL	2.00	intact	5.3 – 5.4	[30]
6ZDR	Chromone 4d	A_{2A}-StaR2-bRIL	1.92	intact	4.7 – 5.4	[31]
6ZDV	Chromone 5d	A_{2A}-StaR2-bRIL	2.13	intact	4.7 – 5.4	[31]

Table S2. Data collection and refinement statistics. Statistics for the highest resolution shell are shown in parentheses.

	Data collection	
	**A_{2A}-PSB1-bRIL-PSB-2113 (PDB 7PX4)**	**A_{2A}-PSB1-bRIL-PSB-2115 (PDB 7PYR)**
**Number of crystals used**	1	1
**Space group**	$C\ 2\ 2\ 2_1$	$C\ 2\ 2\ 2_1$
**Cell parameters a, b, c (Å)**	39.6, 180.262, 139.483	39.31, 180.11, 140.4
**Number of reflections processed**	158,598 (15,265)	103,499 (10,564)
**Number of unique reflections**	24,297 (2,375)	15,875 (1,545)
**Resolution (Å)**	41.32 – 2.25 (2.33 – 2.25)	45.03 - 2.6 (2.69 – 2.6)
**R_{merge} (%)**	21.30 (134.5)	19.53 (133.8)
**$CC_{1/2}$**	0.995 (0.574)	0.995 (0.585)
**Mean I/σ (I)**	7.69 (1.36)	9.26 (2.42)
**Completeness (%)**	99.70 (98.02)	97.92 (94.70)
**Redundancy**	6.5 (6.4)	6.5 (6.8)
	**Refinement**	
**Resolution (Å)**	2.25	2.6
**Number of reflections (test set)**	24,282 (2,372)	15,569 (1,464)
**R_{work} / R_{free} (%)**	19.55 / 23.94 (29.50 / 37.11)	19.15 / 25.04 (23.36 / 28.84)
	**Number of atoms**	
**A_{2A}AR**	2,308	2,312
**bRIL**	696	693
**Ligand**	50	50
**Lipids, PEG and waters**	759	721
	**Overall *B* values (Å^2)**	
**A_{2A}AR**	35.60	40.16
**bRIL**	70.17	82.48
**Ligand**	44.00	49.45
**Lipids, PEG and waters**	55.48	61.59
	**RMSD**	
**Bond lengths (Å)**	0.002	0.003
**Bond angles (°)**	0.42	0.49
	**Ramachandran plot statistics**	
**Favored regions (%)**	98.45	98.70
**Allowed regions (%)**	1.55	1.30
**Disallowed regions (%)**	0	0

Table S3. Affinities determined in radioligand binding assays and potencies determined in the TRUPATH G protein activation assay.[a]

A_{2A}AR construct	MSX-2<br>vs. [^{3}H]MSX-2<br>K_d ± SEM (nM)	PSB-2113<br>vs. [^{3}H]MSX-2<br>K_i ± SEM (nM)	NECA<br>vs. [^{3}H]MSX-2<br>K_i ± SEM (nM)	NECA<br>Gα$_{s-short}$RLuc8-Gβ$_3$-Gγ$_9$GFP2<br>EC_{50} ± SEM (nM)
**A_{2A} wt**	**13.3** ± 0.756	**6.30** ± 0.792	**603** ± 141	**58.6** ± 28.6
**A_{2A}-ΔC**	**17.7** ± 2.02	**12.7** ± 3.30	**230** ± 39.8	**6.86** ± 3.50
**A_{2A}-ΔC-bRIL**	**15.1** ± 2.09	**12.0** ± 2.31	**494** ± 296	**no activation**
**A_{2A}-PSB1-bRIL**	**20.4** ± 3.54	**19.6** ± 3.60	**no binding**	**no activation**
**A_{2A}-PSB1**	N.D.	N.D.	N.D.	**no activation**
**A_{2A}-StaR2-bRIL**	**17.1** ± 0.240	N.D.	**no binding**	N.D.

[a]K_d and K_i values are means ± SEM from three independent experiments. EC_{50} values are means ± SEM from four to five independent experiments. N.D. not determined.

4. References

[1] F. Heisig, S. Gollos, S. J. Freudenthal, A. El-Tayeb, J. Iqbal, C. E. Müller, *J. Fluoresc.* **2014**, *24*, 213–230.
[2] D. B. Garrity, M. J. Chang, G. W. Blissard, *Virology* **1997**, *231*, 167–181.
[3] W. Liu, E. Chun, A. A. Thompson, P. Chubukov, F. Xu, V. Katritch, G. W. Han, C. B. Roth, L. H. Heitman, A. P. IJzerman et al., *Science* **2012**, *337*, 232–236.
[4] X. M. Guan, T. S. Kobilka, B. K. Kobilka, *J. Biol. Chem.* **1992**, *267*, 21995–21998.
[5] E. Chun, A. A. Thompson, W. Liu, C. B. Roth, M. T. Griffith, V. Katritch, J. Kunken, F. Xu, V. Cherezov, M. A. Hanson et al., *Structure* **2012**, *20*, 967–976.
[6] E. Segala, D. Guo, R. K. Y. Cheng, A. Bortolato, F. Deflorian, A. S. Doré, J. C. Errey, L. H. Heitman, A. P. IJzerman, F. H. Marshall et al., *J. Med. Chem.* **2016**, *59*, 6470–6479.
[7] M. Audet, K. Villers, J. Velasquez, M. Chu, C. Hanson, R. C. Stevens, *Nat. Protoc.* **2020**, *15*, 144–160.
[8] T. Claff, J. Yu, V. Blais, N. Patel, C. Martin, L. Wu, G. W. Han, B. J. Holleran, O. van der Poorten, K. L. White et al., *Sci. Adv.* **2019**, *5*, eaax9115.
[9] M. Caffrey, V. Cherezov, *Nat. Protoc.* **2009**, *4*, 706–731.
[10] W. Kabsch, *Acta Crystallogr. D Biol. Crystallogr.* **2010**, *66*, 125–132.
[11] A. J. McCoy, R. W. Grosse-Kunstleve, P. D. Adams, M. D. Winn, L. C. Storoni, R. J. Read, *J. Appl. Crystallogr.* **2007**, *40*, 658–674.
[12] T. C. Terwilliger, R. W. Grosse-Kunstleve, P. V. Afonine, N. W. Moriarty, P. H. Zwart, L. W. Hung, R. J. Read, P. D. Adams, *Acta Crystallogr. D Biol. Crystallogr.* **2008**, *64*, 61–69.
[13] P. Emsley, B. Lohkamp, W. G. Scott, K. Cowtan, *Acta Crystallogr. D Biol. Crystallogr.* **2010**, *66*, 486–501.
[14] M. M. Bradford, *Anal. Biochem.* **1976**, *72*, 248–254.
[15] C. E. Müller, R. Sauer, Y. Maurinsh, R. Huertas, F. Flle, K.-N. Klotz, J. Nagel, W. Hauber, *Drug Dev. Res.* **1998**, *45*, 190–197.
[16] R. H. J. Olsen, J. F. DiBerto, J. G. English, A. M. Glaudin, B. E. Krumm, S. T. Slocum, T. Che, A. C. Gavin, J. D. McCorvy, B. L. Roth et al., *Nat. Chem. Biol.* **2020**, *16*, 841–849.
[17] P. Baraldi, *Bioorg. Med. Chem.* **2003**, *11*, 4161–4169.
[18] B. R. Neustadt, N. A. Lindo, W. J. Greenlee, D. Tulshian, L. S. Silverman, Y. Xia, C. D. Boyle, S. Chackala-Mannil, *International Patent* **2001**, *WO 01/92264 A1*.
[19] H. Zhang, X. Li, Q. Shi, Y. Li, G. Xia, L. Chen, Z. Yang, Z.-X. Jiang, *Angew. Chem. Int. Ed.* **2015**, *127*, 3834–3838.
[20] Z. Wan, Y. Li, S. Bo, M. Gao, X. Wang, K. Zeng, X. Tao, X. Li, Z. Yang, Z.-X. Jiang, *Org. Biomol. Chem.* **2016**, *14*, 7912–7919.
[21] V.-P. Jaakola, M. T. Griffith, M. A. Hanson, V. Cherezov, E. Y. T. Chien, J. R. Lane, A. P. IJzerman, R. C. Stevens, *Science* **2008**, *322*, 1211–1217.
[22] a) M. W. Martynowycz, A. Shiriaeva, X. Ge, J. Hattne, B. L. Nannenga, V. Cherezov, T. Gonen, *PNAS* **2021**, *118*; b) K. Ihara, M. Hato, T. Nakane, K. Yamashita, T. Kimura-Someya, T. Hosaka, Y. Ishizuka-Katsura, R. Tanaka, T. Tanaka, M. Sugahara et al., *Sci. Rep.* **2020**, *10*, 19305; c) M.-Y. Lee, J. Geiger, A. Ishchenko, G. W. Han, A. Barty, T. A. White, C. Gati, A. Batyuk, M. S. Hunter, A. Aquila et al., *IUCrJ* **2020**, *7*, 976–984; d) A. Ishchenko, B. Stauch, G. W. Han, A. Batyuk, A. Shiriaeva, C. Li, N. Zatsepin, U. Weierstall, W. Liu, E. Nango et al., *IUCrJ* **2019**, *6*, 1106–1119; e) Y. Shimazu, K. Tono, T. Tanaka, Y. Yamanaka, T. Nakane, C. Mori, K. Terakado Kimura, T. Fujiwara, M. Sugahara, R. Tanaka et al., *J. Appl. Crystallogr.* **2019**, *52*, 1280–1288; f) J. M. Martin-Garcia, L. Zhu, D. Mendez, M.-Y. Lee, E. Chun, C. Li, H. Hu, G. Subramanian, D. Kissick, C. Ogata et al., *IUCrJ* **2019**, *6*, 412–425; g) K. L. White, M. T. Eddy, Z.-G. Gao, G. W. Han, T. Lian, A. Deary, N. Patel, K. A. Jacobson, V. Katritch, R. C. Stevens, *Structure* **2018**, *26*, 259-269.e5; h) J. Broecker, T. Morizumi, W.-L. Ou, V. Klingel, A. Kuo, D. J. Kissick, A. Ishchenko, M.-Y. Lee, S. Xu, O. Makarov et al., *Nat. Protoc.* **2018**, *13*, 260–292; i) I. Melnikov, V. Polovinkin, K. Kovalev, I. Gushchin, M. Shevtsov, V. Shevchenko, A. Mishin, A. Alekseev, F. Rodriguez-Valera, V. Borshchevskiy et al., *Sci. Adv.* **2017**, *3*, e1602952; j) J.

M. Martin-Garcia, C. E. Conrad, G. Nelson, N. Stander, N. A. Zatsepin, J. Zook, L. Zhu, J. Geiger, E. Chun, D. Kissick et al., *IUCrJ* **2017**, *4*, 439–454; k) A. Batyuk, L. Galli, A. Ishchenko, G. W. Han, C. Gati, P. A. Popov, M.-Y. Lee, B. Stauch, T. A. White, A. Barty et al., *Sci. Adv.* **2016**, *2*, e1600292.

[23] A. S. Doré, N. Robertson, J. C. Errey, I. Ng, K. Hollenstein, B. Tehan, E. Hurrell, K. Bennett, M. Congreve, F. Magnani et al., *Structure* **2011**, *19*, 1283–1293.

[24] T. Hino, T. Arakawa, H. Iwanari, T. Yurugi-Kobayashi, C. Ikeda-Suno, Y. Nakada-Nakura, O. Kusano-Arai, S. Weyand, T. Shimamura, N. Nomura et al., *Nature* **2012**, *482*, 237–240.

[25] T. Weinert, N. Olieric, R. Cheng, S. Brünle, D. James, D. Ozerov, D. Gashi, L. Vera, M. Marsh, K. Jaeger et al., *Nat. Commun.* **2017**, *8*, 542.

[26] P. Rucktooa, R. K. Y. Cheng, E. Segala, T. Geng, J. C. Errey, G. A. Brown, R. M. Cooke, F. H. Marshall, A. S. Doré, *Sci. Rep.* **2018**, *8*, 41.

[27] R. K. Y. Cheng, E. Segala, N. Robertson, F. Deflorian, A. S. Doré, J. C. Errey, C. Fiez-Vandal, F. H. Marshall, R. M. Cooke, *Structure* **2017**, *25*, 1275-1285.e4.

[28] M. Congreve, S. P. Andrews, A. S. Doré, K. Hollenstein, E. Hurrell, C. J. Langmead, J. S. Mason, I. W. Ng, B. Tehan, A. Zhukov et al., *J. Med. Chem.* **2012**, *55*, 1898–1903.

[29] B. Sun, P. Bachhawat, M. L.-H. Chu, M. Wood, T. Ceska, Z. A. Sands, J. Mercier, F. Lebon, T. S. Kobilka, B. K. Kobilka, *PNAS* **2017**, *114*, 2066–2071.

[30] A. Borodovsky, C. M. Barbon, Y. Wang, M. Ye, L. Prickett, D. Chandra, J. Shaw, N. Deng, K. Sachsenmeier, J. D. Clarke et al., *J. ImmunoTher. Cancer* **2020**, *8*, e000417.

[31] W. Jespers, G. Verdon, J. Azuaje, M. Majellaro, H. Keränen, X. García-Mera, M. Congreve, F. Deflorian, C. de Graaf, A. Zhukov et al., *Angew. Chem. Int. Ed.* **2020**, *59*, 16536–16543.

5. Author Contributions

T.C. designed and performed expression, purification and crystallization experiments and analyzed the data; T.A.K. designed, synthesized and analyzed preladenant derivatives; U.K.T.S. collected and analyzed X-ray diffraction data and solved the crystal structures; V.J.V. and J.G.S. helped with protein expression, purification, crystallization experiments and data analysis; C.V. performed radioligand binding experiments; J.H.V. performed TRUPATH experiments; J.L. supervised X-ray data collection and structural refinement; C.E.M. initiated the project, acquired funding and supervised the project. T.C. and C.E.M. wrote the manuscript with contributions from all co-authors.

6. ^{1}H and ^{13}C NMR Spectra

^{1}H-NMR *tert*-Butyl-2-(4-(4-(2-(5-amino-2-furan-2-yl)-7H-pyrazolo[4,3-*e*][1,2,4]triazolo[1,5-*c*]pyrimidin-7-yl)ethyl)piperazin)-1-yl)phenoxy)acetat in DMSO-d_6

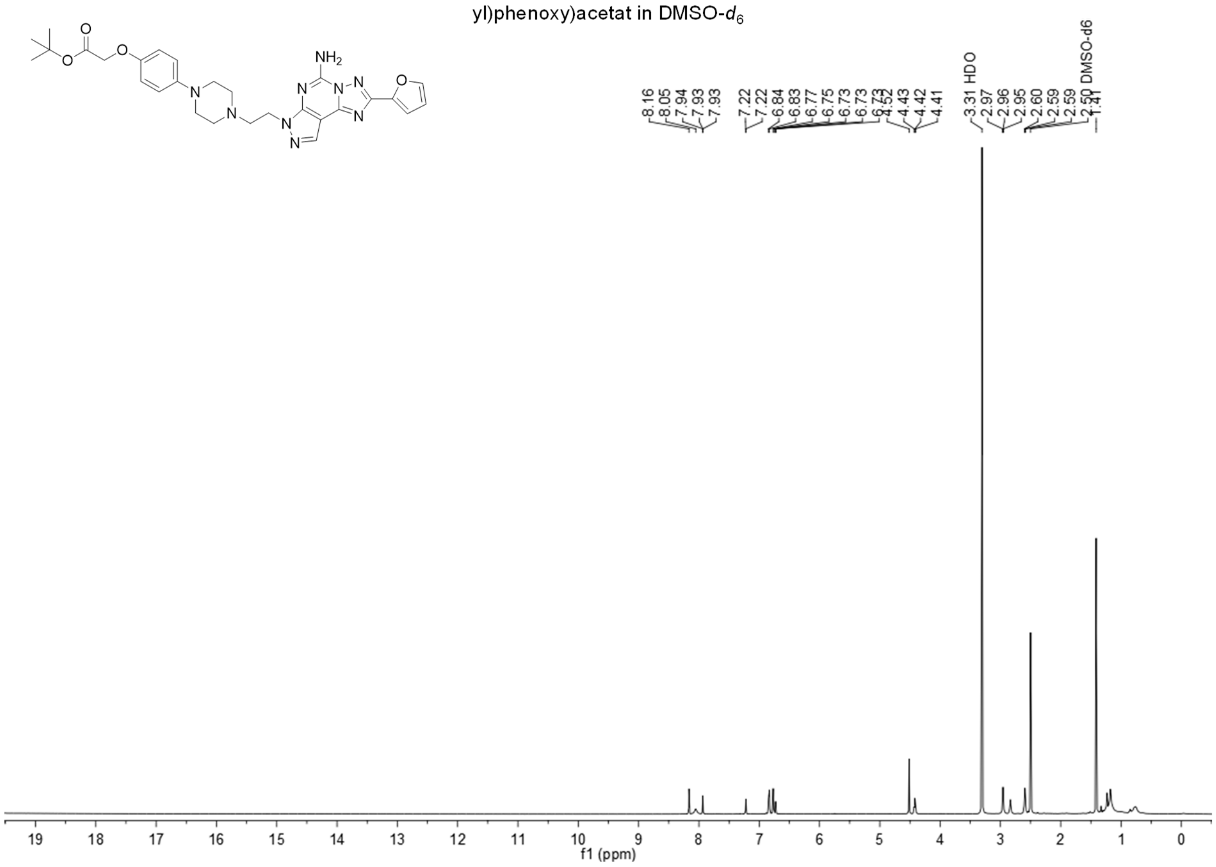

^{13}C-NMR *tert*-Butyl-2-(4-(4-(2-(5-amino-2-furan-2-yl)-7H-pyrazolo[4,3-*e*][1,2,4]triazolo[1,5-*c*]pyrimidin-7-yl)ethyl)piperazin)-1-yl)phenoxy)acetat in DMSO-d_6

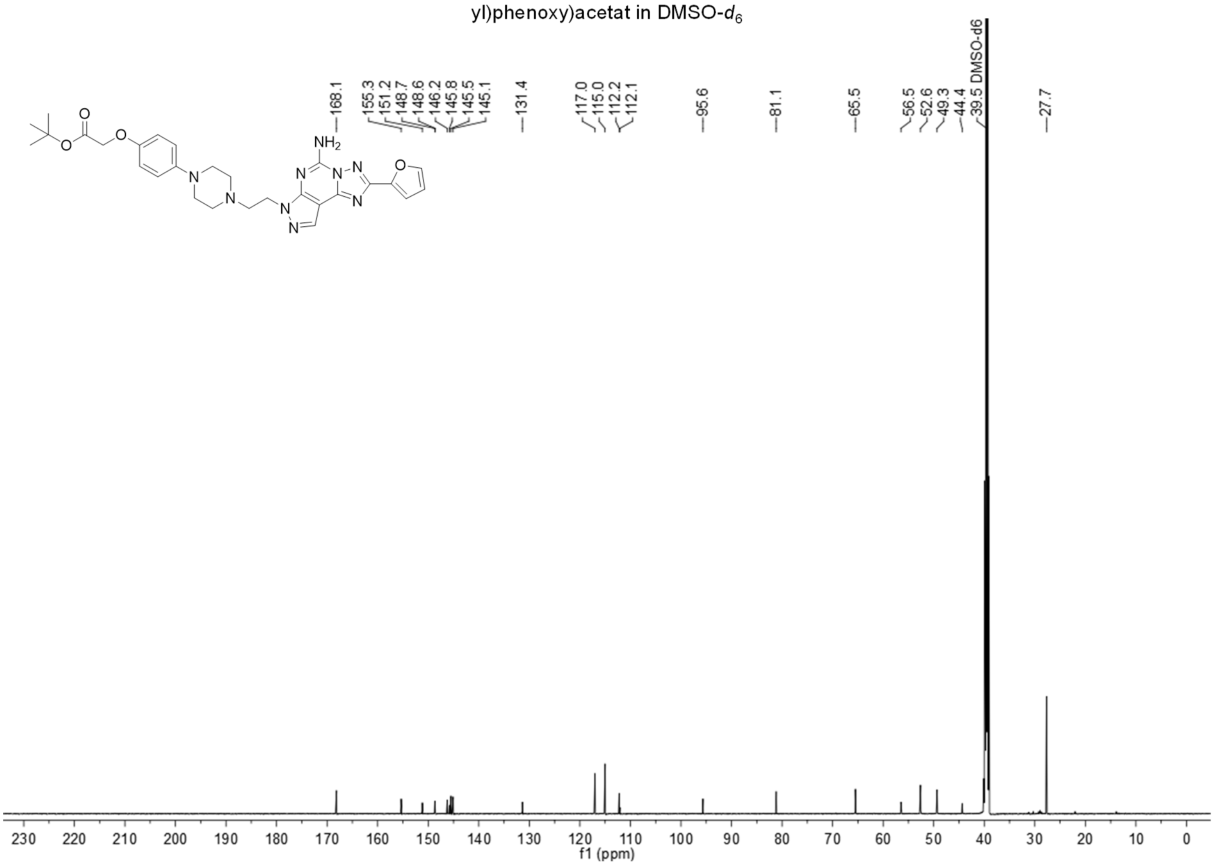

^{1}H-NMR 2-(4-(4-(2-(5-Amino-2-(furan-2-yl)-7*H*-pyrazolo[4,3-*e*][1,2,4]triazolo[1,5-*c*]pyrimidin-7-yl)ethyl)piperazin-1-yl)phenoxy)essigsäure in DMSO-d_6

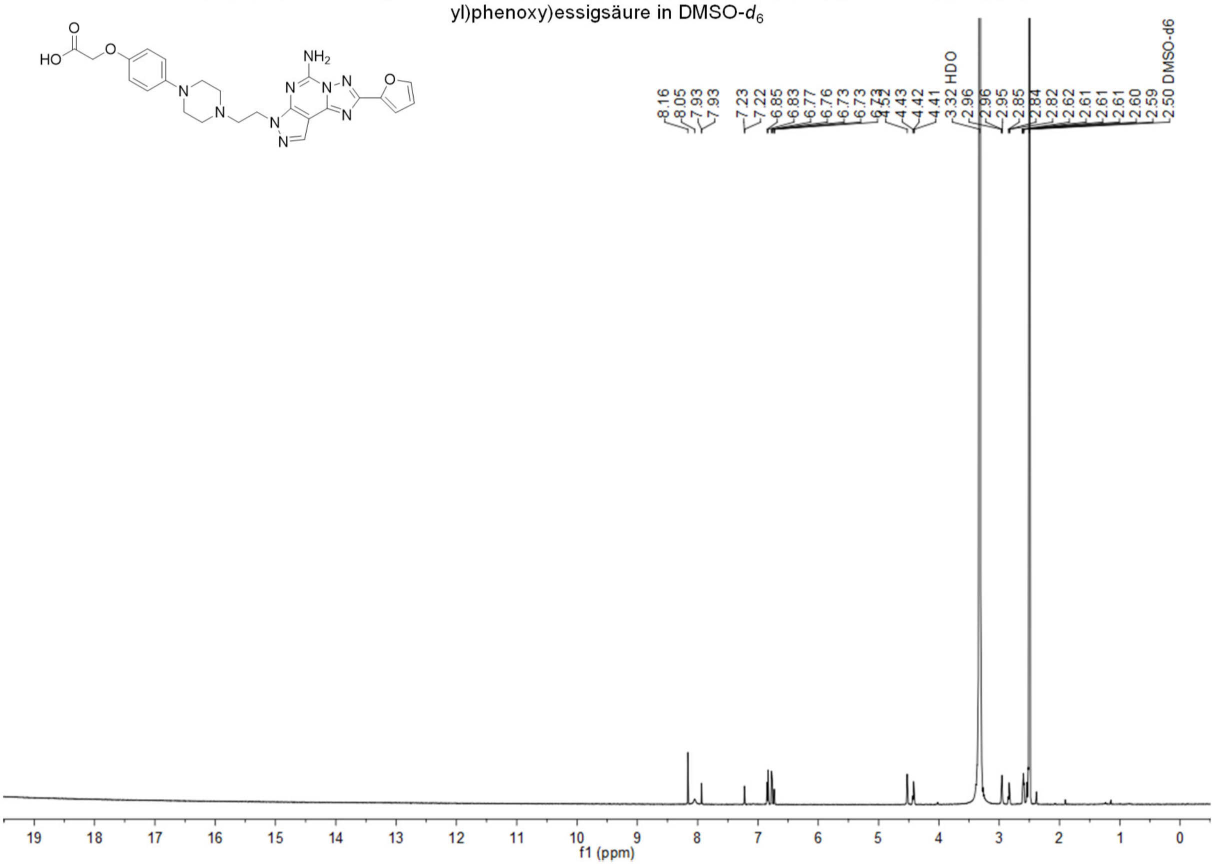

^{13}C-NMR 2-(4-(4-(2-(5-Amino-2-(furan-2-yl)-7*H*-pyrazolo[4,3-*e*][1,2,4]triazolo[1,5-*c*]pyrimidin-7-yl)ethyl)piperazin-1-yl)phenoxy)essigsäure in DMSO-d_6

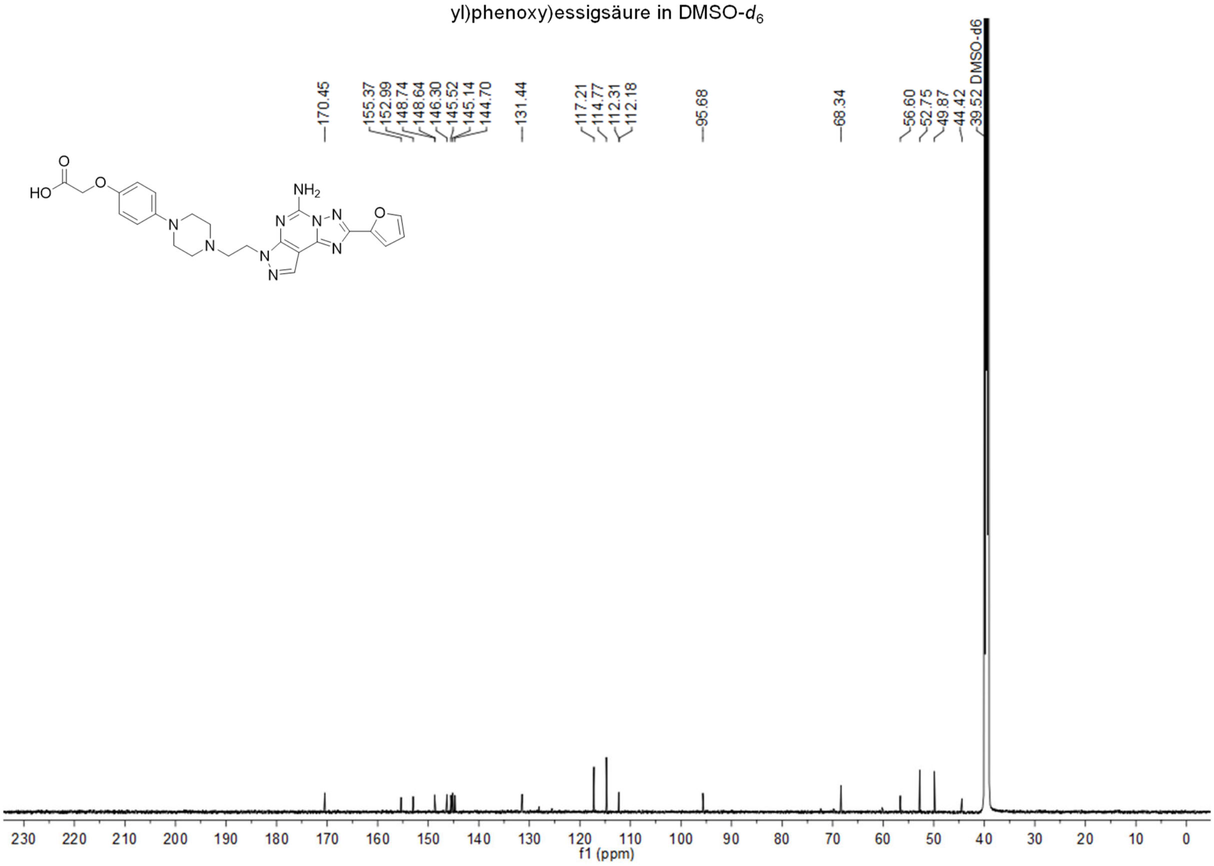

^{1}H-NMR *tert*-Butyl-1-(4-(4-(2-(5-amino-2-(furan-2-yl)-7*H*-pyrazolo[4,3-*e*][1,2,4]triazolo[1,5-*c*]pyrimidin-7-yl)ethyl)piperazin-1-yl)phenoxy)-2-oxo-6,9,12,15-tetraoxa-3-azaheptadecan-17-oat in DMSO-d_6

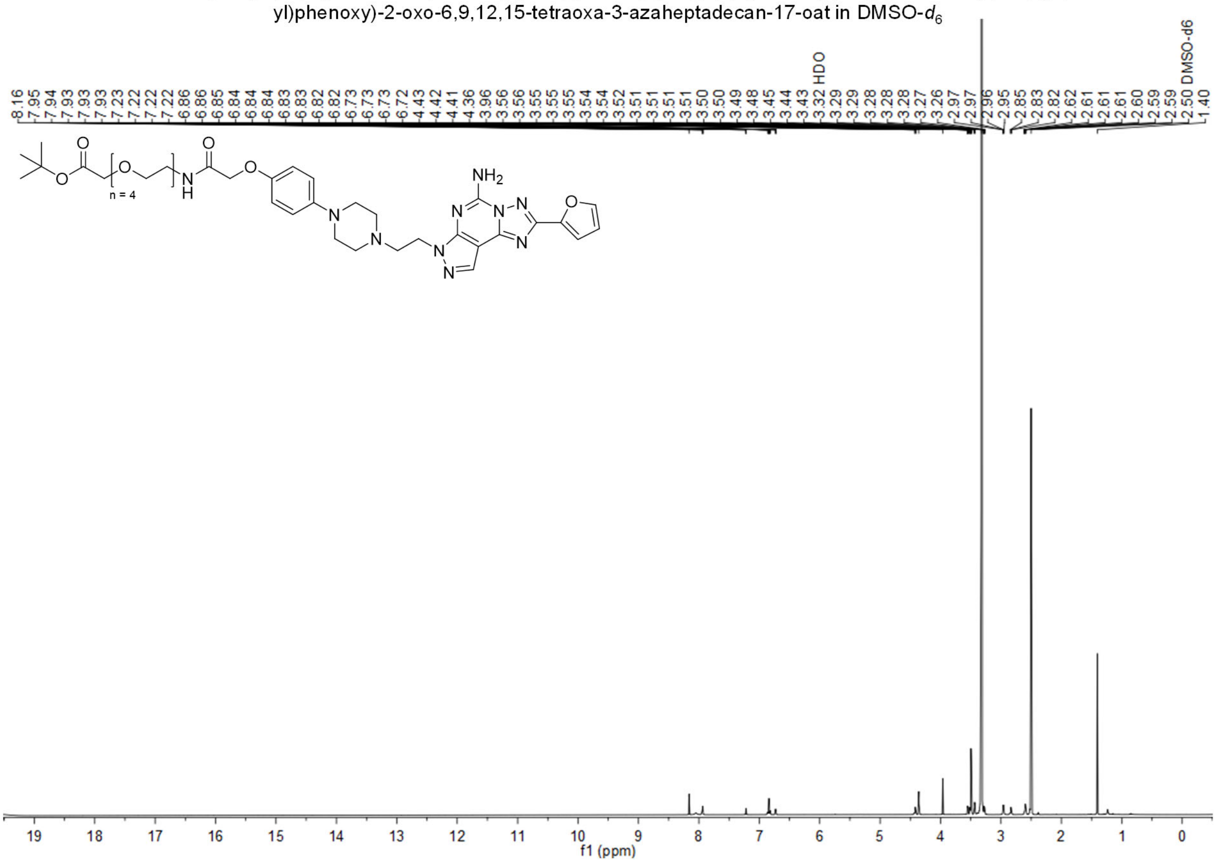

^{13}C-NMR *tert*-Butyl-1-(4-(4-(2-(5-amino-2-(furan-2-yl)-7*H*-pyrazolo[4,3-*e*][1,2,4]triazolo[1,5-*c*]pyrimidin-7-yl)ethyl)piperazin-1-yl)phenoxy)-2-oxo-6,9,12,15-tetraoxa-3-azaheptadecan-17-oat in DMSO-d_6

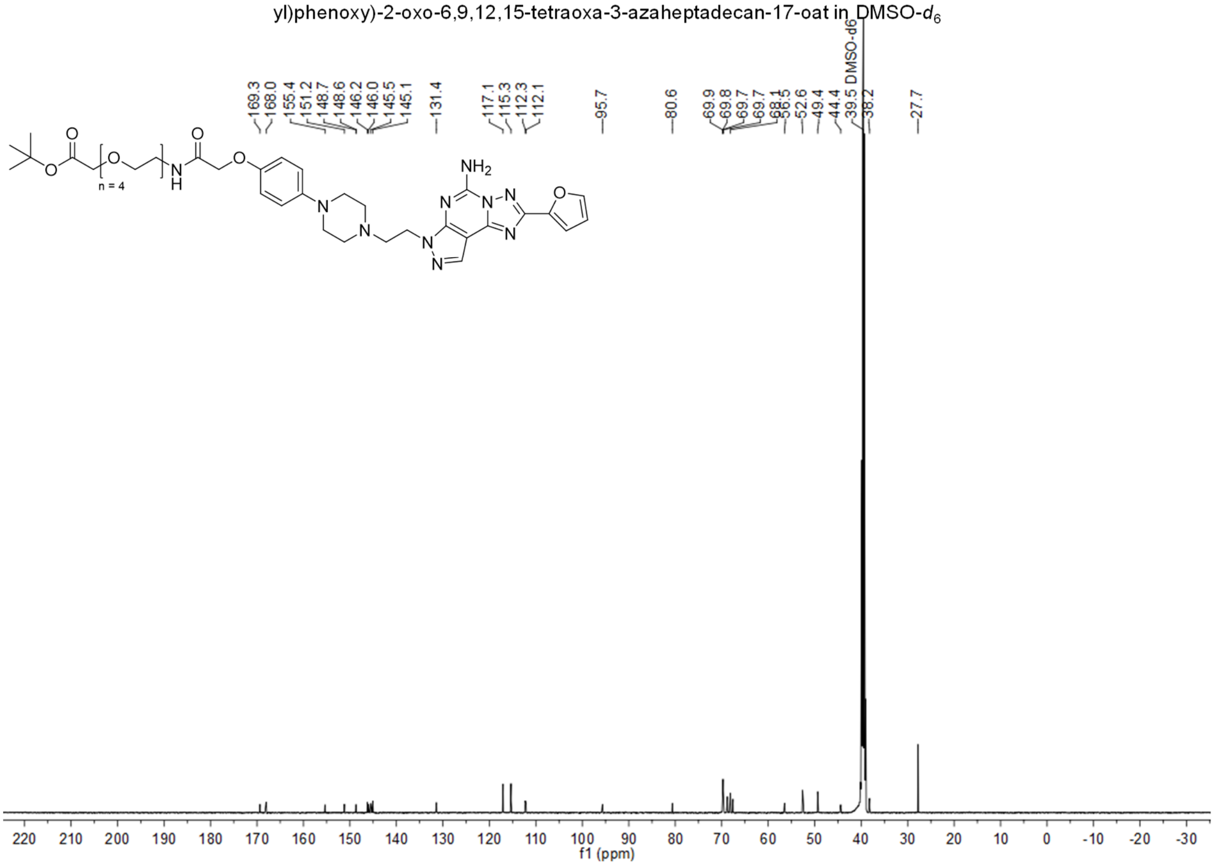

^{1}H-NMR *tert*-Butyl-1-(4-(4-(2-(5-amino-2-(furan-2-yl)-7*H*-pyrazolo[4,3-*e*][1,2,4]triazolo[1,5-*c*]pyrimidin-7-yl)ethyl)piperazin-1-yl)phenoxy)-2-oxo-6,9,12,15,18-pentaoxa-3-azaicosan-20-oat in DMSO-d_6

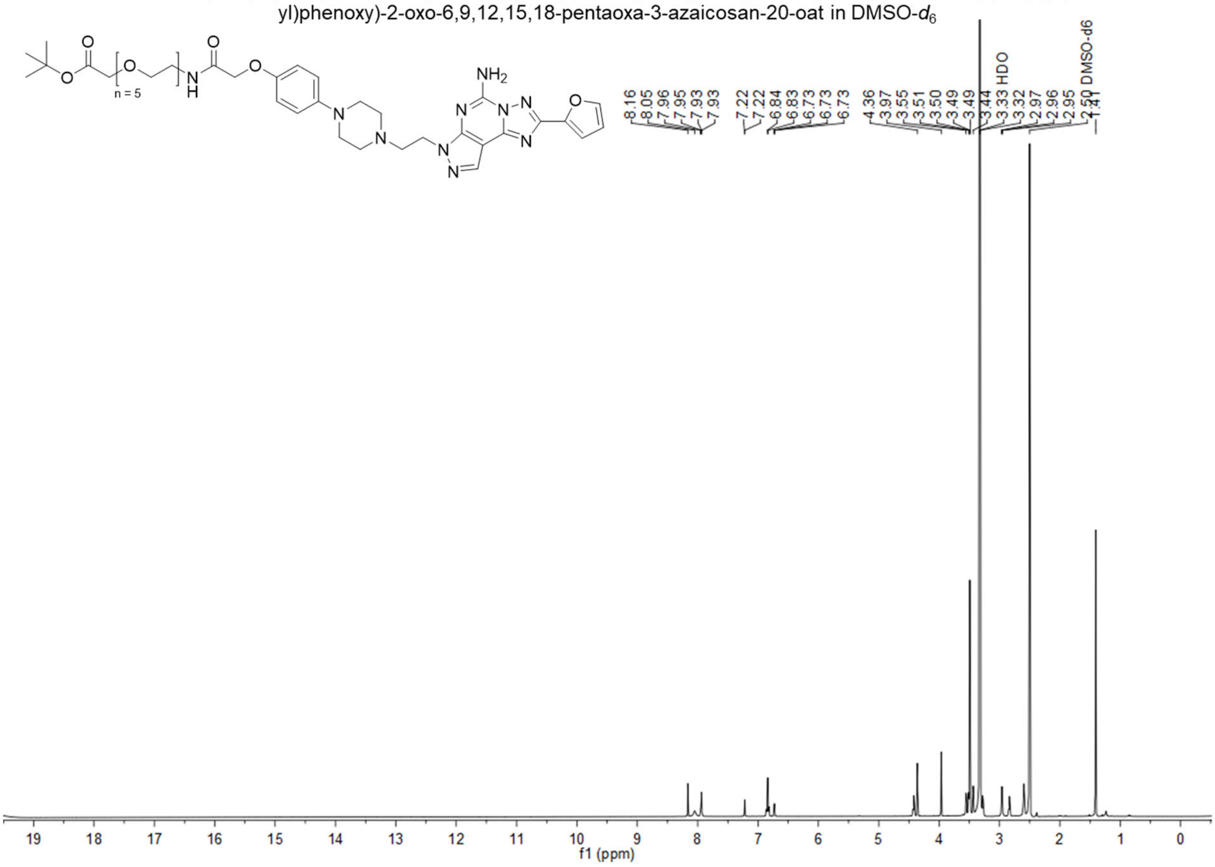

^{13}C-NMR *tert*-Butyl-1-(4-(4-(2-(5-amino-2-(furan-2-yl)-7*H*-pyrazolo[4,3-*e*][1,2,4]triazolo[1,5-*c*]pyrimidin-7-yl)ethyl)piperazin-1-yl)phenoxy)-2-oxo-6,9,12,15,18-pentaoxa-3-azaicosan-20-oat in DMSO-d_6

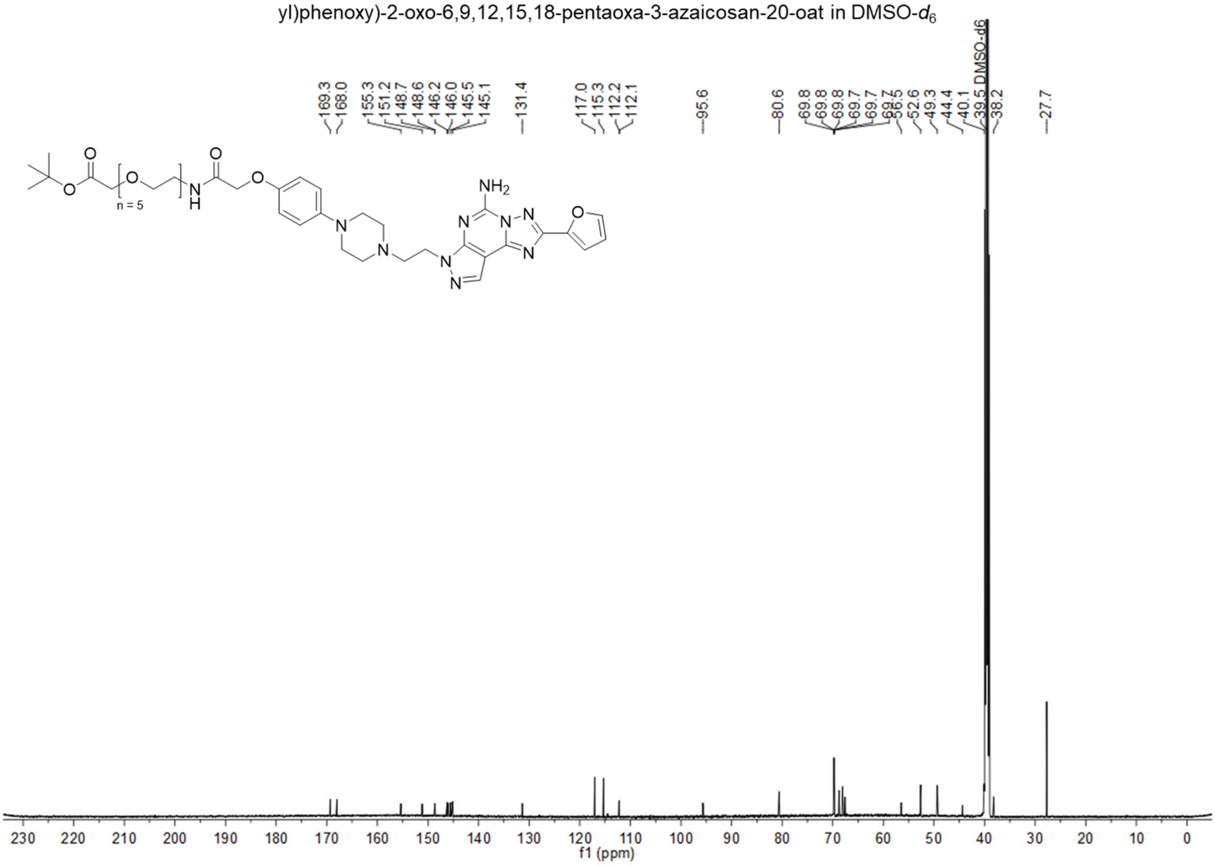

^{1}H-NMR *tert*-Butyl 1-(4-(4-(2-(5-amino-2-(furan-2-yl)-7*H*-pyrazolo[4,3-*e*][1,2,4]triazolo[1,5-*c*]pyrimidin-7-yl)ethyl)piperazin-1-yl)phenoxy)-2-oxo-6,9,12,15,18,21,24,27-octaoxa-3-azanonacosan-29-oat in DMSO-d_6

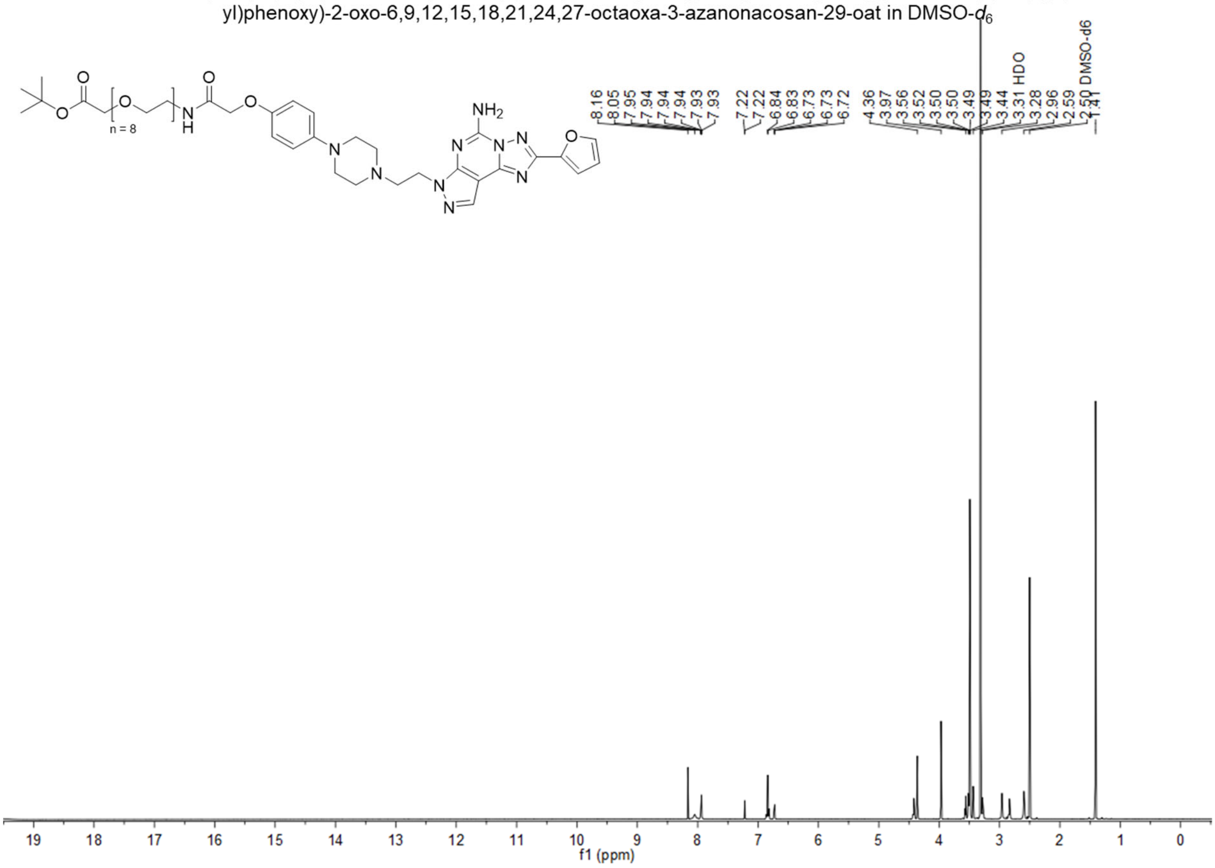

^{13}C-NMR *tert*-Butyl 1-(4-(4-(2-(5-amino-2-(furan-2-yl)-7*H*-pyrazolo[4,3-*e*][1,2,4]triazolo[1,5-*c*]pyrimidin-7-yl)ethyl)piperazin-1-yl)phenoxy)-2-oxo-6,9,12,15,18,21,24,27-octaoxa-3-azanonacosan-29-oat in DMSO-d_6

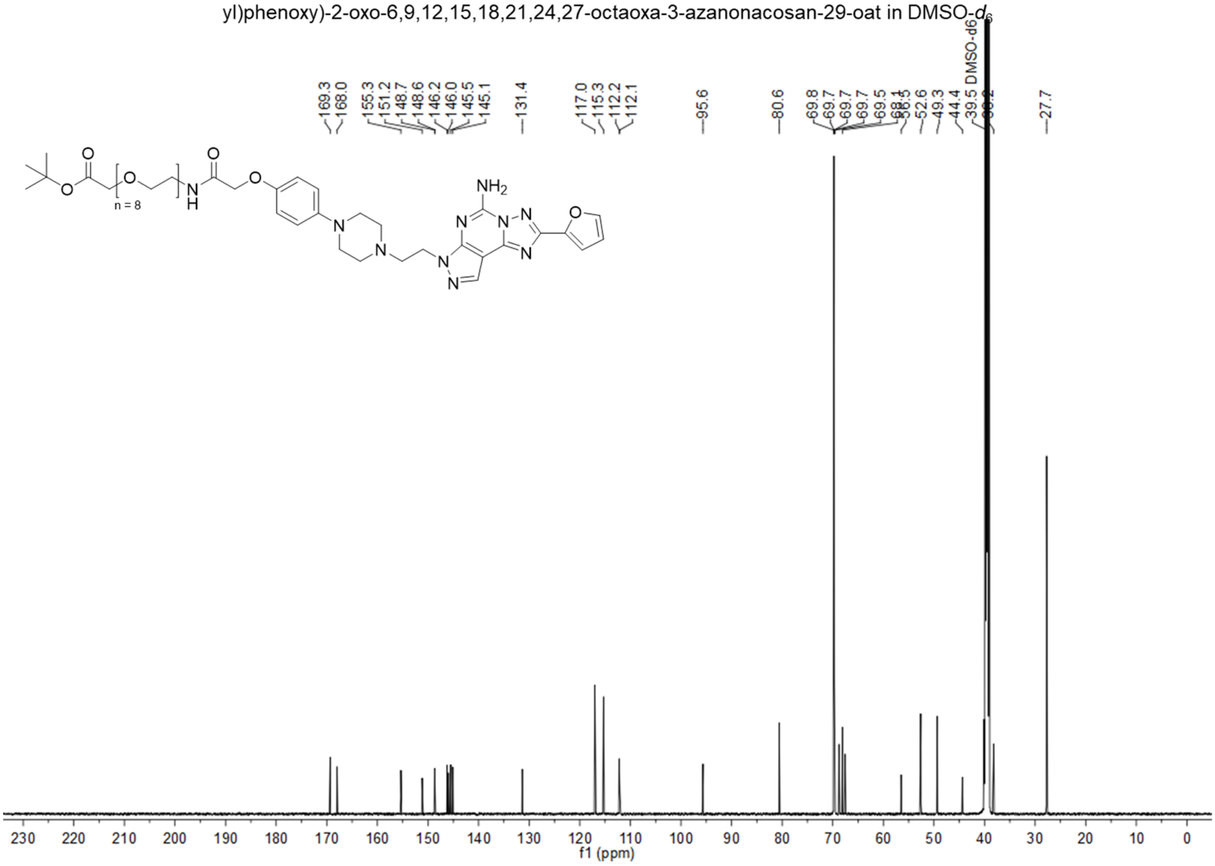

^{1}H-NMR *tert*-Butyl 1-(4-(4-(2-(5-amino-2-(furan-2-yl)-7*H*-pyrazolo[4,3-*e*][1,2,4]triazolo[1,5-*c*]pyrimidin-7-yl)ethyl)piperazin-1-yl)phenoxy)-2-oxo-6,9,12,15,18,21,24,27,30,33,36,39-dodecaoxa-3-azahentetracontan-41-oat in DMSO-d_6

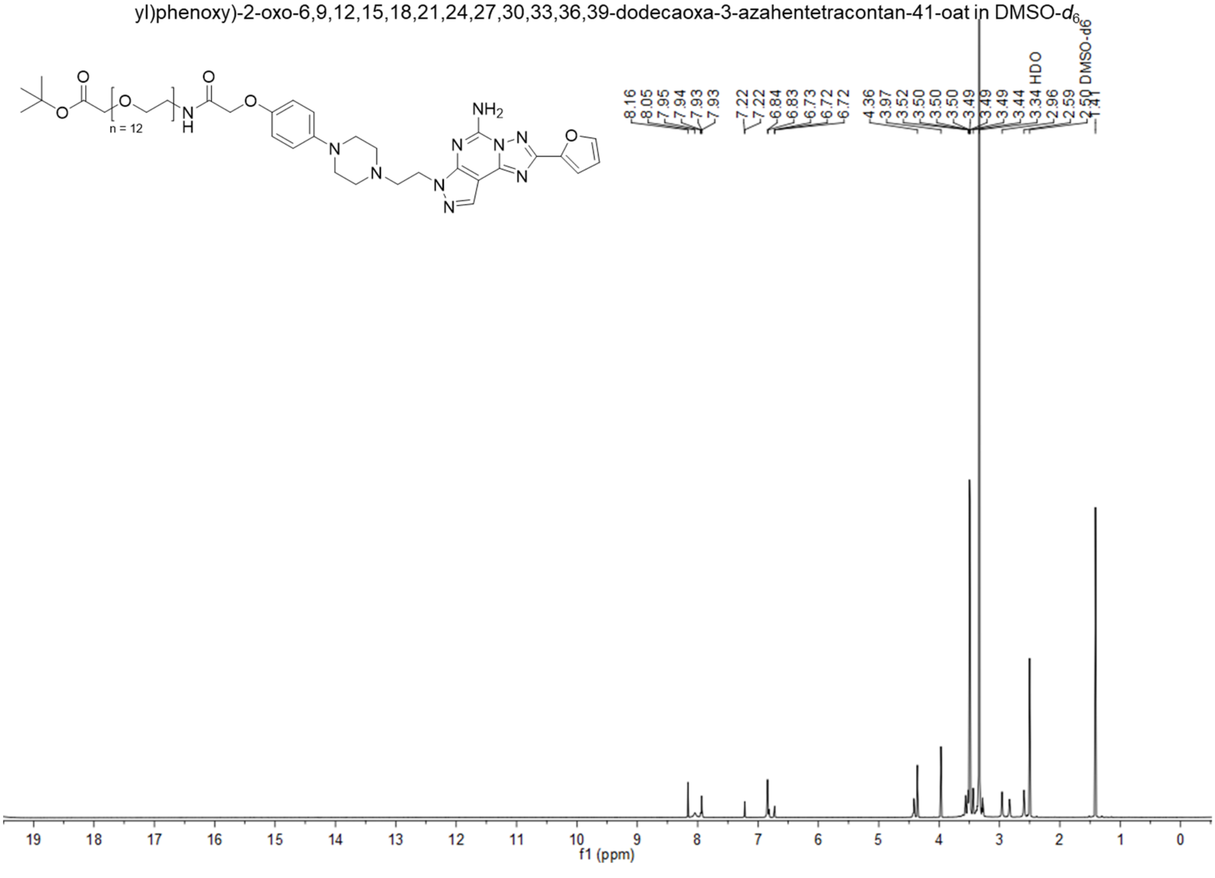

^{13}C-NMR *tert*-Butyl 1-(4-(4-(2-(5-amino-2-(furan-2-yl)-7*H*-pyrazolo[4,3-*e*][1,2,4]triazolo[1,5-*c*]pyrimidin-7-yl)ethyl)piperazin-1-yl)phenoxy)-2-oxo-6,9,12,15,18,21,24,27,30,33,36,39-dodecaoxa-3-azahentetracontan-41-oat in DMSO-d_6

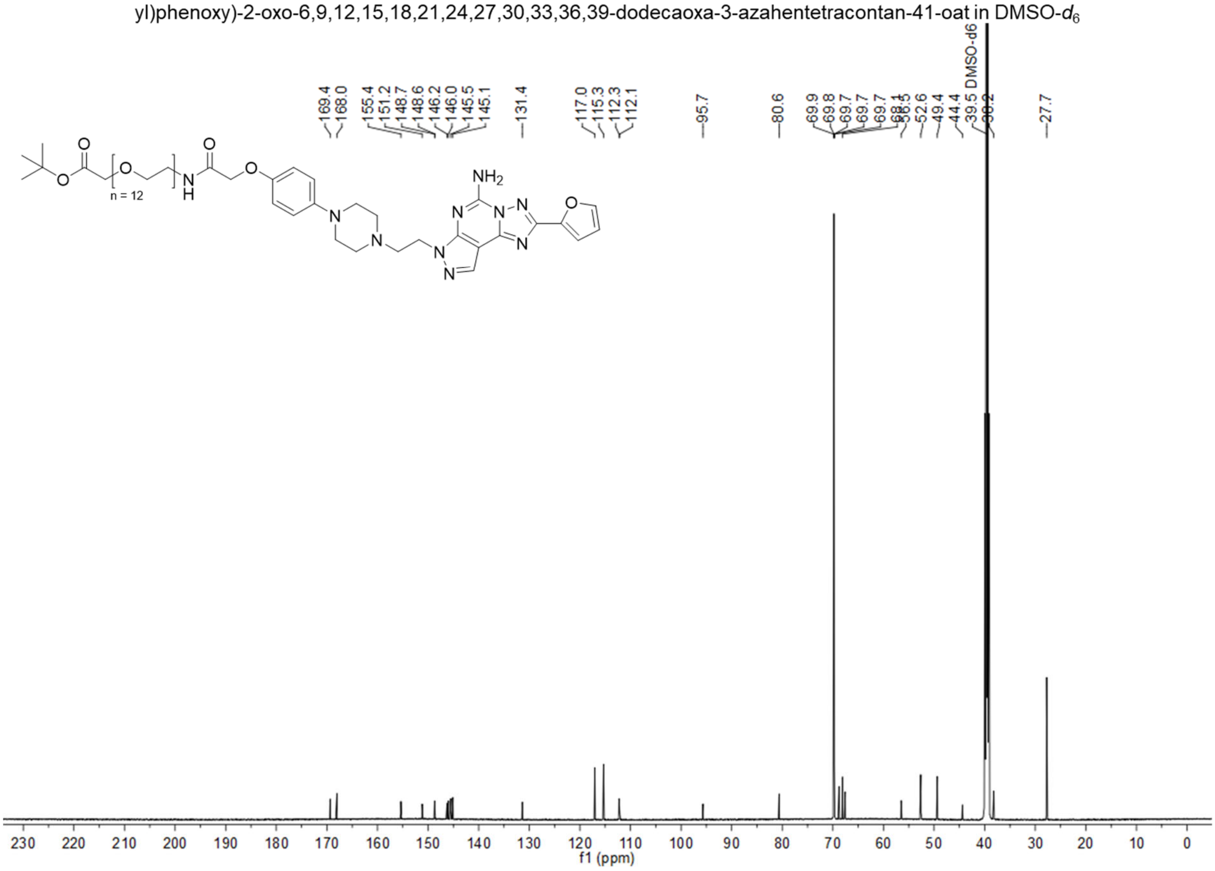

^{1}H-NMR 1-(4-(4-(2-(5-amino-2-(furan-2-yl)-7H-pyrazolo[4,3-e][1,2,4]triazolo[1,5-c]pyrimidin-7-yl)ethyl)piperazin-1-yl)phenoxy)-2-oxo-6,9,12,15,18,21,24,27,30,33,36,39,42,45,48,51-hexadecaoxa-3-azatripentacontan-53-oate in DMSO-d_6

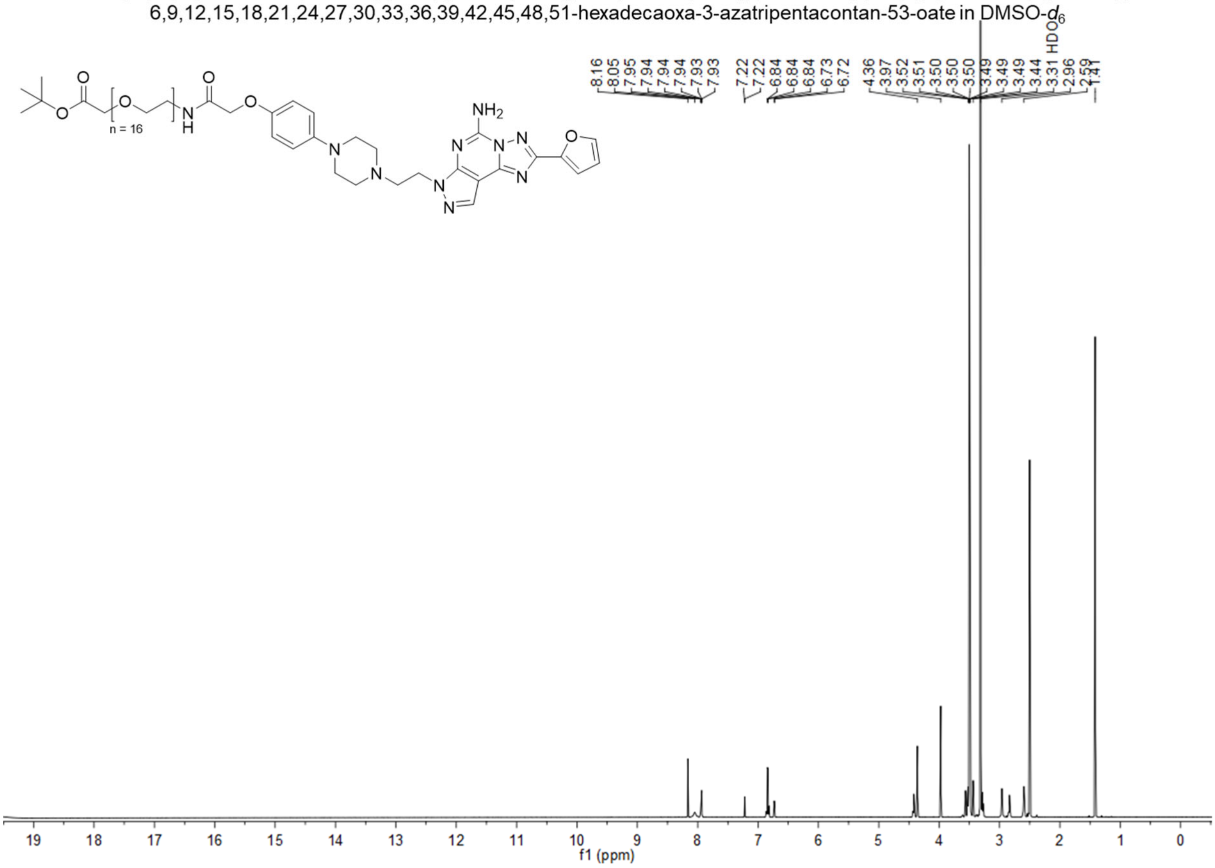

^{13}C-NMR 1-(4-(4-(2-(5-amino-2-(furan-2-yl)-7H-pyrazolo[4,3-e][1,2,4]triazolo[1,5-c]pyrimidin-7-yl)ethyl)piperazin-1-yl)phenoxy)-2-oxo-6,9,12,15,18,21,24,27,30,33,36,39,42,45,48,51-hexadecaoxa-3-azatripentacontan-53-oate in DMSO-d_6

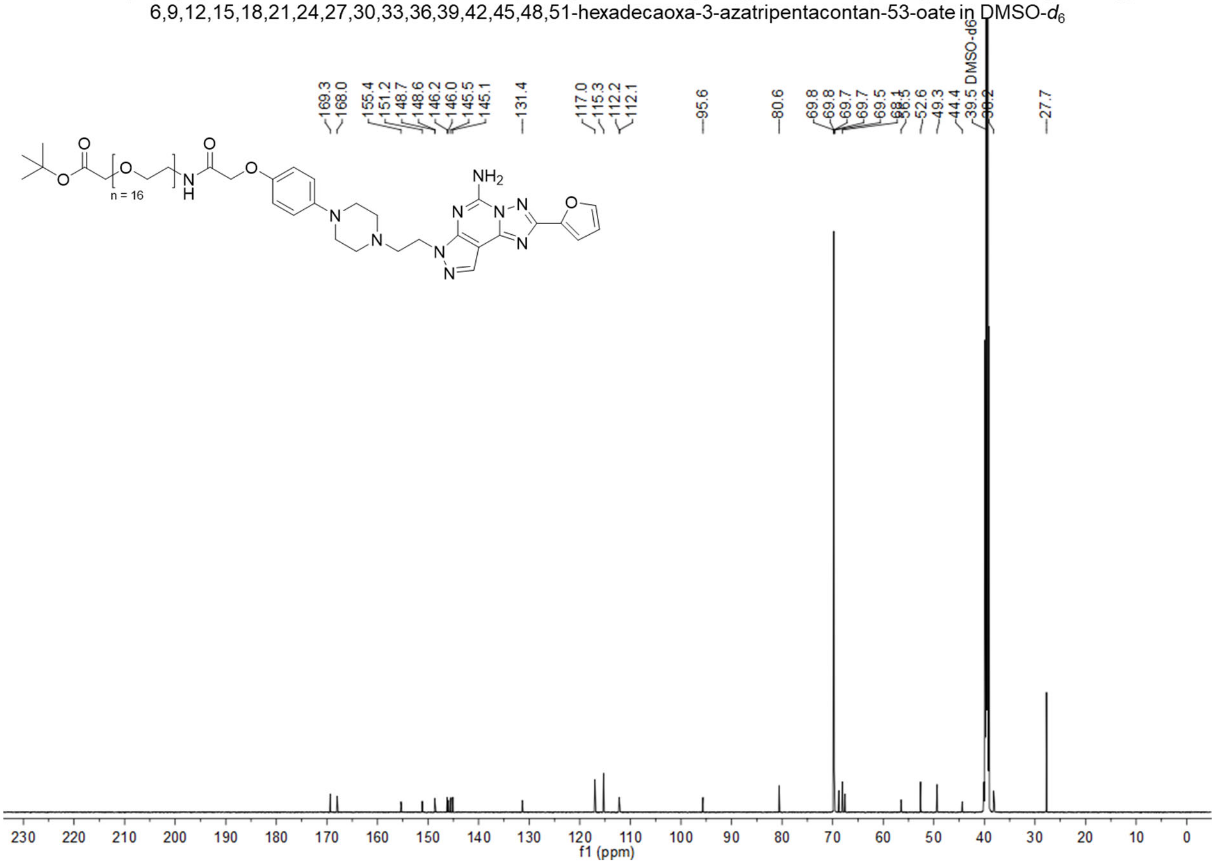

^{1}H-NMR 1-(4-(4-(2-(5-amino-2-(furan-2-yl)-7H-pyrazolo[4,3-e][1,2,4]triazolo[1,5-c]pyrimidin-7-yl)ethyl)piperazin-1-yl)phenoxy)-2-oxo-6,9,12,15,18,21,24,27,30,33,36,39,42,45,48,51,54,57,60,63-icosaoxa-3-azapentahexacontan-65-oate in DMSO-d_6

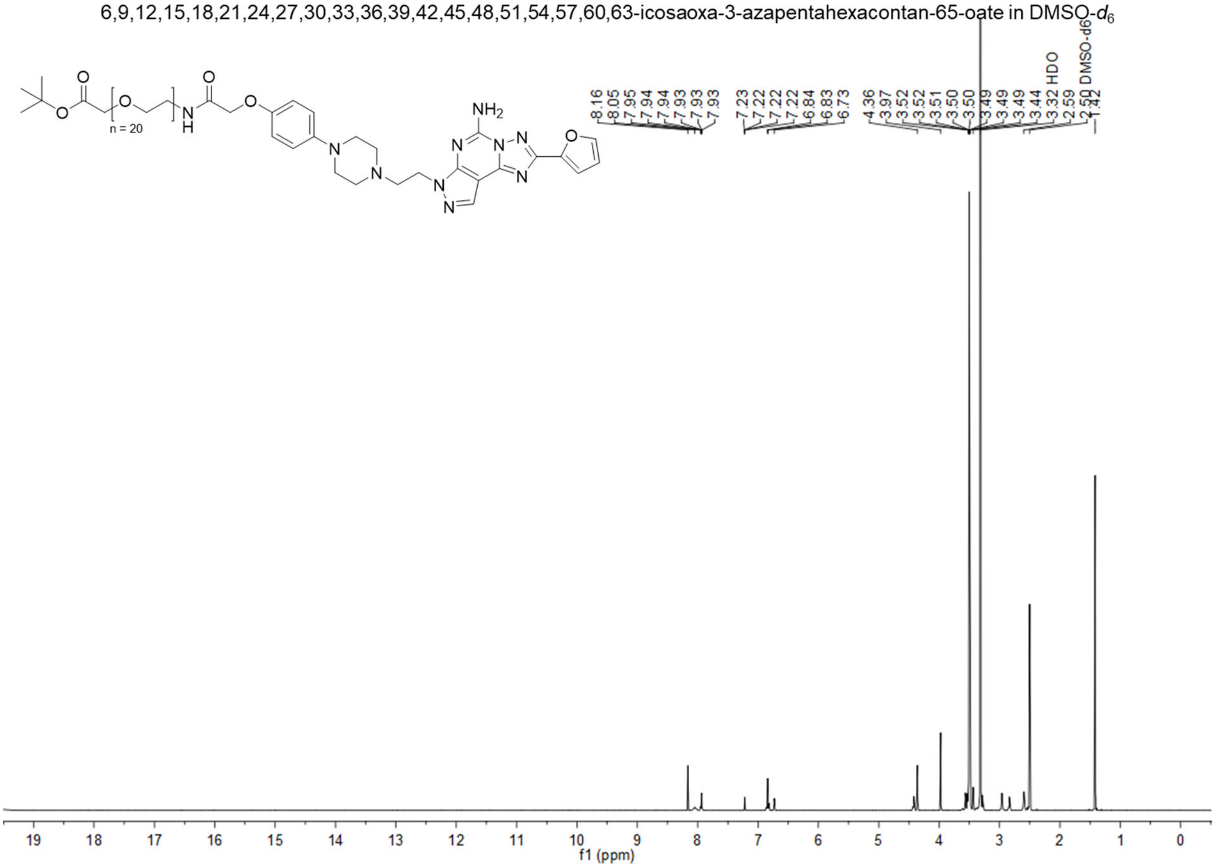

^{13}C-NMR 1-(4-(4-(2-(5-amino-2-(furan-2-yl)-7H-pyrazolo[4,3-e][1,2,4]triazolo[1,5-c]pyrimidin-7-yl)ethyl)piperazin-1-yl)phenoxy)-2-oxo-6,9,12,15,18,21,24,27,30,33,36,39,42,45,48,51,54,57,60,63-icosaoxa-3-azapentahexacontan-65-oate in DMSO-d_6

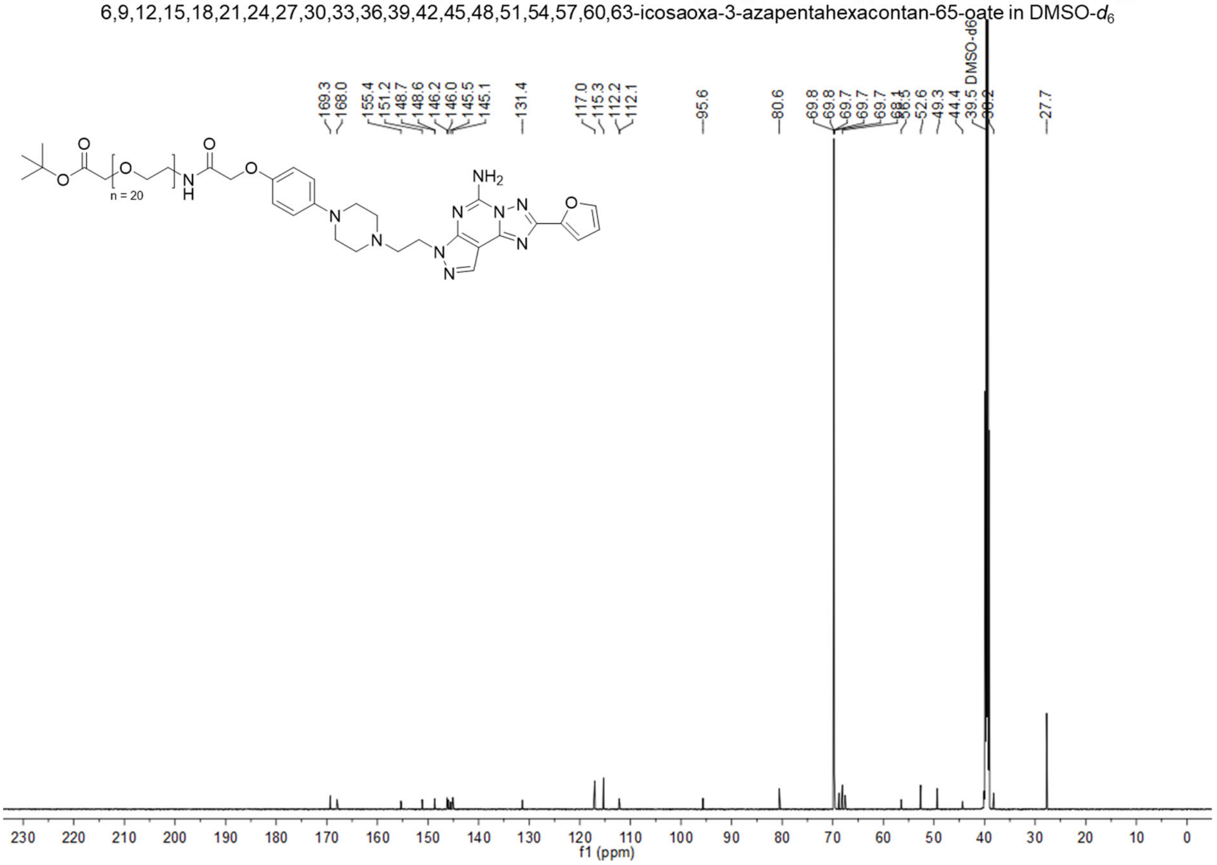

^{1}H-NMR 1-(4-(4-(2-(5-Amino-2-(furan-2-yl)-7*H*-pyrazolo[4,3-*e*][1,2,4]triazolo[1,5-*c*]pyrimidin-7-yl)ethyl)piperazin-1-yl)phenoxy)-2-oxo-6,9,12,15-tetraoxa-3-azaheptadecan-17-säure in DMSO-d_6

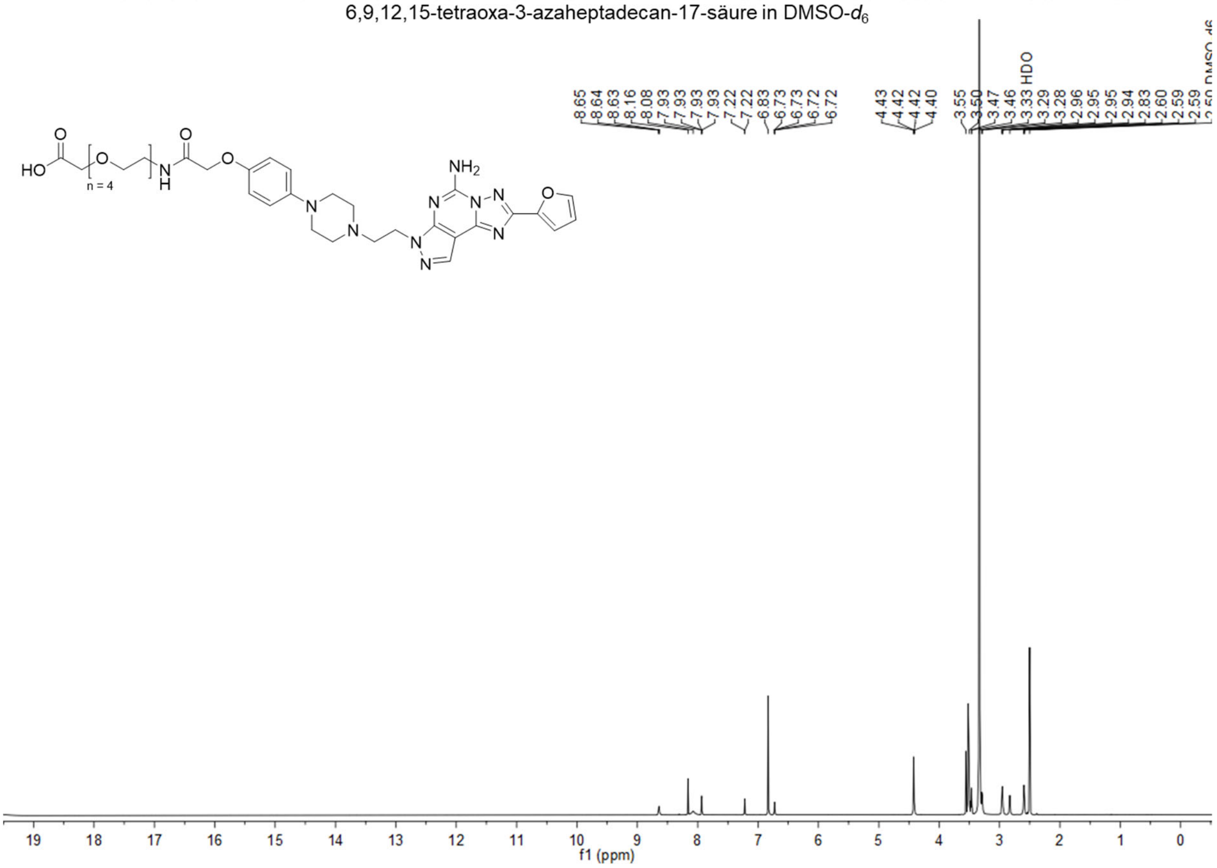

^{13}C-NMR 1-(4-(4-(2-(5-Amino-2-(furan-2-yl)-7*H*-pyrazolo[4,3-*e*][1,2,4]triazolo[1,5-*c*]pyrimidin-7-yl)ethyl)piperazin-1-yl)phenoxy)-2-oxo-6,9,12,15-tetraoxa-3-azaheptadecan-17-säure in DMSO-d_6

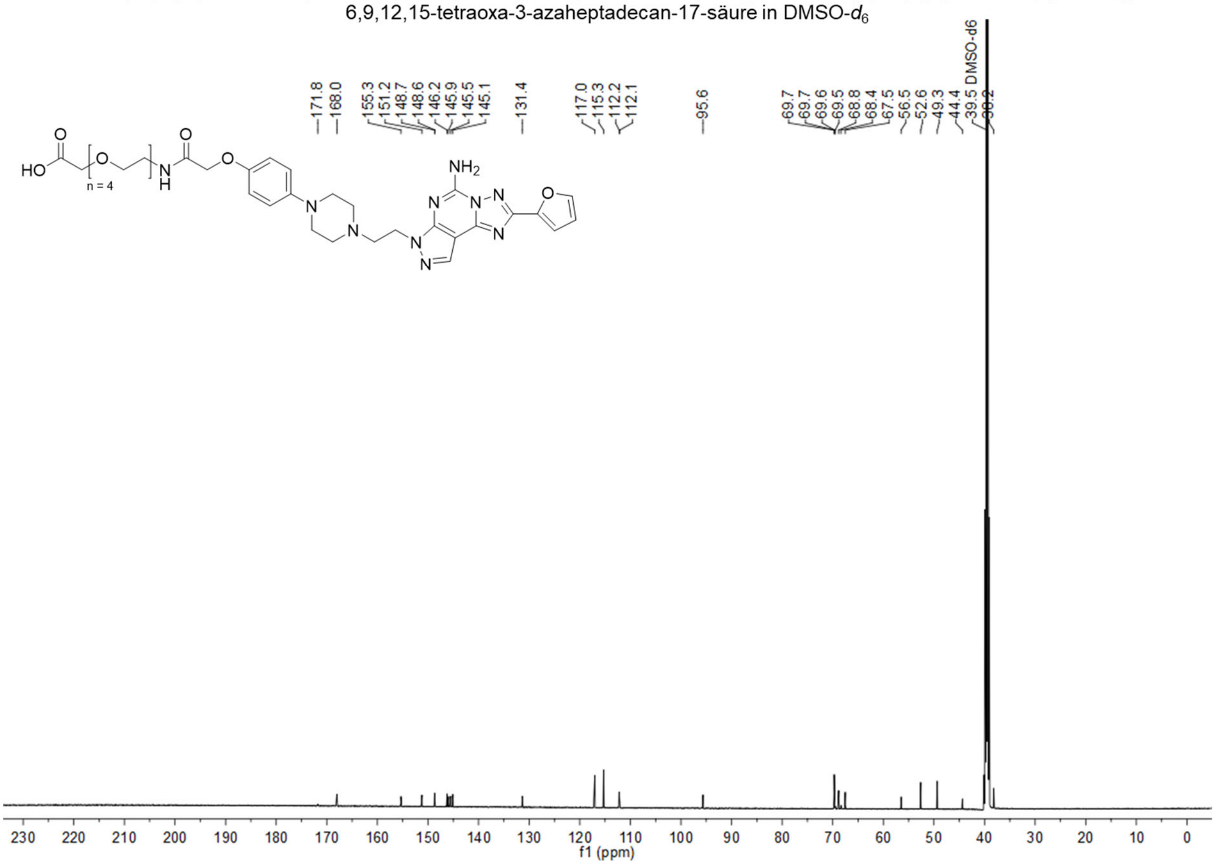

^{1}H-NMR 14-(2-(4-(4-(2-(5-Amino-2-(furan-2-yl)-7*H*-pyrazolo[4,3-*e*][1,2,4]triazolo[1,5-*c*]pyrimidin-7-yl)ethyl)piperazin-1-yl)phenoxy)acetamido)-*N*-(5-(5,5-difluoro-1,3,7,9-tetramethyl-5*H*-4*l*4,5*l*4-dipyrrolo[1,2-*c*:2',1'-*f*][1,3,2]diazaborinin-10-yl)pentyl)-3,6,9,12-tetraoxatetradecanamid in DMSO-d_6

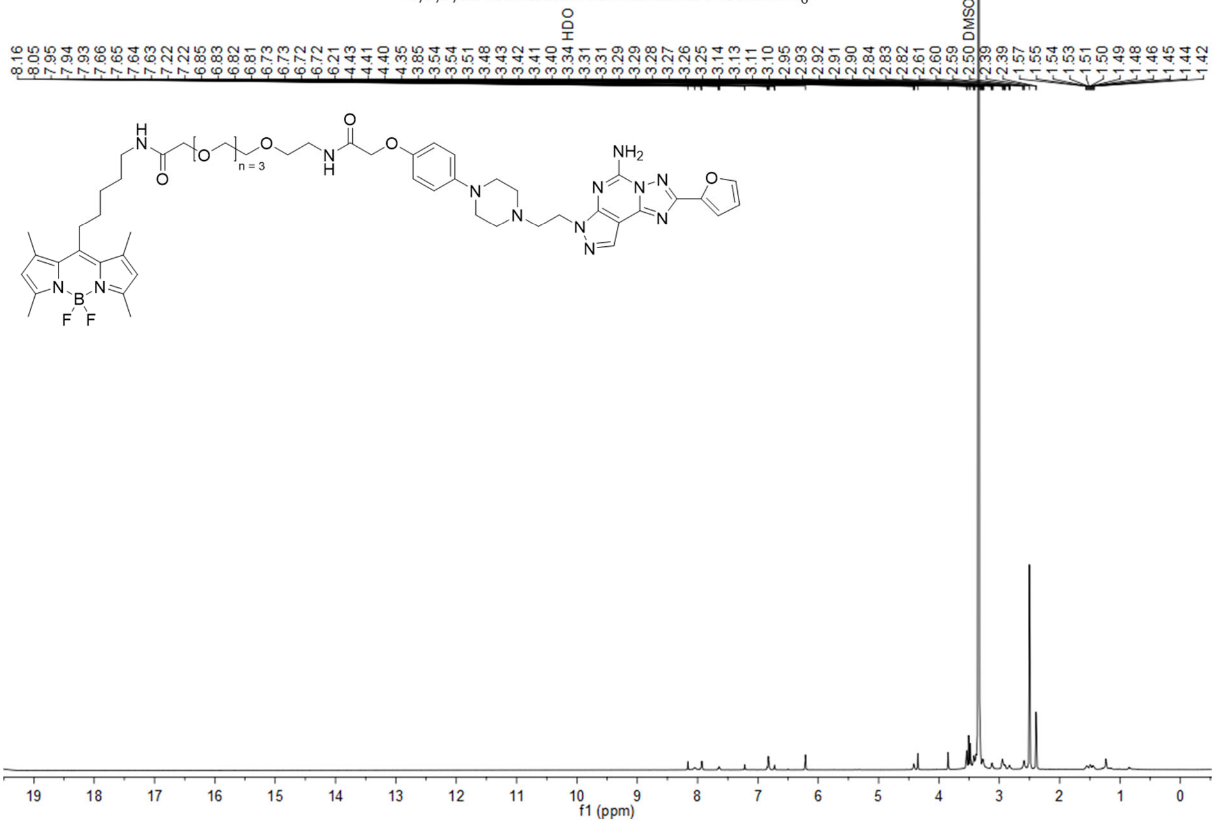

^{13}C-NMR 14-(2-(4-(4-(2-(5-Amino-2-(furan-2-yl)-7*H*-pyrazolo[4,3-e][1,2,4]triazolo[1,5-*c*]pyrimidin-7-yl)ethyl)piperazin-1-yl)phenoxy)acetamido)-*N*-(5-(5,5-difluoro-1,3,7,9-tetramethyl-5*H*-4*l*4,5*l*4-dipyrrolo[1,2-*c*:2',1'-*f*][1,3,2]diazaborinin-10-yl)pentyl)-3,6,9,12-tetraoxatetradecanamid in DMSO-d_6

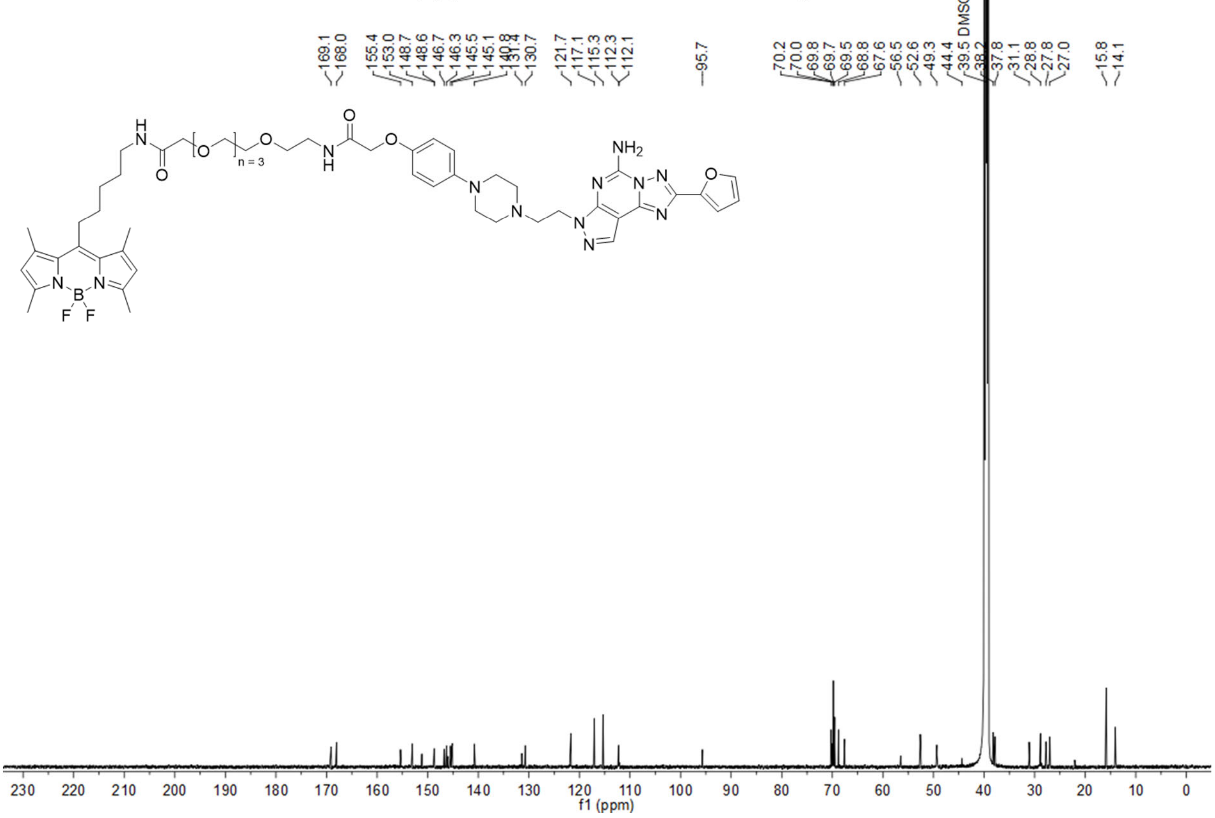

^{1}H-NMR *tert*-Butyl-4-(piperazin-1-yl)benzoat in $CDCl_3$

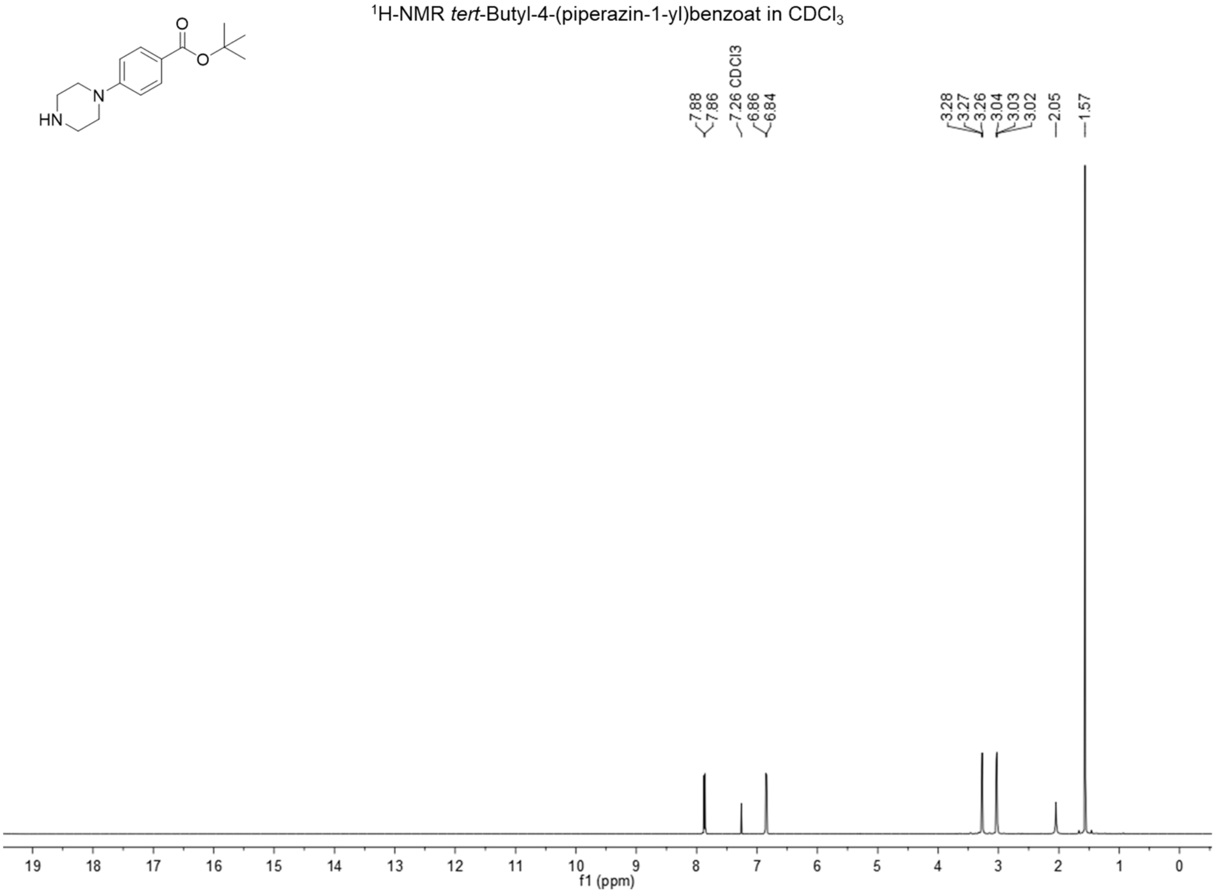

^{13}C-NMR *tert*-Butyl-4-(piperazin-1-yl)benzoat in $CDCl_3$

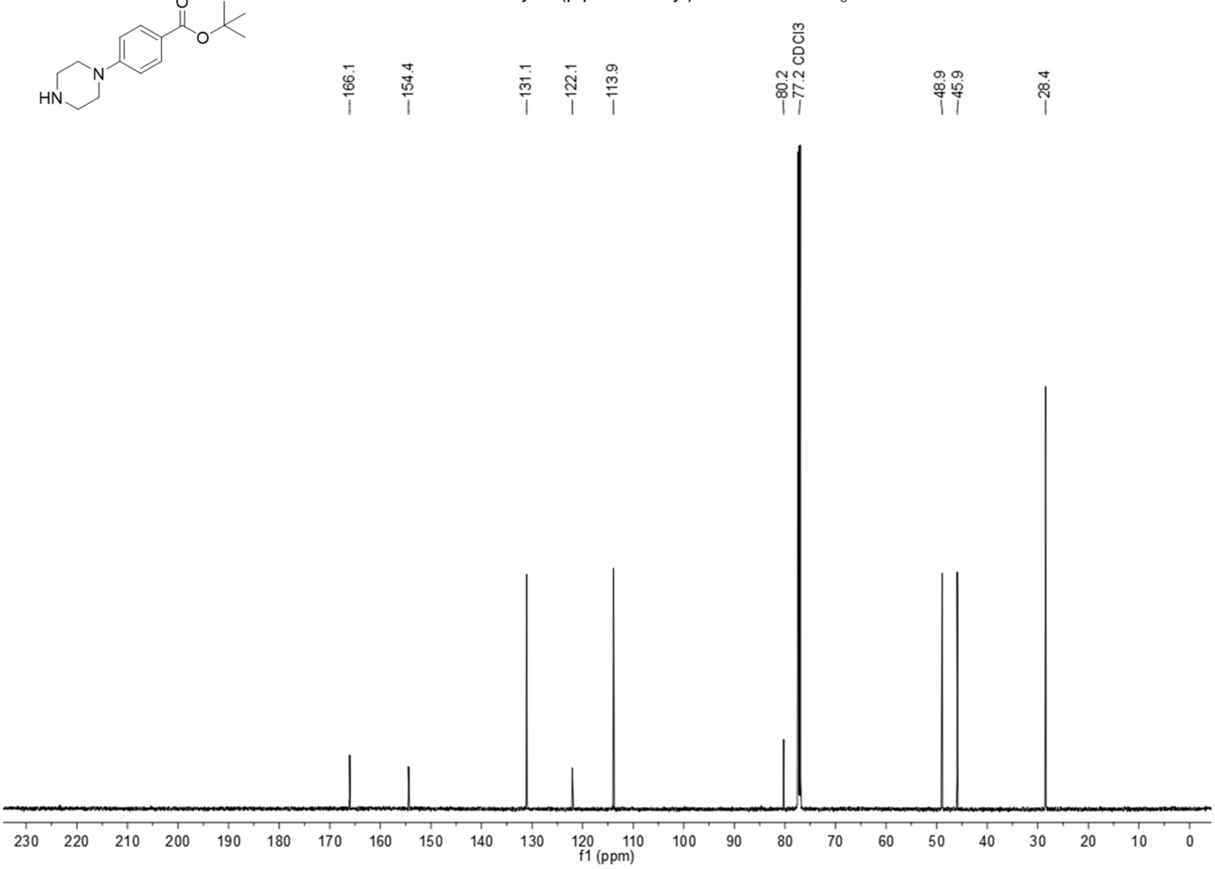

^{1}H-NMR Benzyl-4-(4-(2-(*tert*-butoxy)-2-oxoethoxy)phenyl)piperazin-1-carboxylat in DMSO-d_6

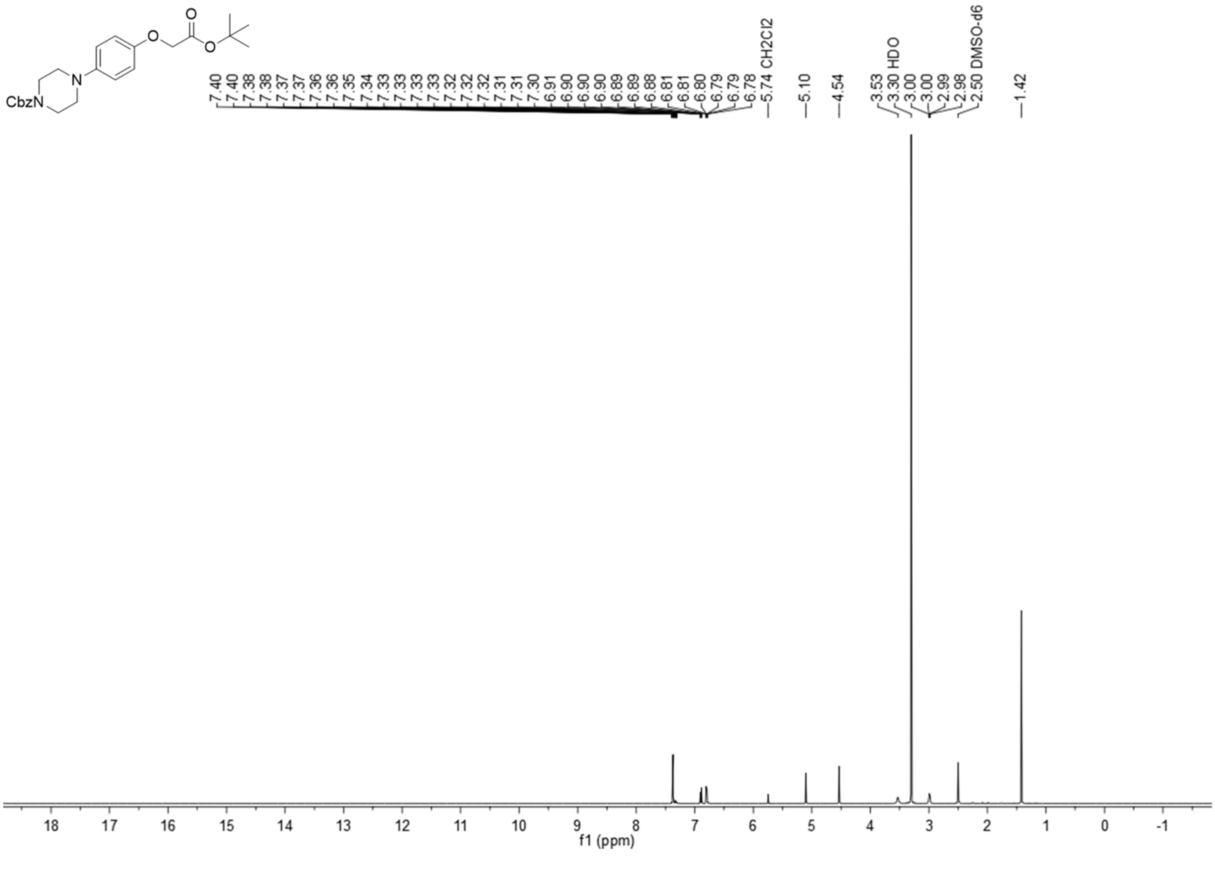

^{13}C-NMR Benzyl-4-(4-(2-(*tert*-butoxy)-2-oxoethoxy)phenyl)piperazin-1-carboxylat in DMSO-d_6

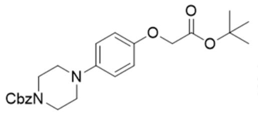

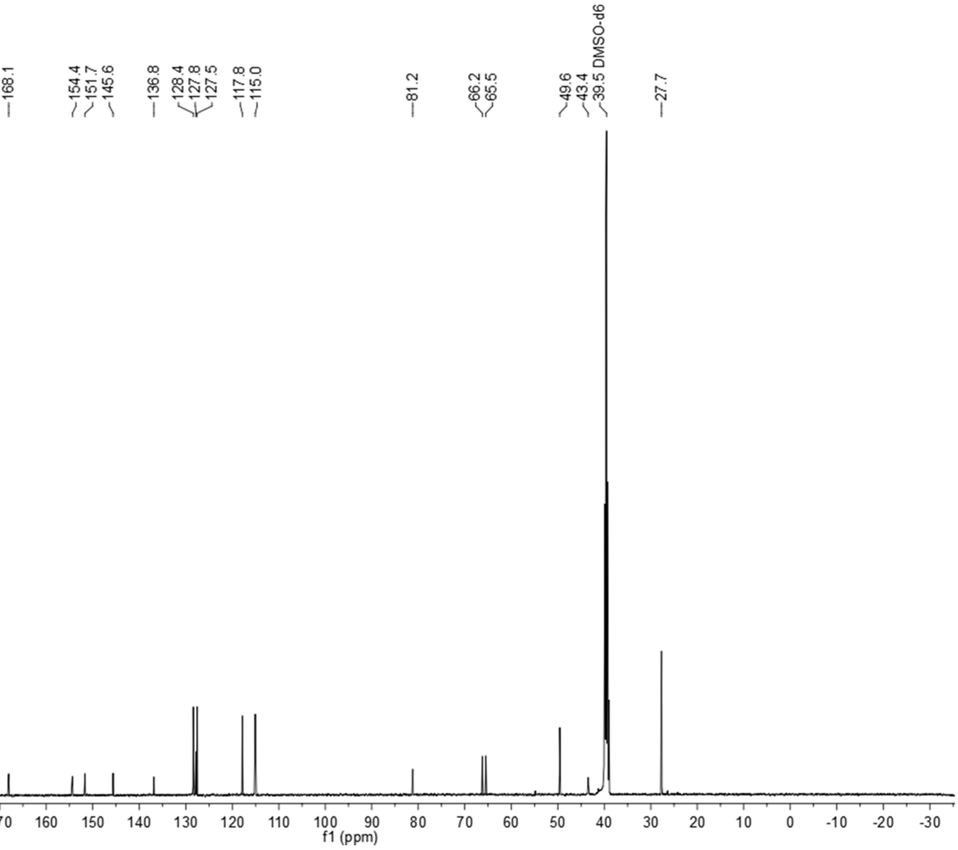

^{1}H-NMR *tert*-Butyl-2-(4-(piperazin-1-yl)phenoxy)acetat in DMSO-d_6

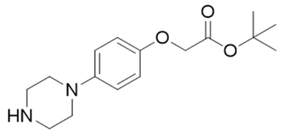

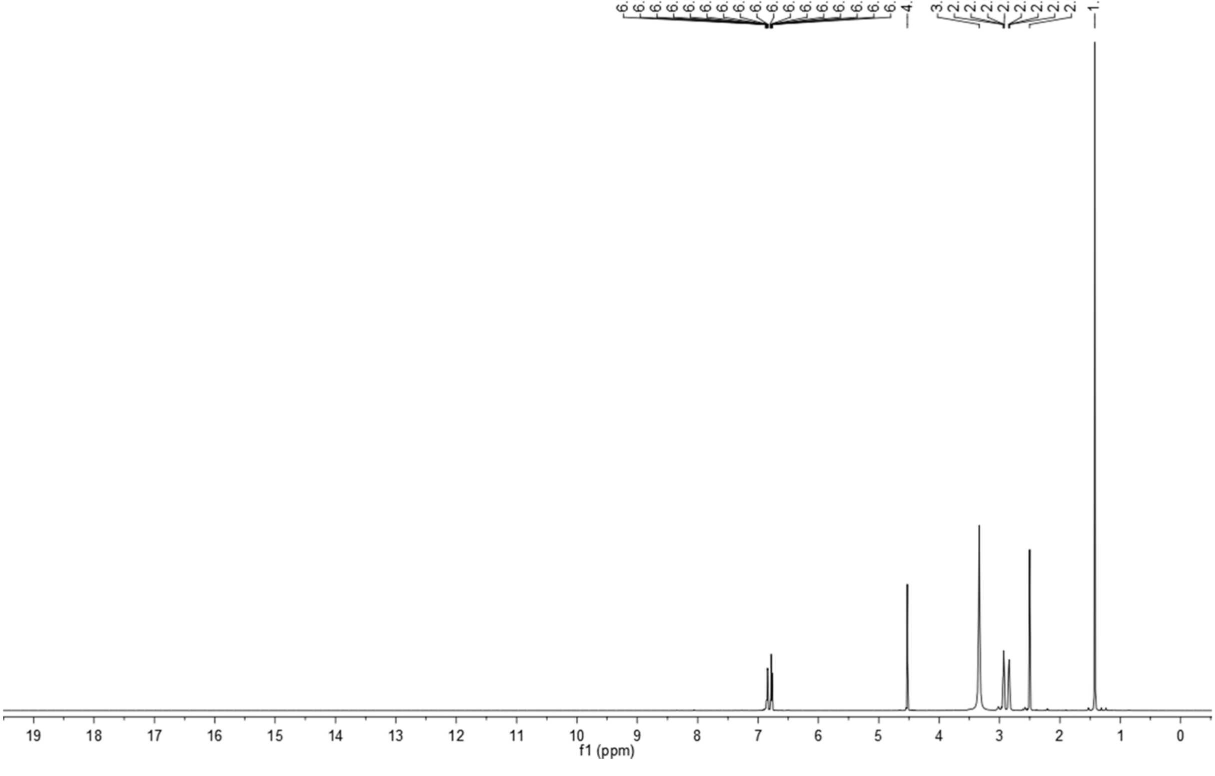

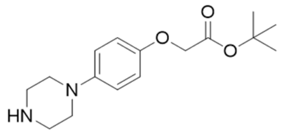

^{13}C-NMR *tert*-Butyl-2-(4-(piperazin-1-yl)phenoxy)acetat in DMSO-d_6

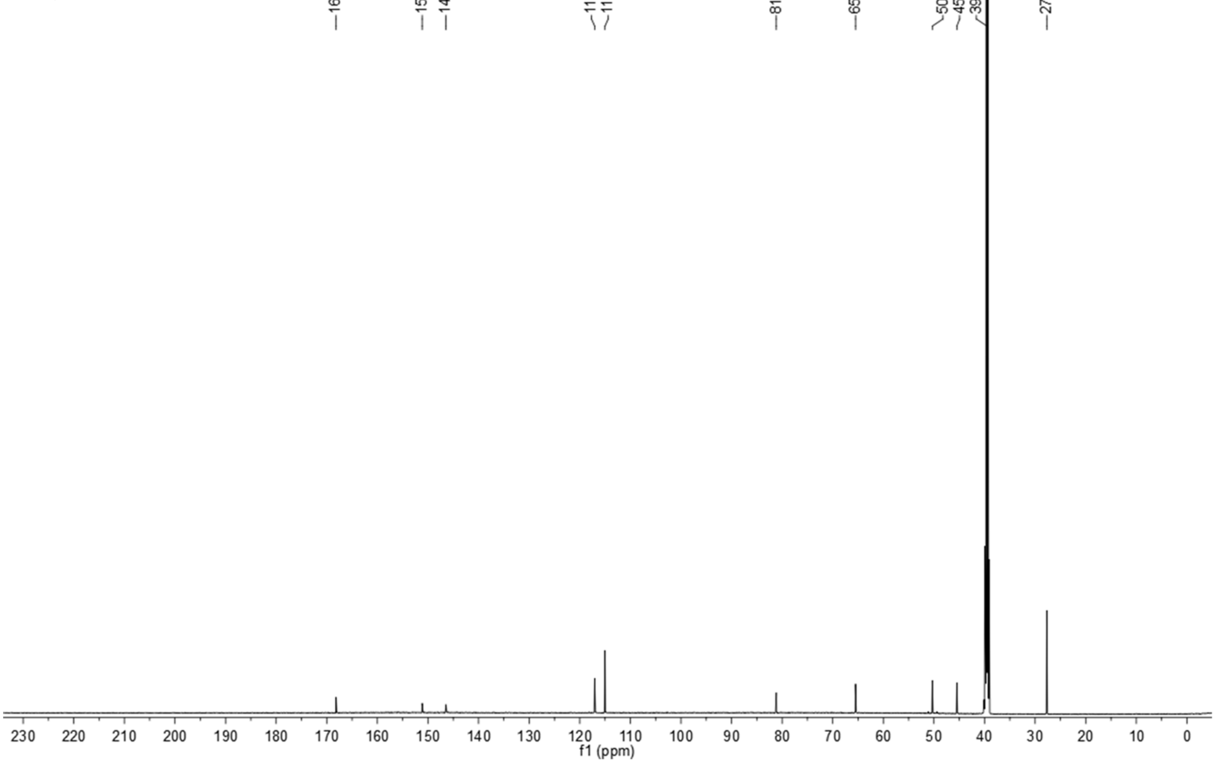

^{1}H-NMR (*E*/*Z*)-*N*'-(6-Chloro-5-formyl-2-imino-2,3-dihydropyrimidin-4(1*H*)-yliden)furan-2-carbohydrazid in DMSO-d_6

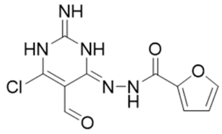

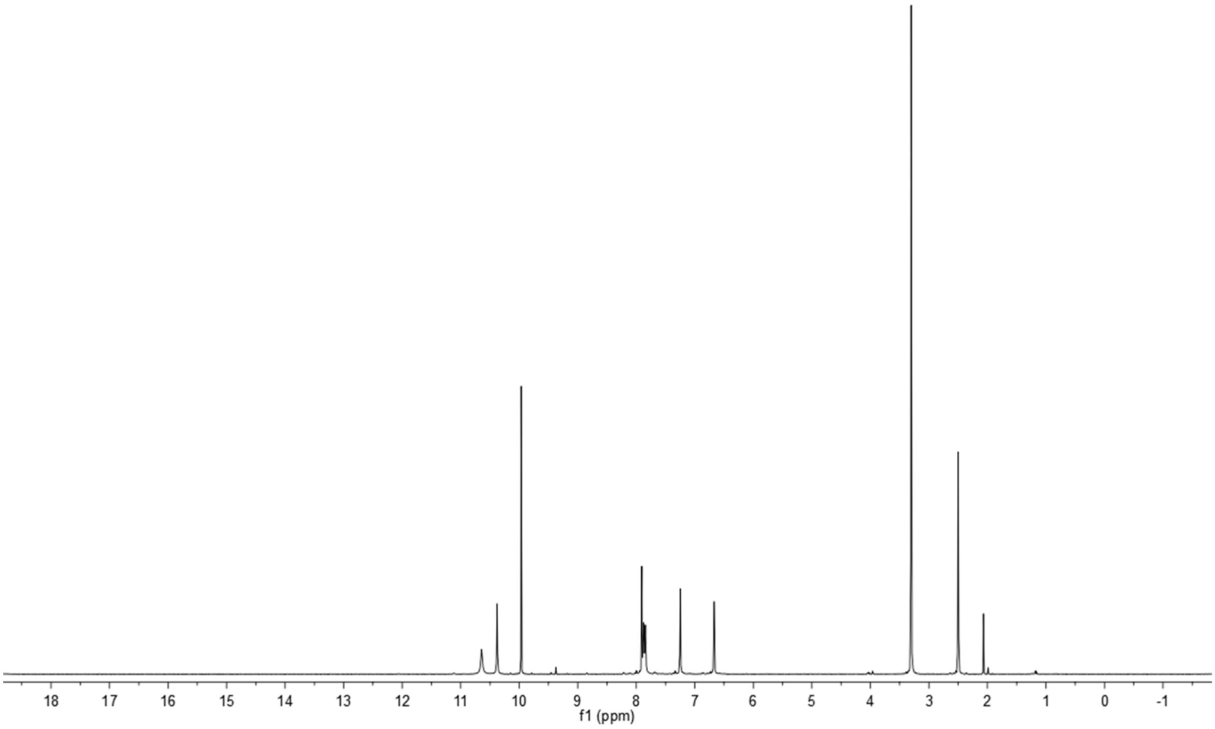

^{13}C-NMR (*E*/*Z*)-*N*'-(6-Chloro-5-formyl-2-imino-2,3-dihydropyrimidin-4(1*H*)-yliden)furan-2-carbohydrazid in DMSO-d_6

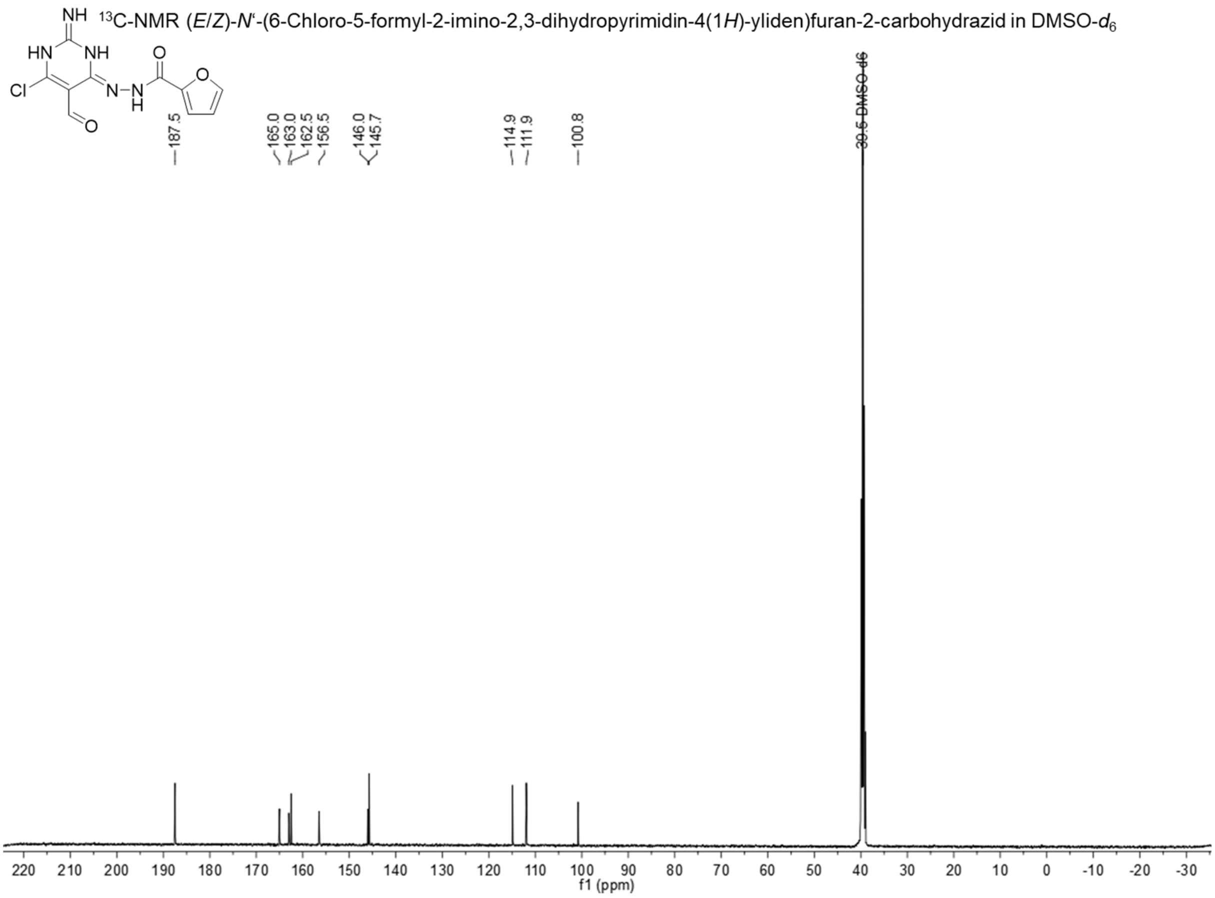

^{1}H-NMR 2-(Furan-2-yl)-7*H*-pyrazolo[4,3-*e*][1,2,4]triazolo[1,5-*c*]pyrimidin-5-amin in DMSO-d_6

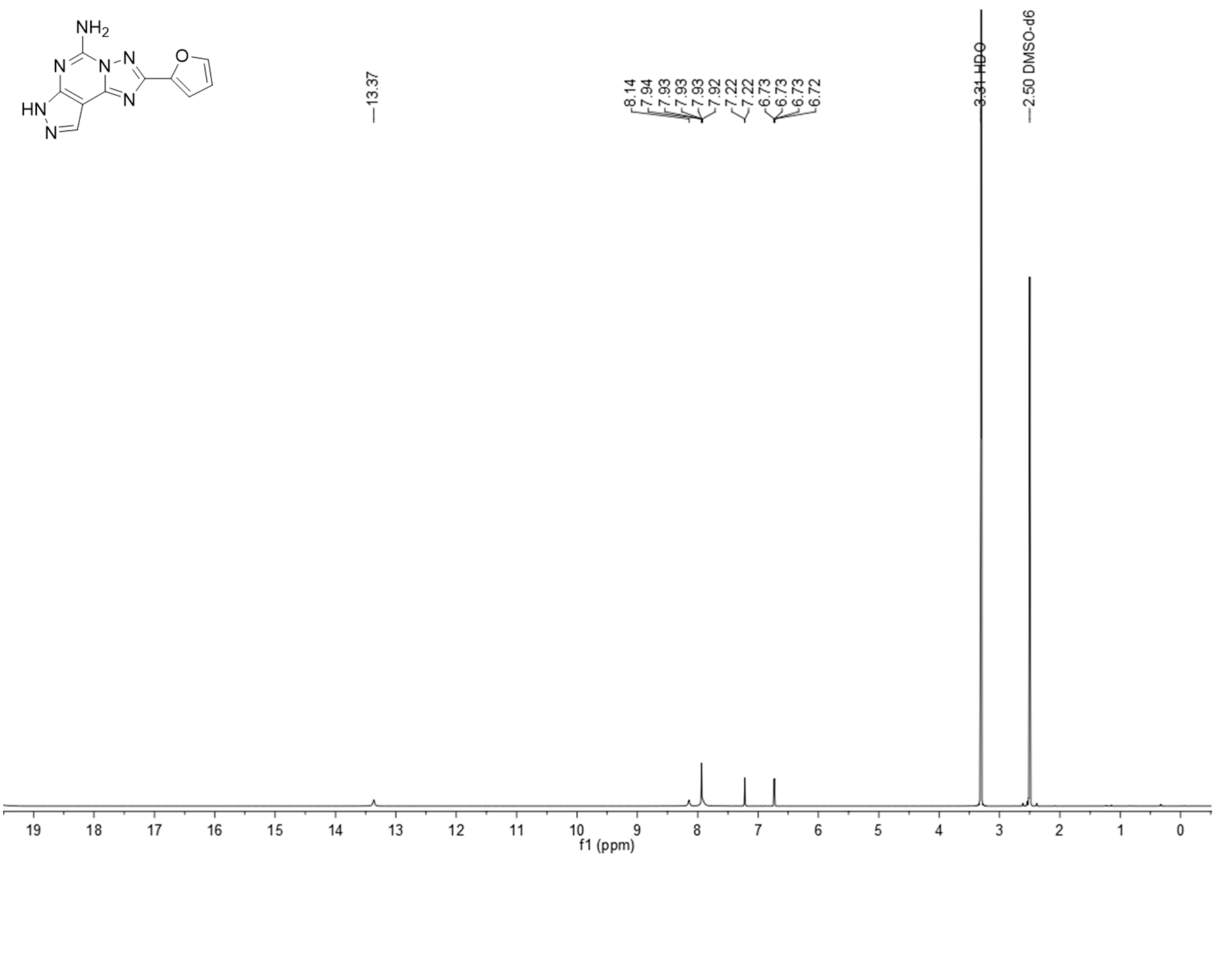

^{13}C-NMR 2-(Furan-2-yl)-7*H*-pyrazolo[4,3-*e*][1,2,4]triazolo[1,5-*c*]pyrimidin-5-amin in DMSO-d_6

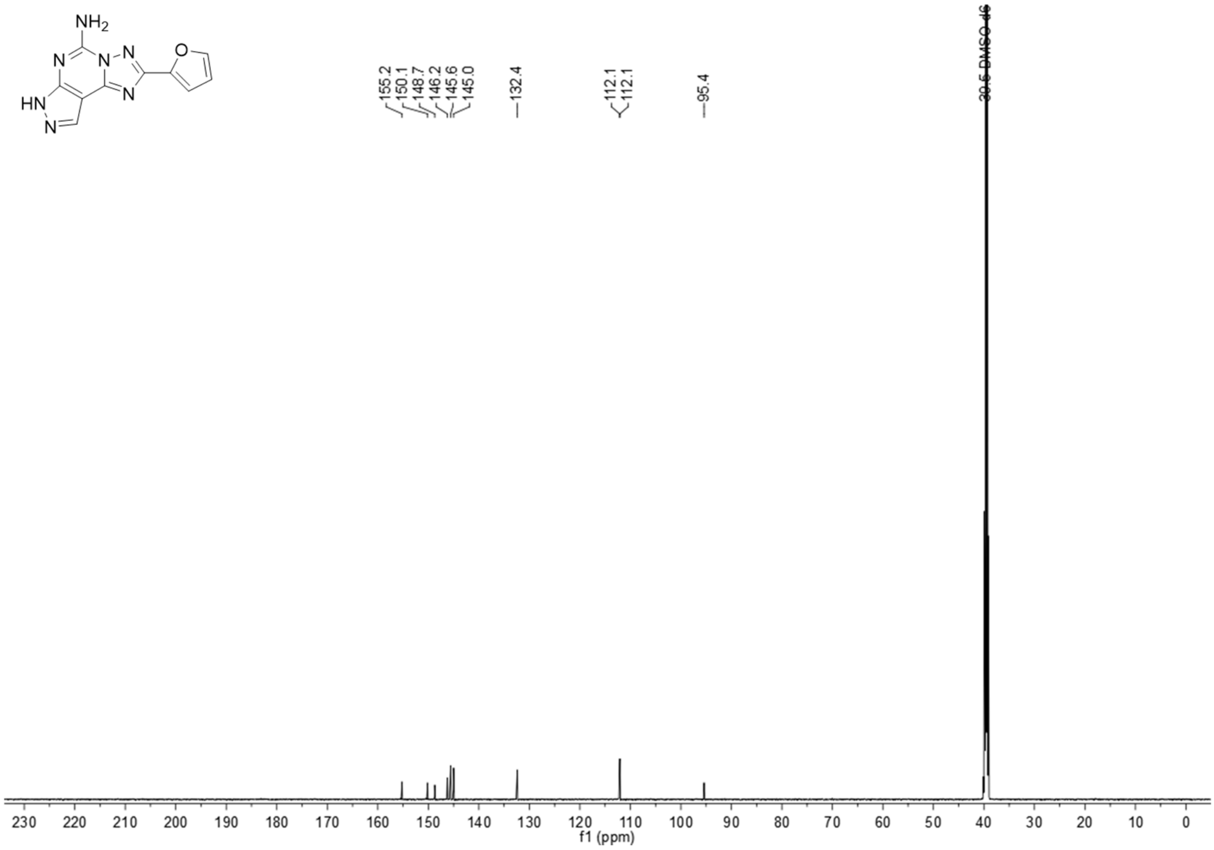

^{1}H-NMR 2-(5-Amino-2-(furan-2-yl)-7*H*-pyrazolo[4,3-*e*][1,2,4]triazolo[1,5-*c*]pyrimidin-7-yl)-ethyl-4-methylbenzenesulfonat in DMSO-d_6

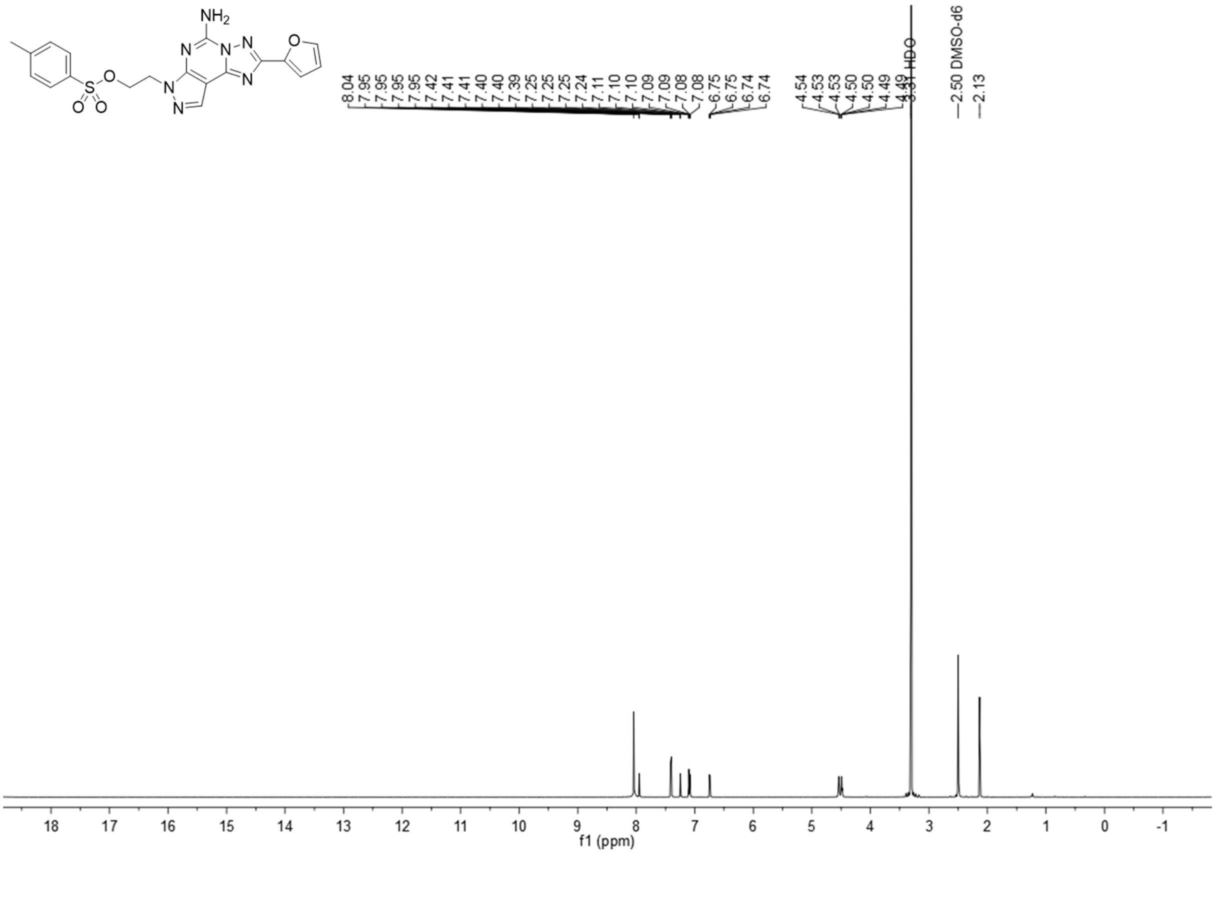

^{13}C-NMR 2-(5-Amino-2-(furan-2-yl)-7*H*-pyrazolo[4,3-*e*][1,2,4]triazolo[1,5-*c*]pyrimidin-7-yl)-ethyl-4-methylbenzenesulfonat in DMSO-d_6

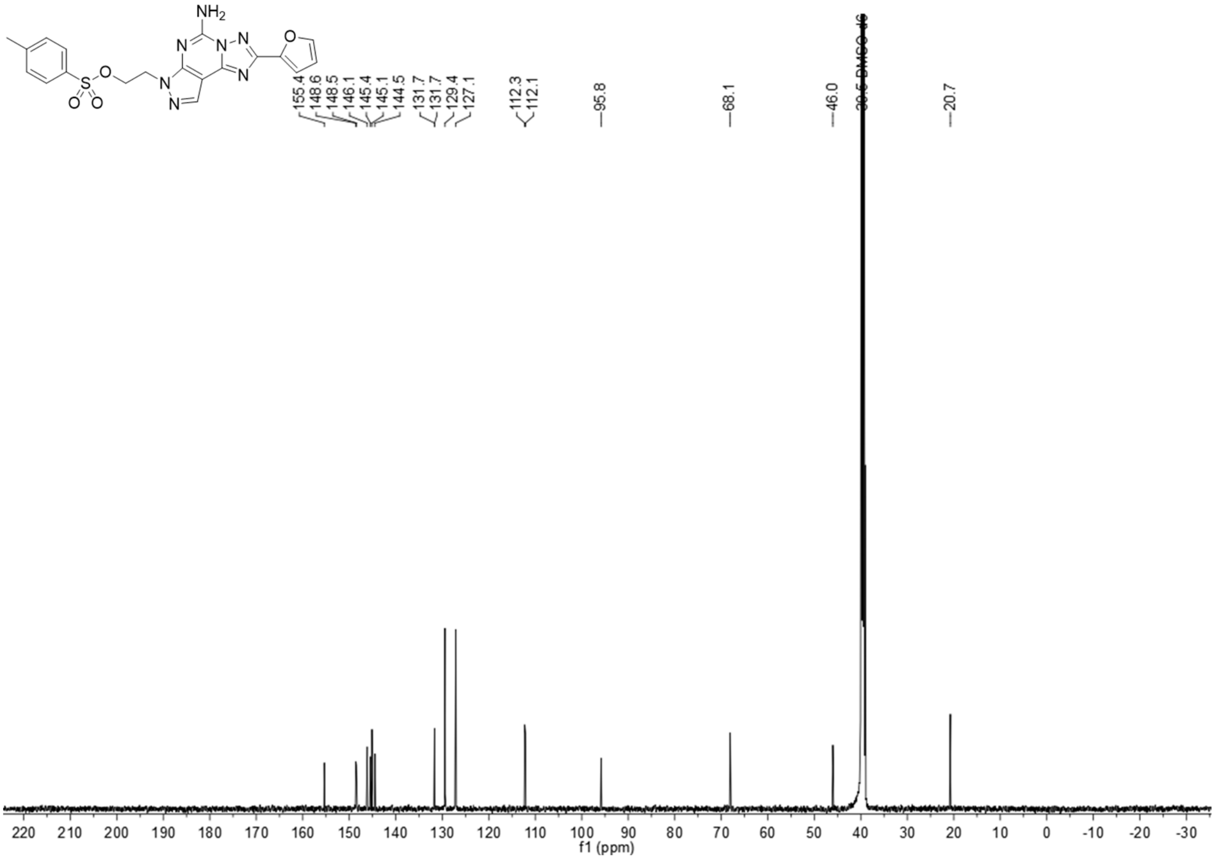

^{1}H-NMR *tert*-Butyl-14-amino-3,6,9,12-tetraoxatetradecanoat in DMSO-d_6

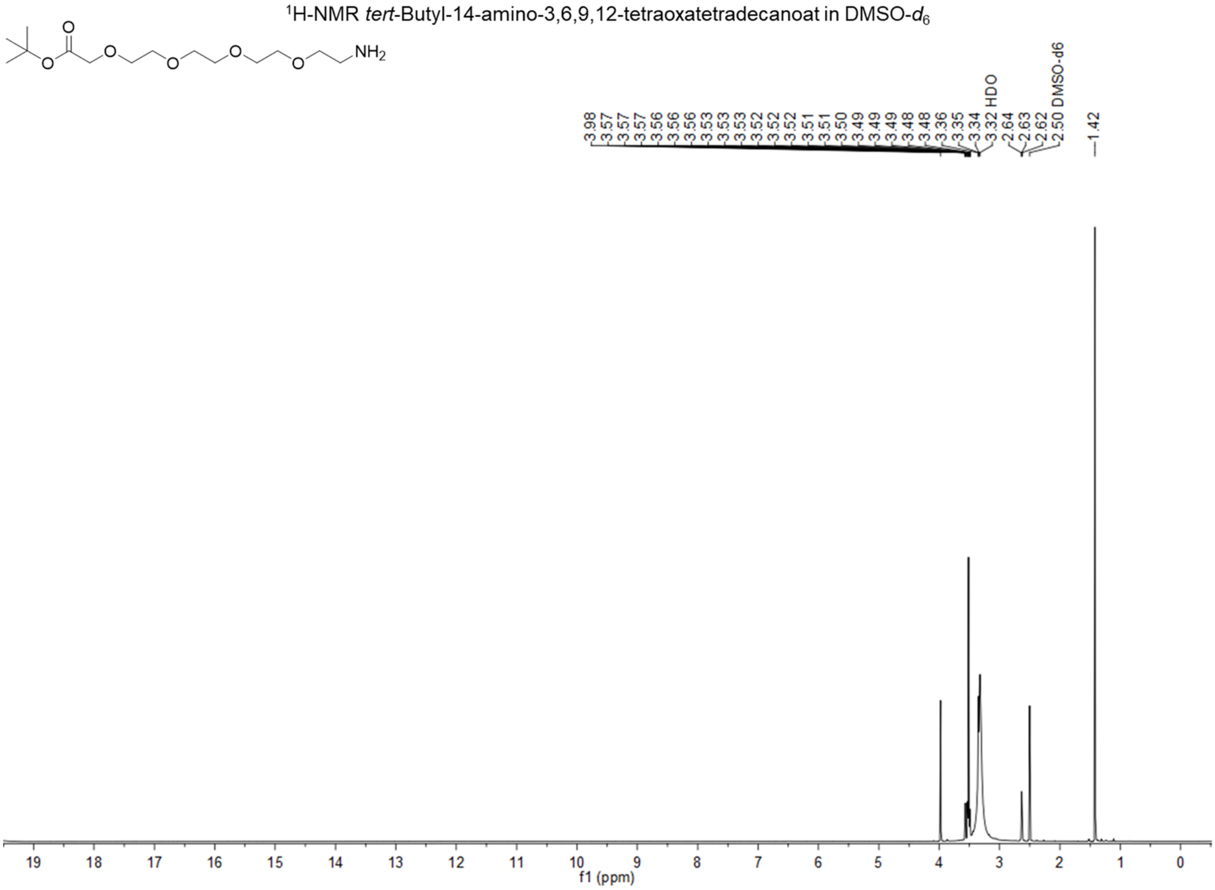

^{13}C-NMR *tert*-Butyl-14-amino-3,6,9,12-tetraoxatetradecanoat in DMSO-d_6

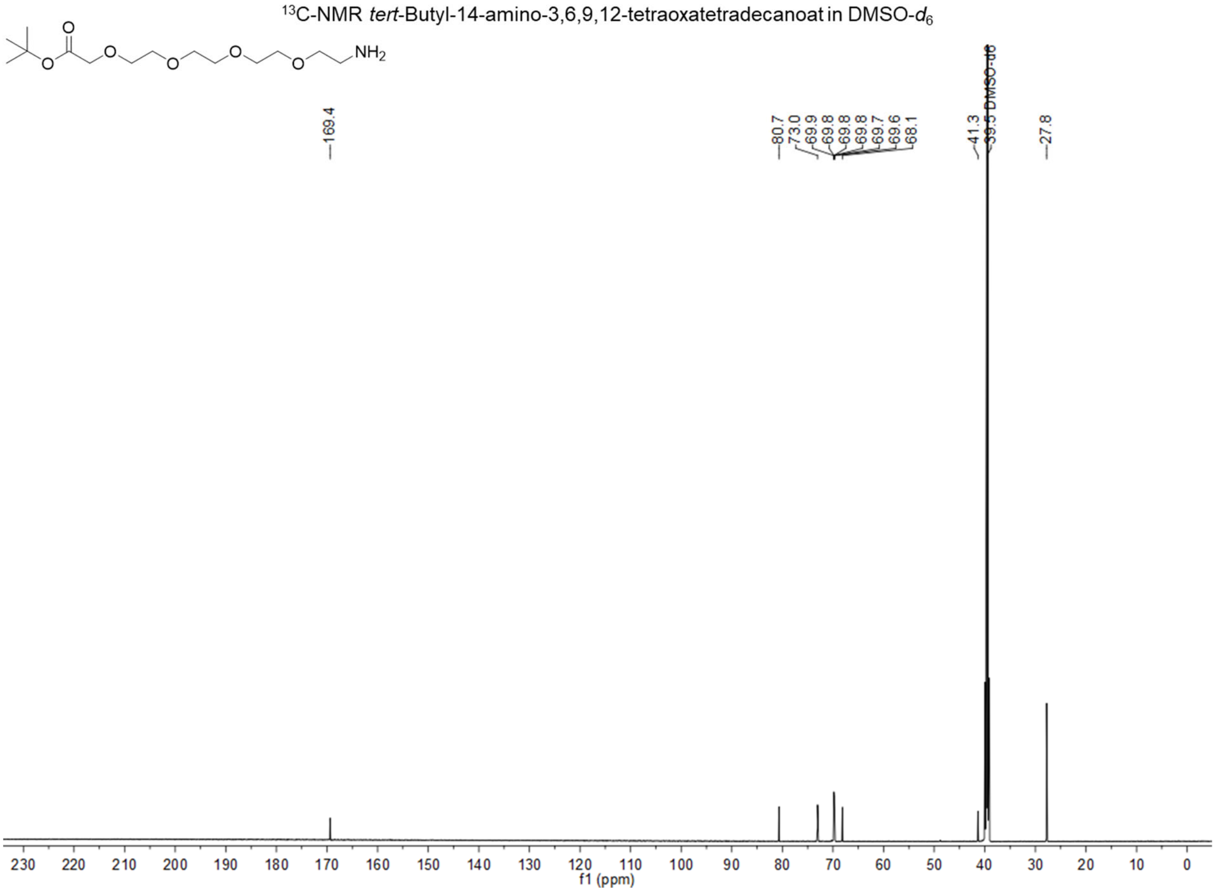

^{1}H-NMR *tert*-Butyl-17-amino-3,6,9,12,15-pentaoxaheptadecanoat in DMSO-d_6

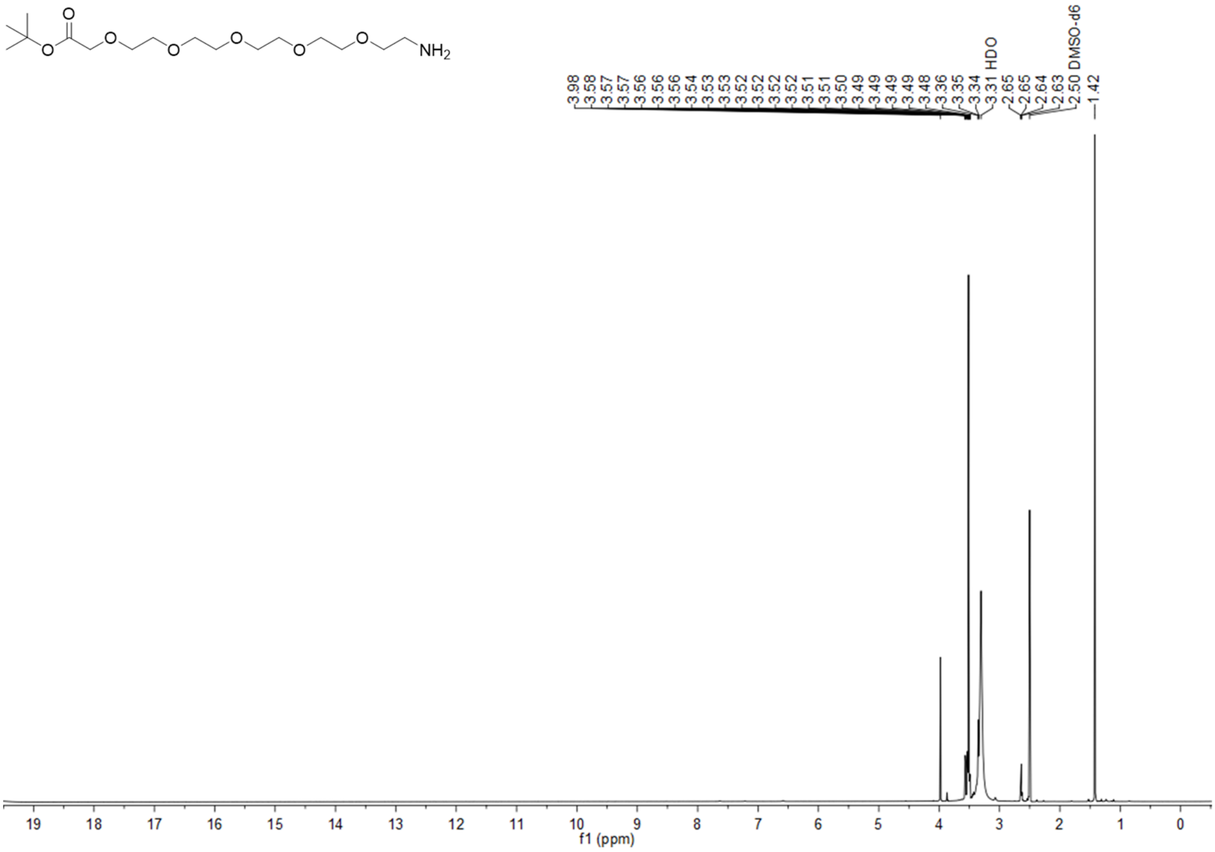

^{13}C-NMR *tert*-Butyl-17-amino-3,6,9,12,15-pentaoxaheptadecanoat in DMSO-d_6

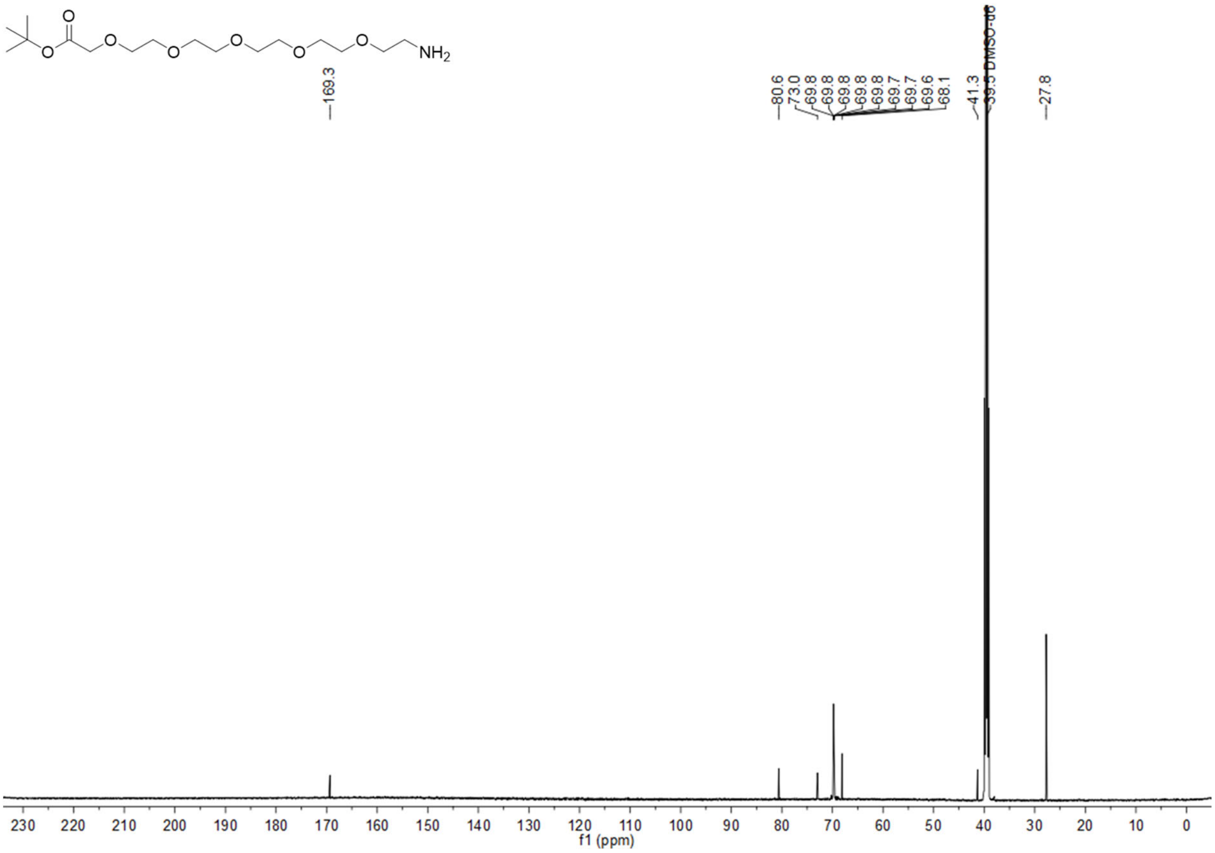

^{1}H-NMR *tert*-Butyl-26-amino-3,6,9,12,15,18,21,24-octaoxahexacosanoat in DMSO-d_6

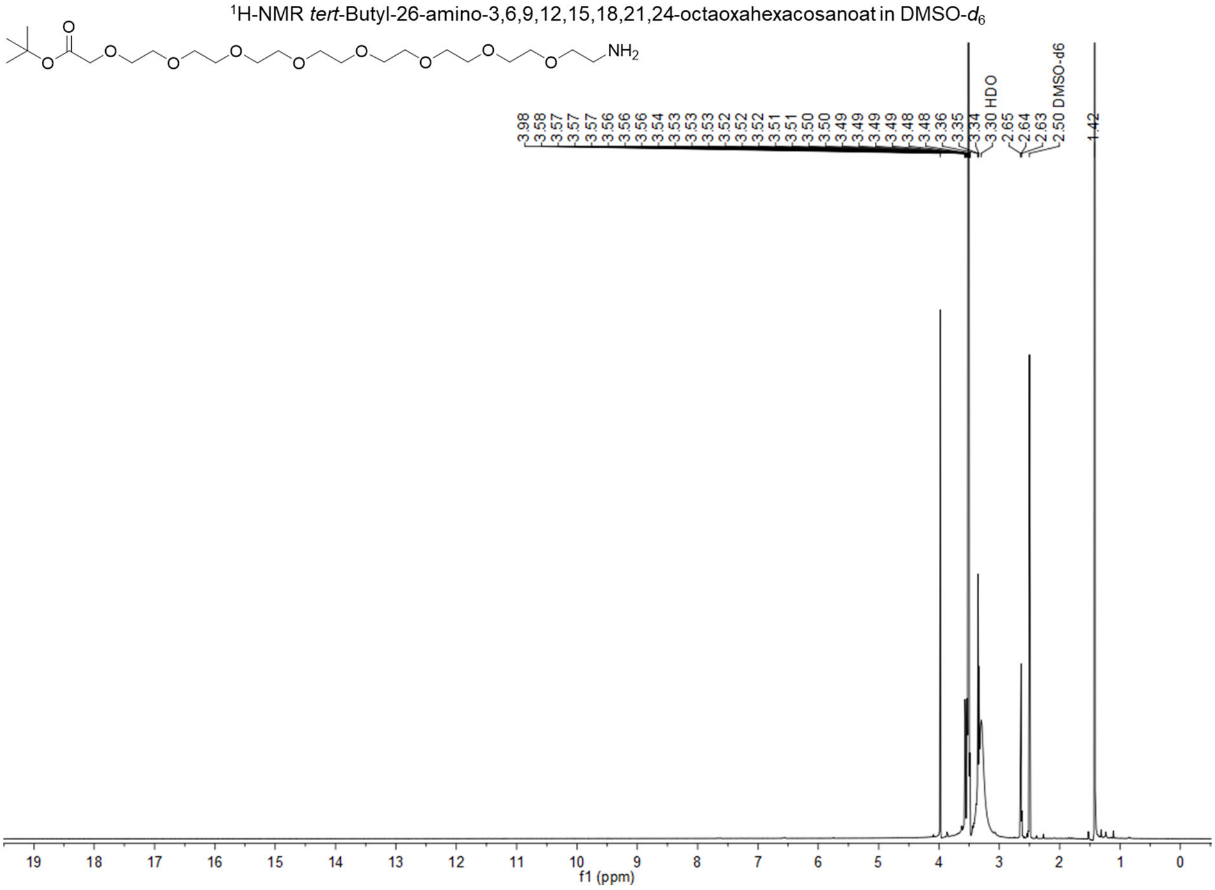

^{13}C-NMR *tert*-Butyl-26-amino-3,6,9,12,15,18,21,24-octaoxahexacosanoat in DMSO-d_6

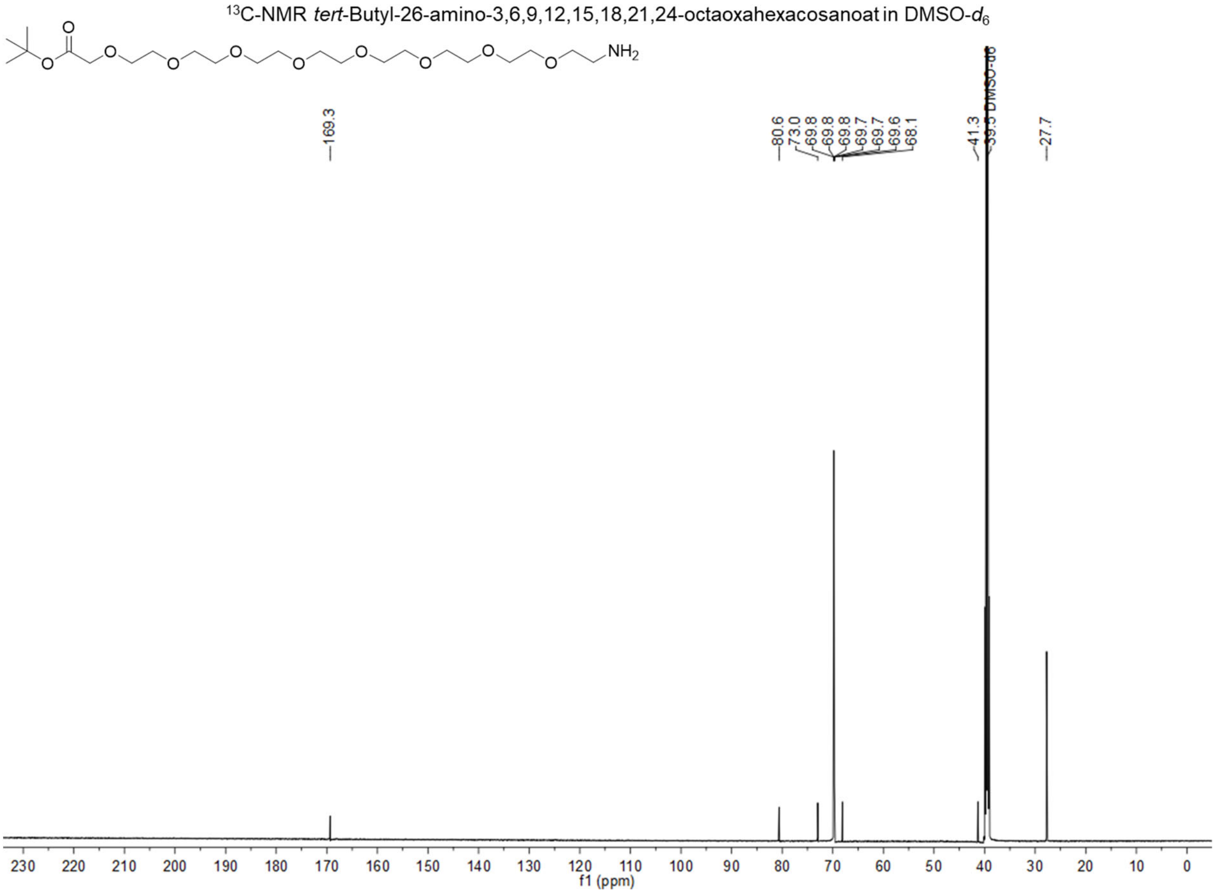

^{1}H-NMR *tert*-Butyl-38-amino-3,6,9,12,15,18,21,24,27,30,33,36-dodecaoxaoctatriacontanoat in DMSO-d_6

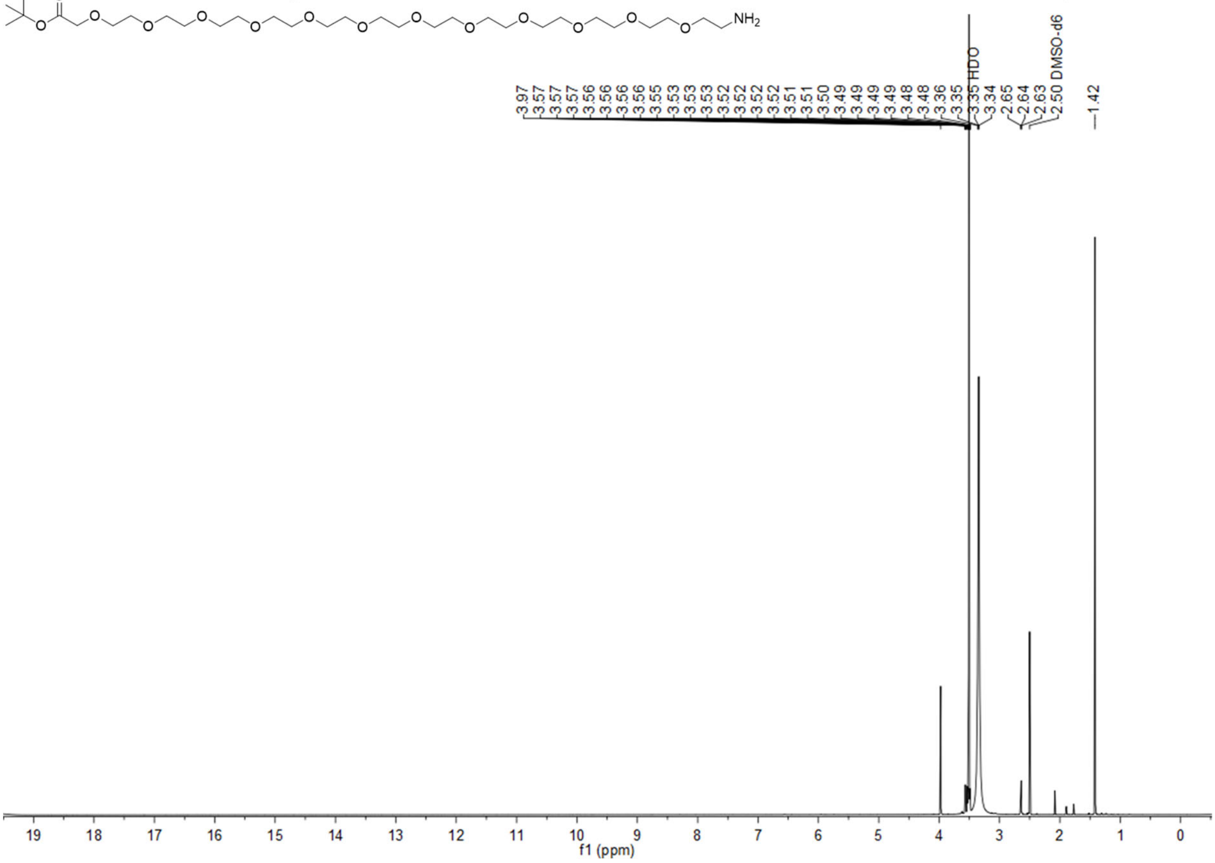

^{13}C-NMR *tert*-Butyl-38-amino-3,6,9,12,15,18,21,24,27,30,33,36-dodecaoxaoctatriacontanoat in DMSO-d_6

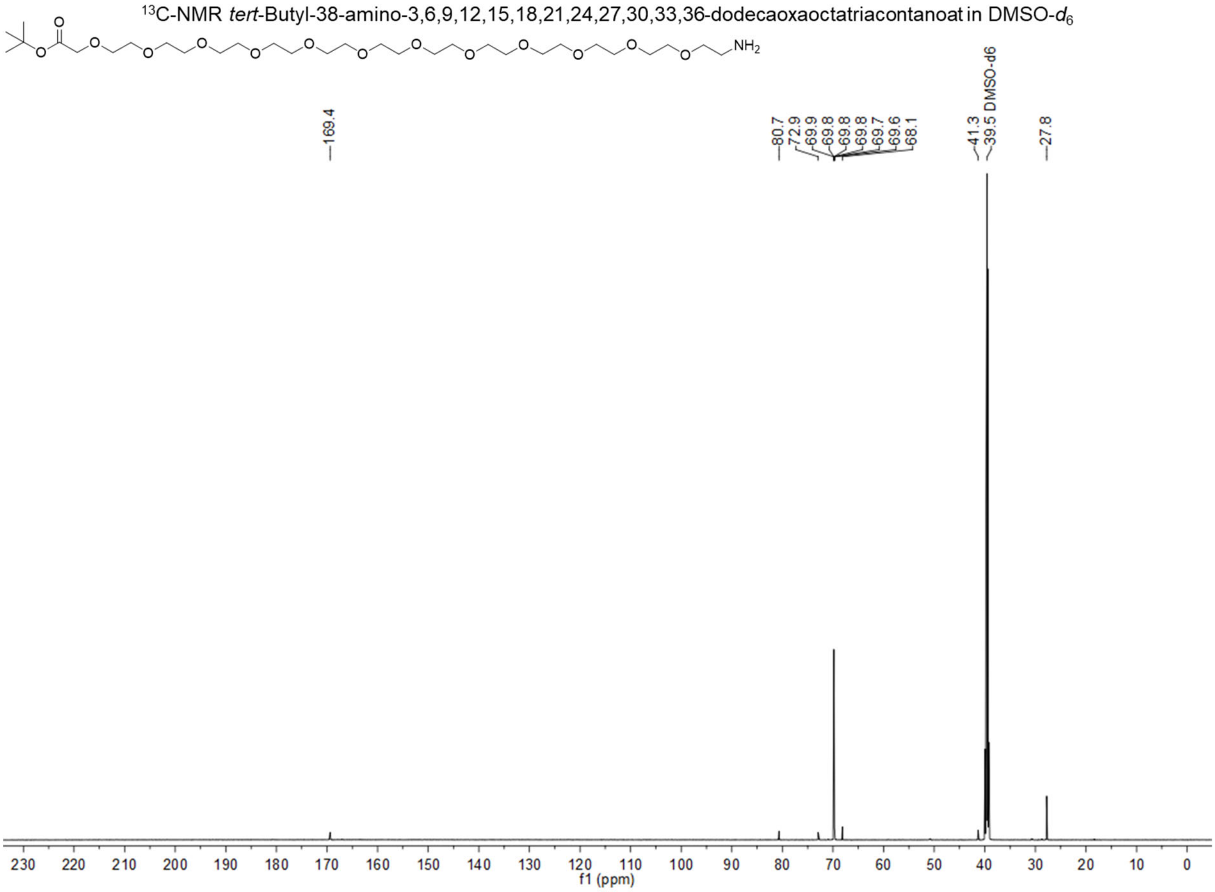

^{1}H-NMR *tert*-Butyl-50-amino-3,6,9,12,15,18,21,24,27,30,33,36,39,42,45,48-hexadecaoxapentacontanoat in DMSO-d_6

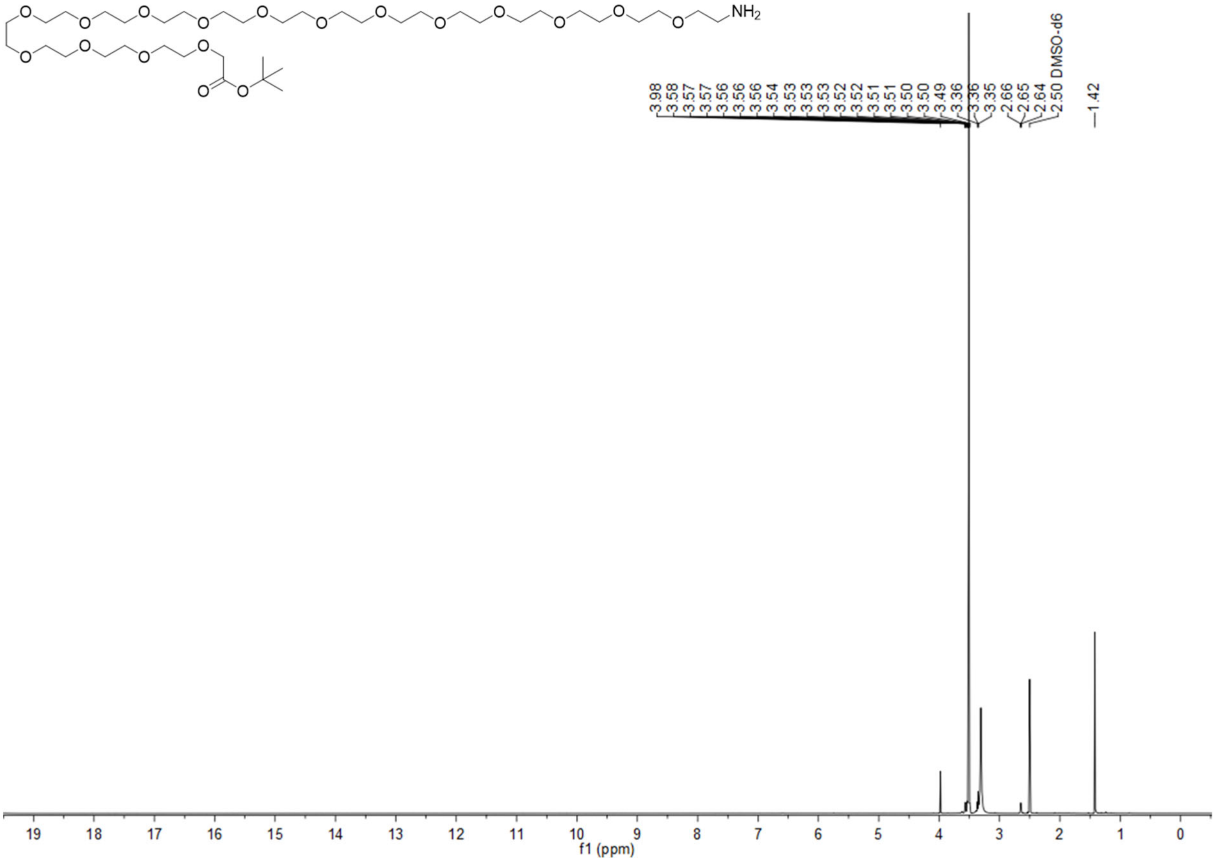

^{13}C-NMR *tert*-Butyl-50-amino-3,6,9,12,15,18,21,24,27,30,33,36,39,42,45,48-hexadecaoxapentacontanoat in DMSO-d_6

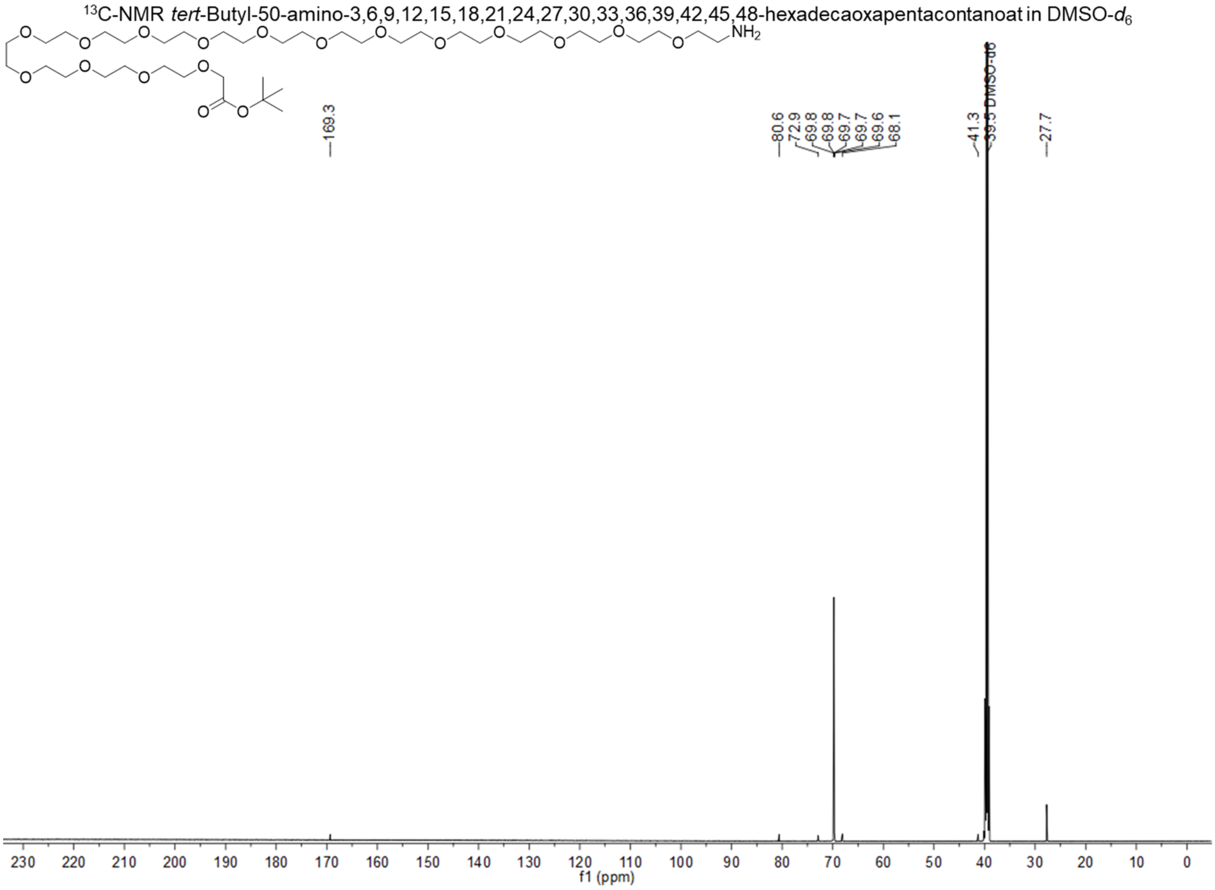

^{1}H-NMR *tert*-Butyl-62-amino-3,6,9,12,15,18,21,24,27,30,33,36,39,42,45,48,51,54,57,60-icosaoxadohexacontanoat in DMSO-d_6

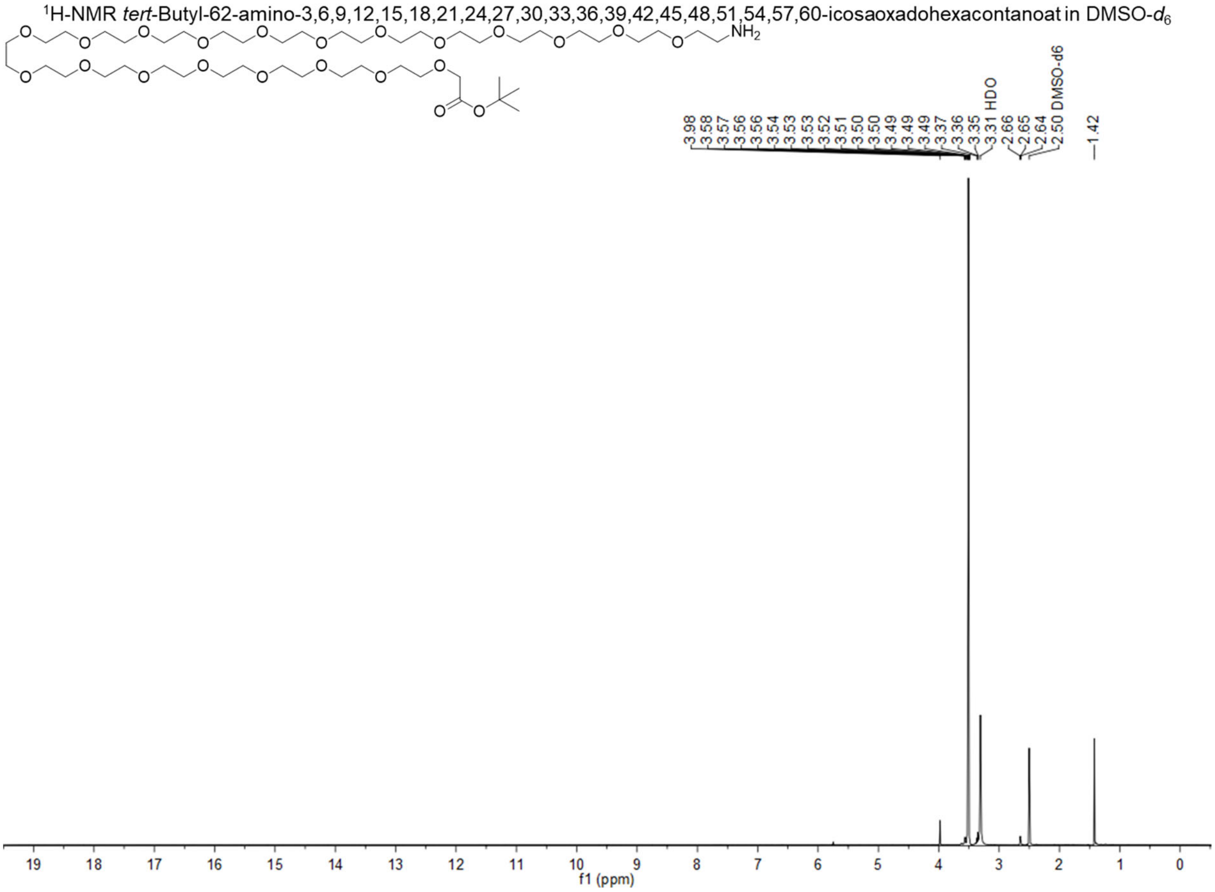

^{13}C-NMR *tert*-Butyl-62-amino-3,6,9,12,15,18,21,24,27,30,33,36,39,42,45,48,51,54,57,60-icosaoxadohexacontanoat in DMSO-d_6

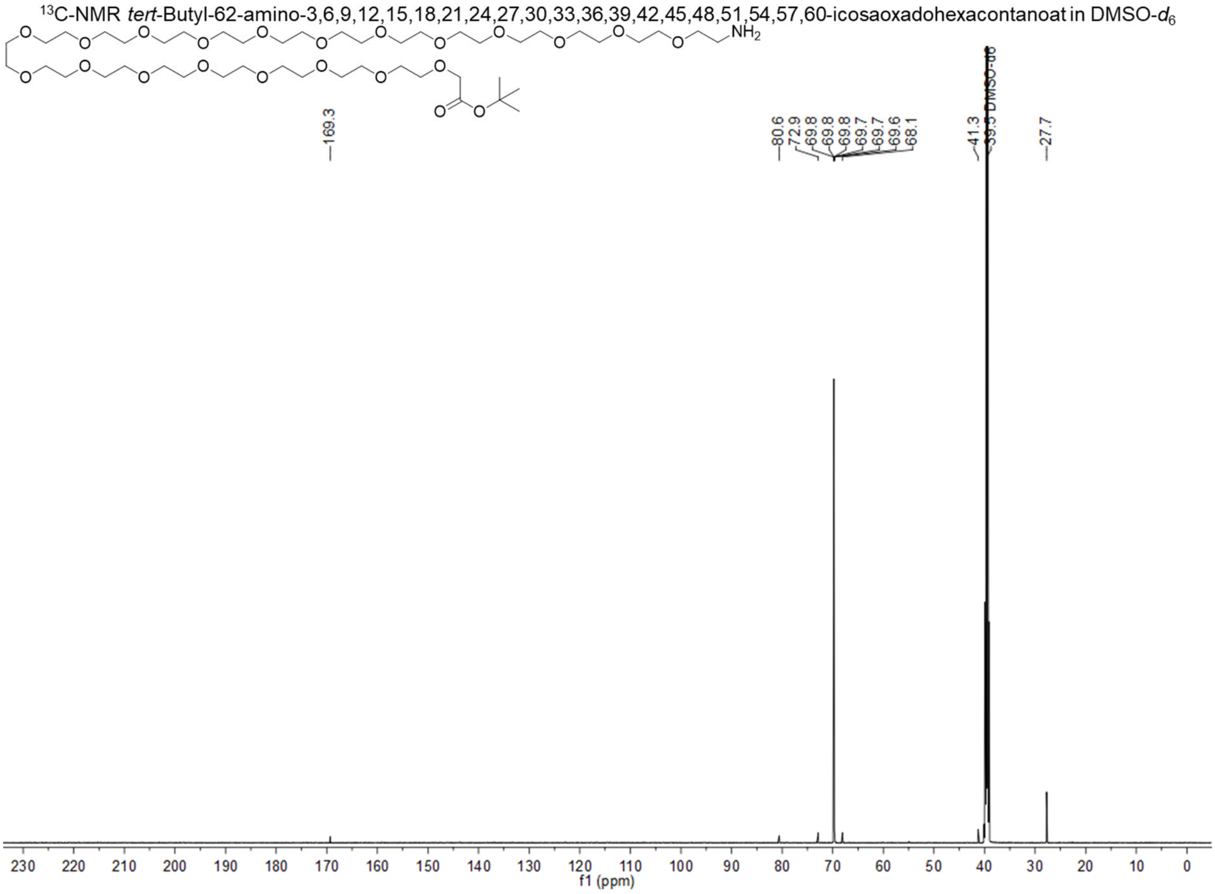

Supplement: Supplementary file 1 — Supporting Information [file ANIE-61-0-s001.pdf]
